# Supplementary figures and images for: Ubiquitin ligase and signalling hub MYCBP2 is required for efficient EPHB2 tyrosine kinase receptor function
Source: eLife. 2024 Jan 30;12:RP89176. doi: 10.7554/eLife.89176 (PMC10945567; doi:10.7554/eLife.89176)

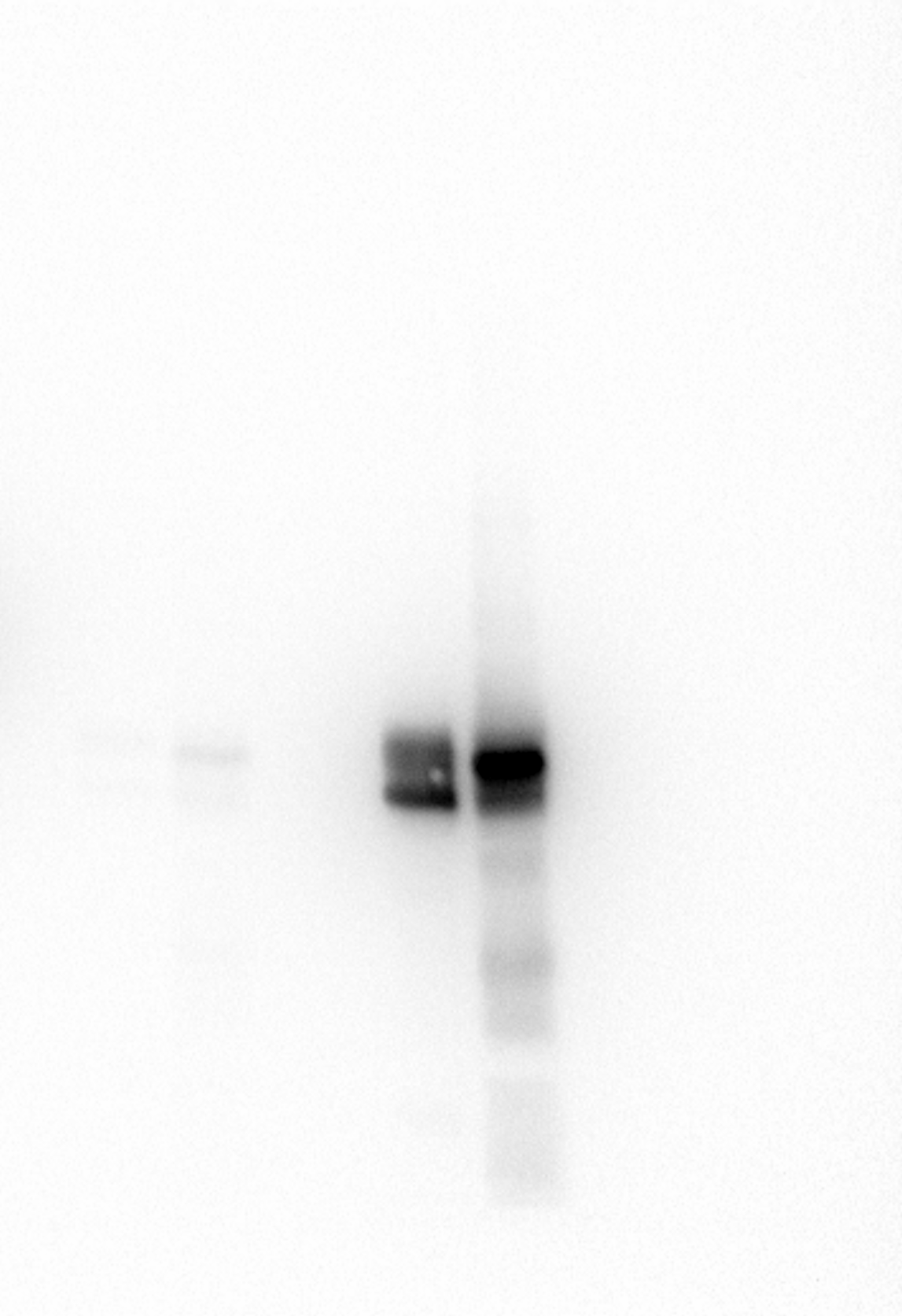

Supplement: Figure 1—source data 1. [file elife-89176-fig1-data1.zip › Figure 1 - source data 1/Figure 1C - FLAG IP.tif]

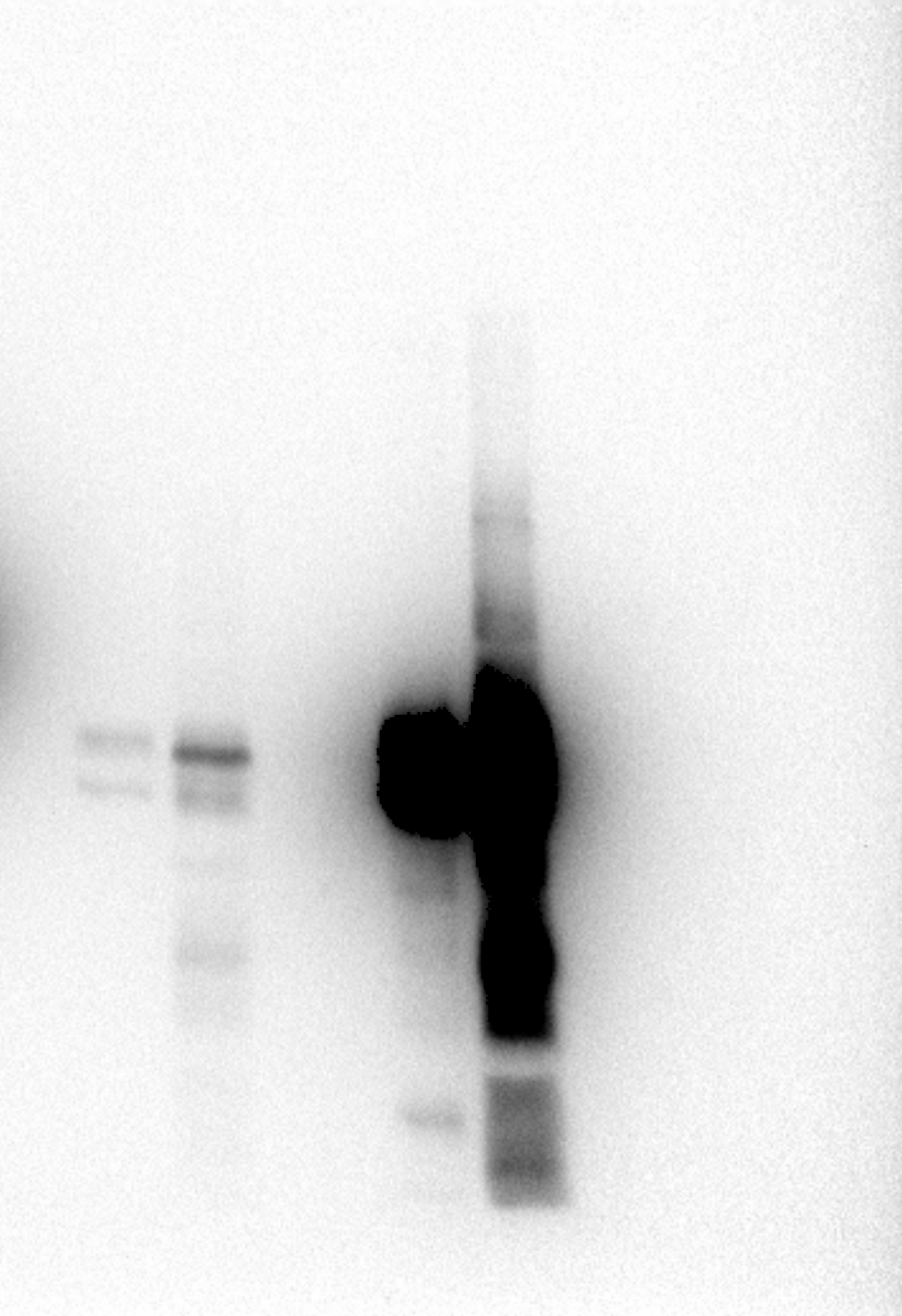

Supplement: Figure 1—source data 1. [file elife-89176-fig1-data1.zip › Figure 1 - source data 1/Figure 1C - FLAG Lysate.tif]

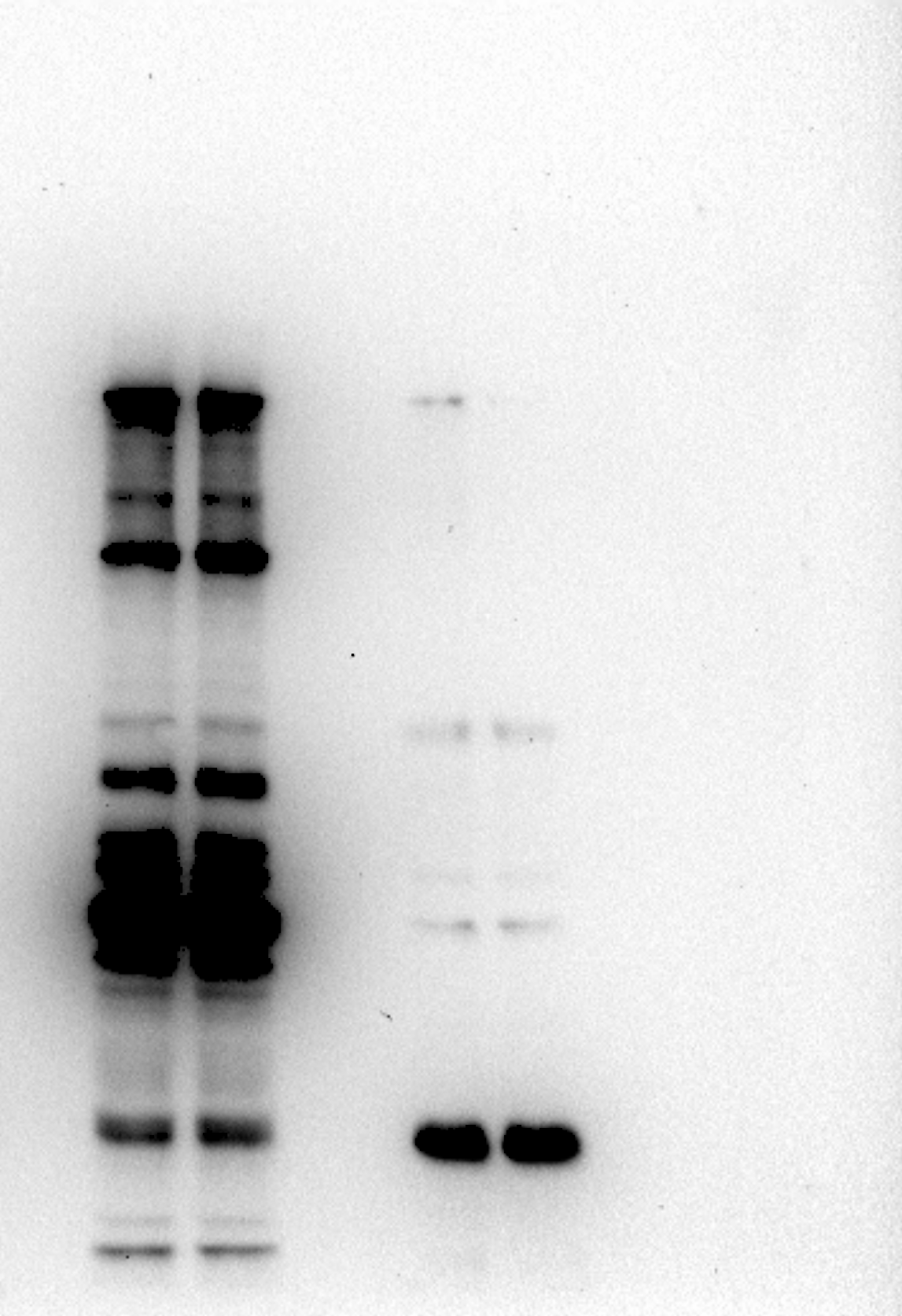

Supplement: Figure 1—source data 1. [file elife-89176-fig1-data1.zip › Figure 1 - source data 1/Figure 1C - MYCBP2 IP.tif]

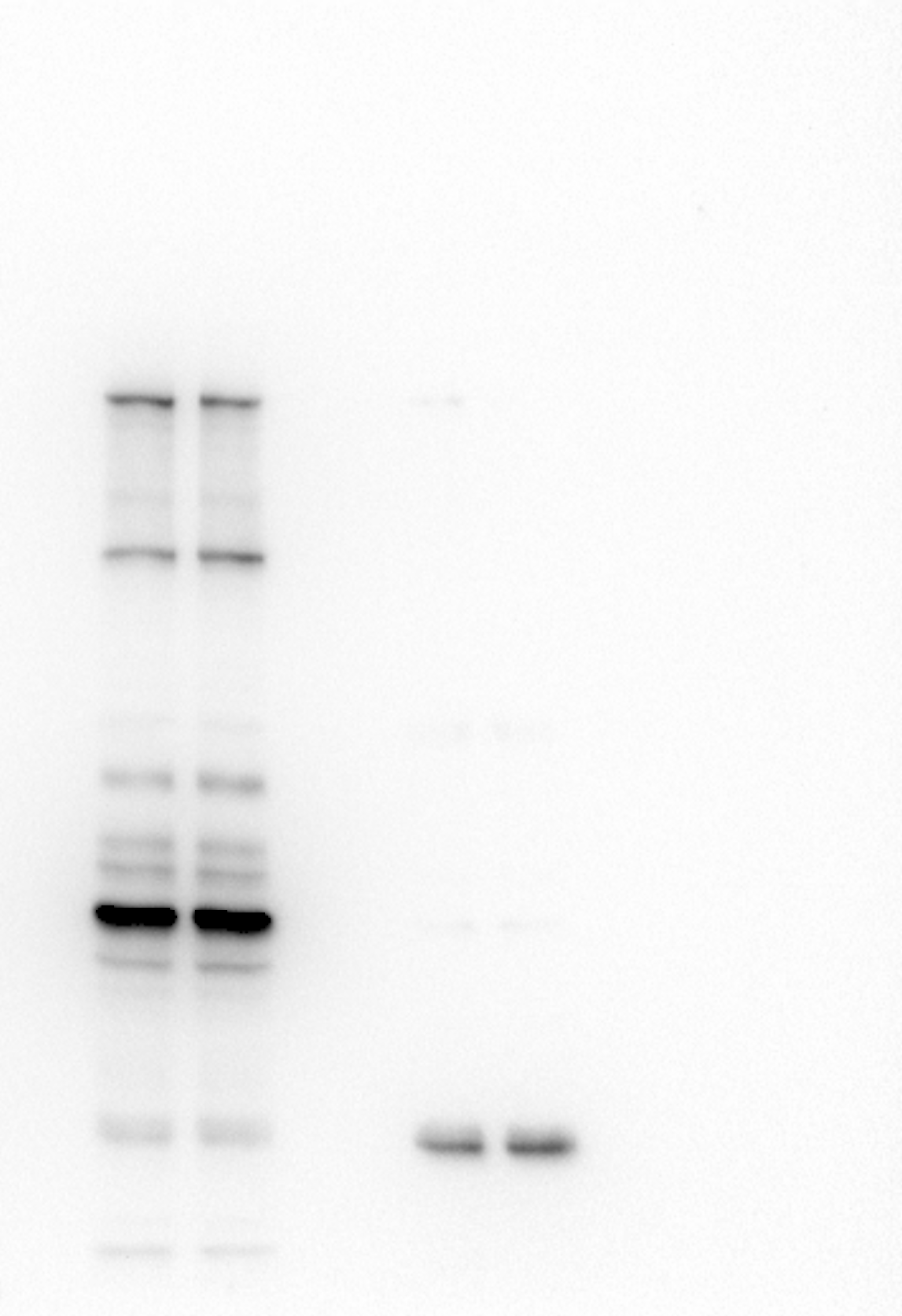

Supplement: Figure 1—source data 1. [file elife-89176-fig1-data1.zip › Figure 1 - source data 1/Figure 1C - MYCBP2 Lysate.tif]

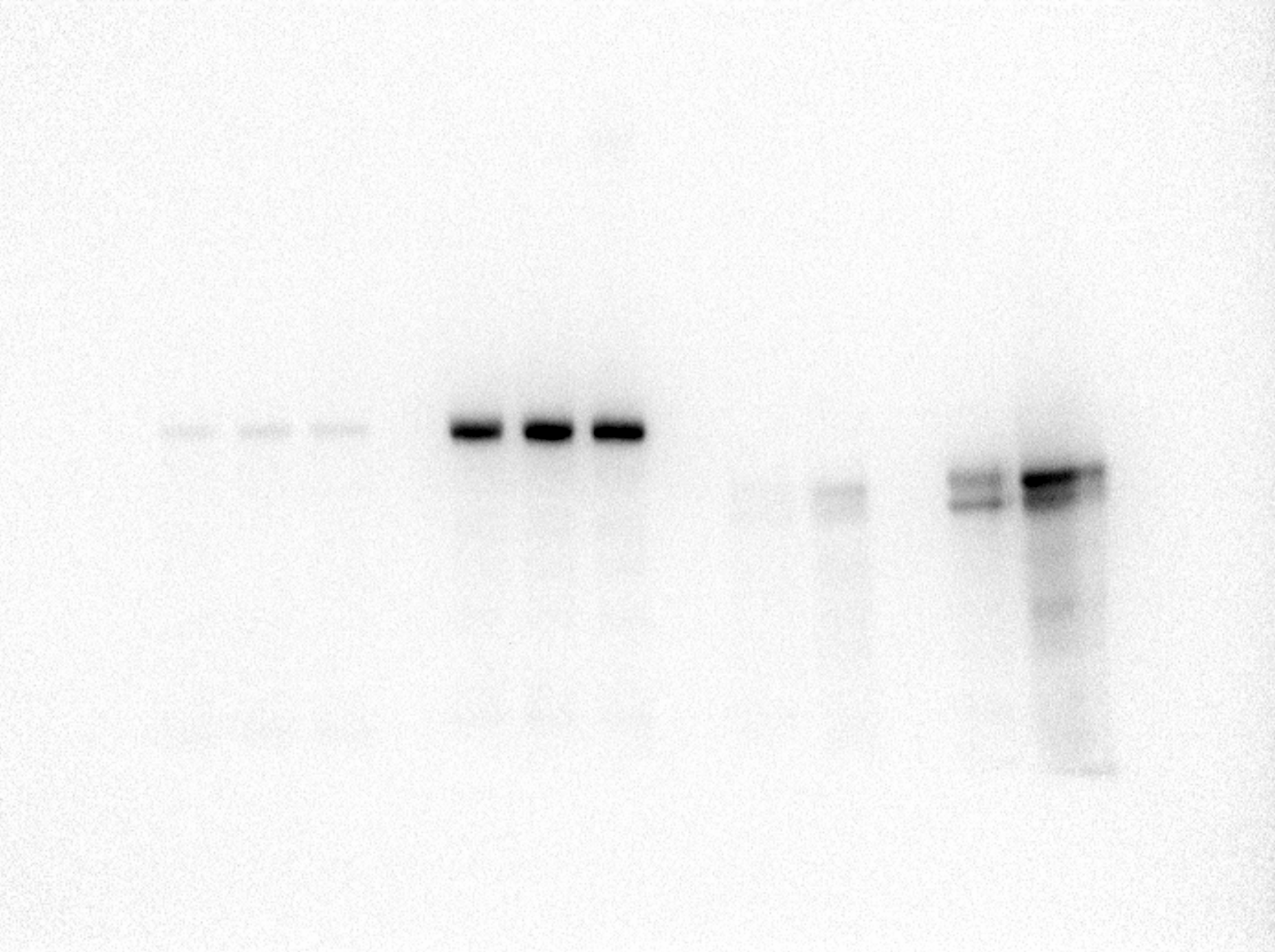

Supplement: Figure 1—source data 1. [file elife-89176-fig1-data1.zip › Figure 1 - source data 1/Figure 1D - EPHB2-FLAG IP.tif]

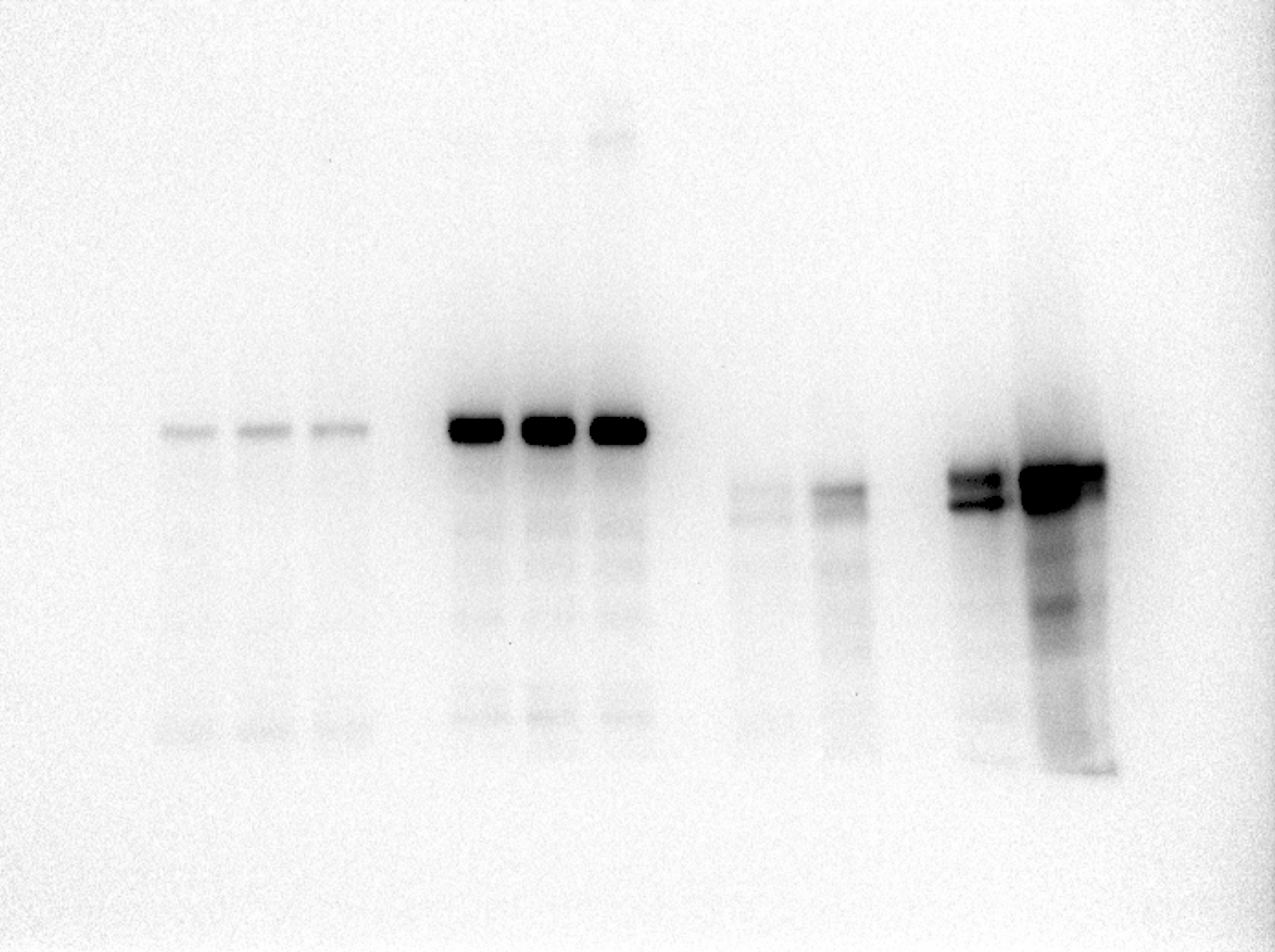

Supplement: Figure 1—source data 1. [file elife-89176-fig1-data1.zip › Figure 1 - source data 1/Figure 1D - EPHB2-FLAG Lysate.tif]

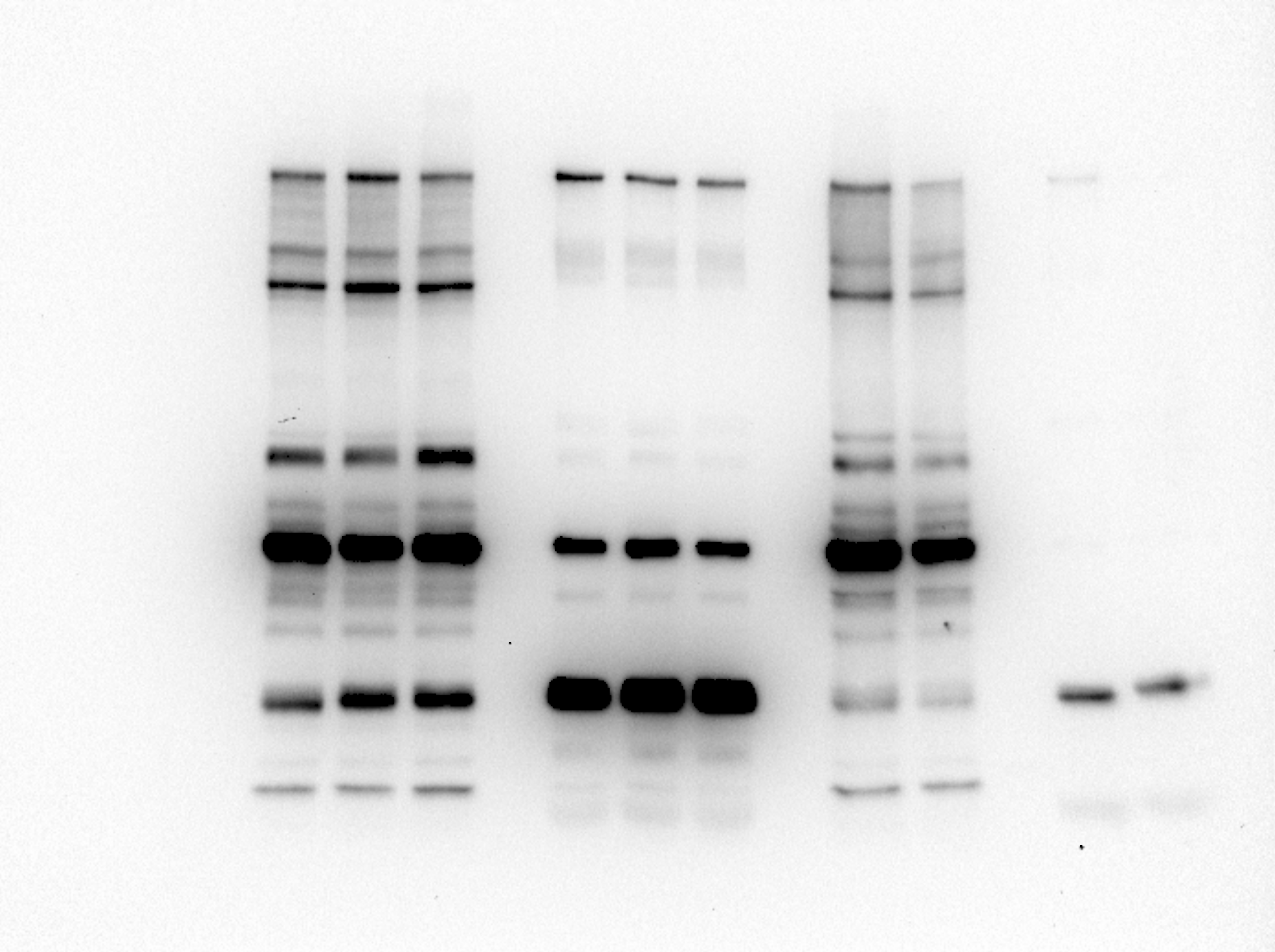

Supplement: Figure 1—source data 1. [file elife-89176-fig1-data1.zip › Figure 1 - source data 1/Figure 1D - MYCBP2 IP Lysate.tif]

Figure 1C

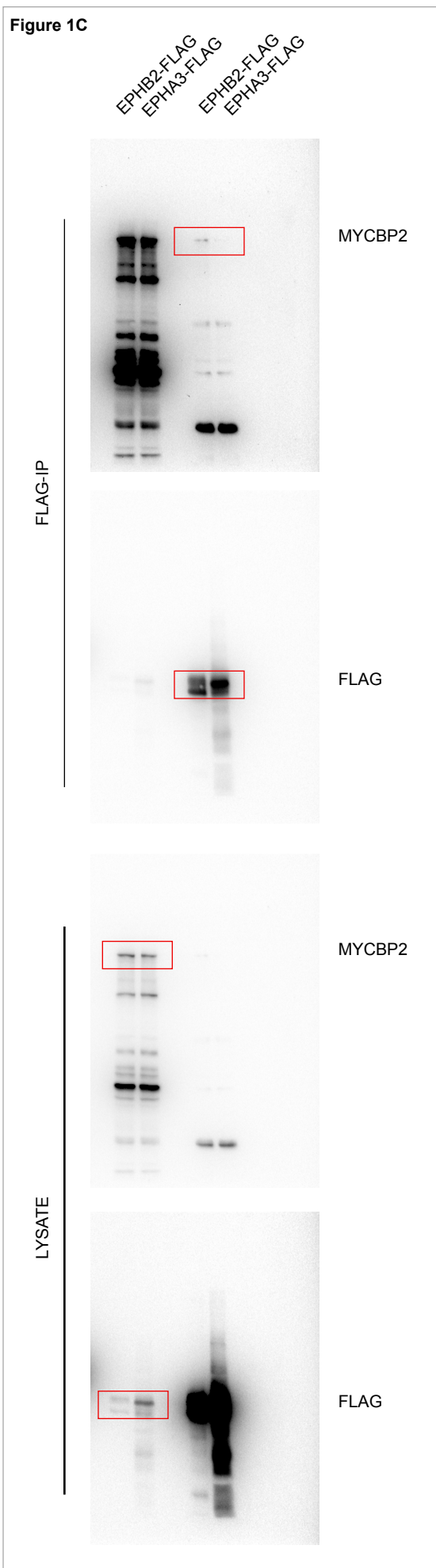

Figure 1D

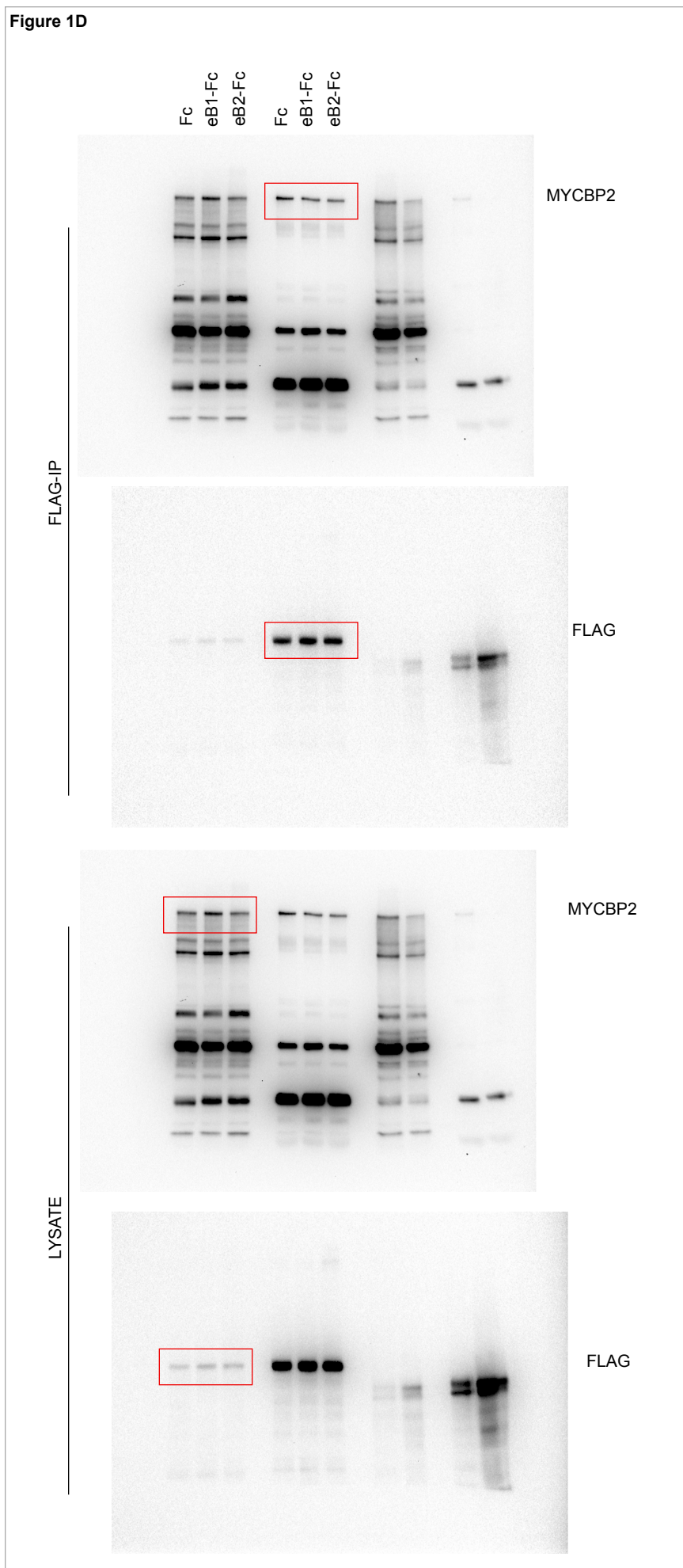

Supplement: Figure 1—source data 1. [file elife-89176-fig1-data1.zip › Figure 1 - source data 1/Figure 1-Source data 1.pdf]

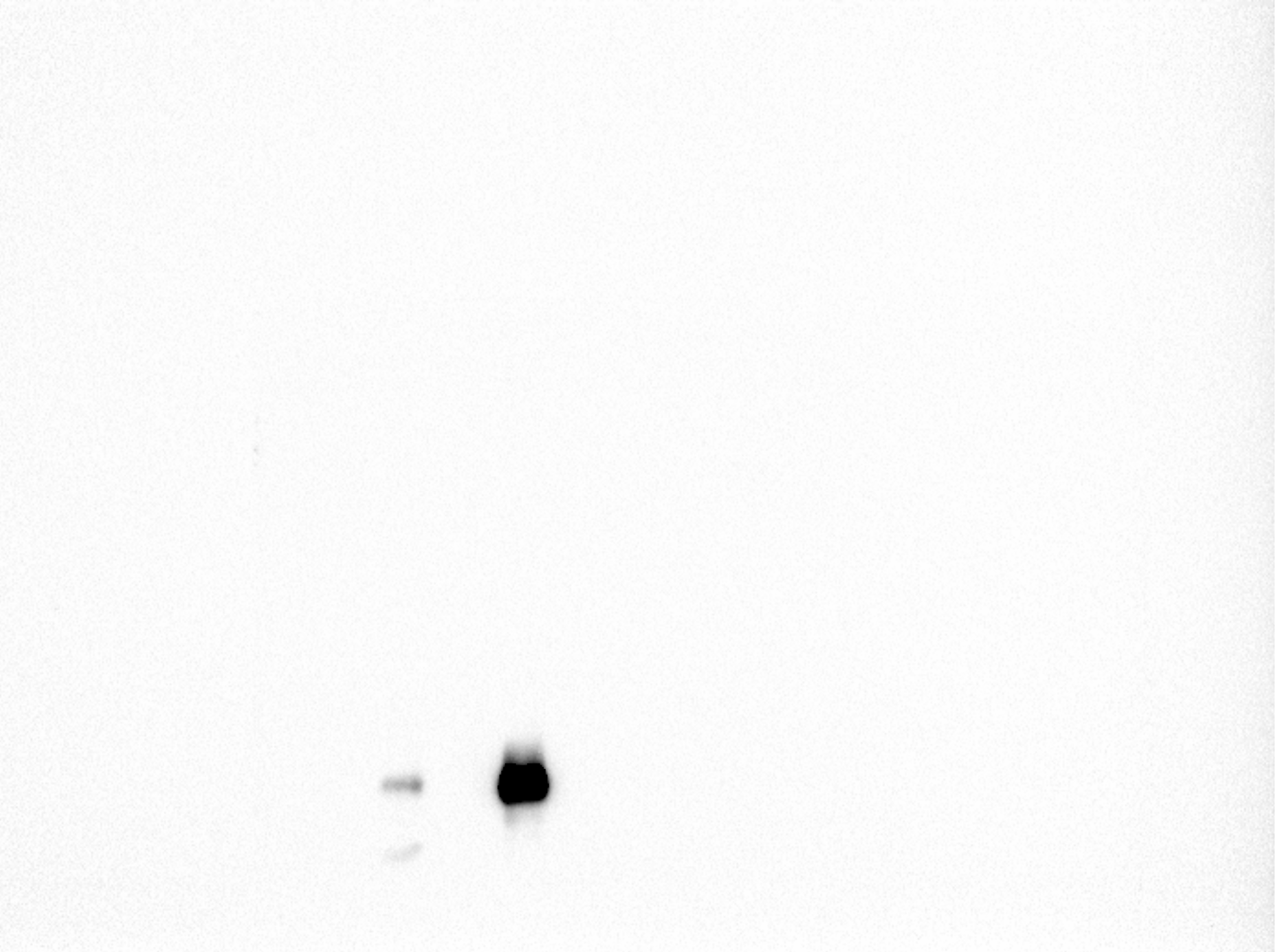

Supplement: Figure 1—source data 2. [file elife-89176-fig1-data2.zip › Figure 1 - source data 2/Figure 1F - EPHB2.tif]

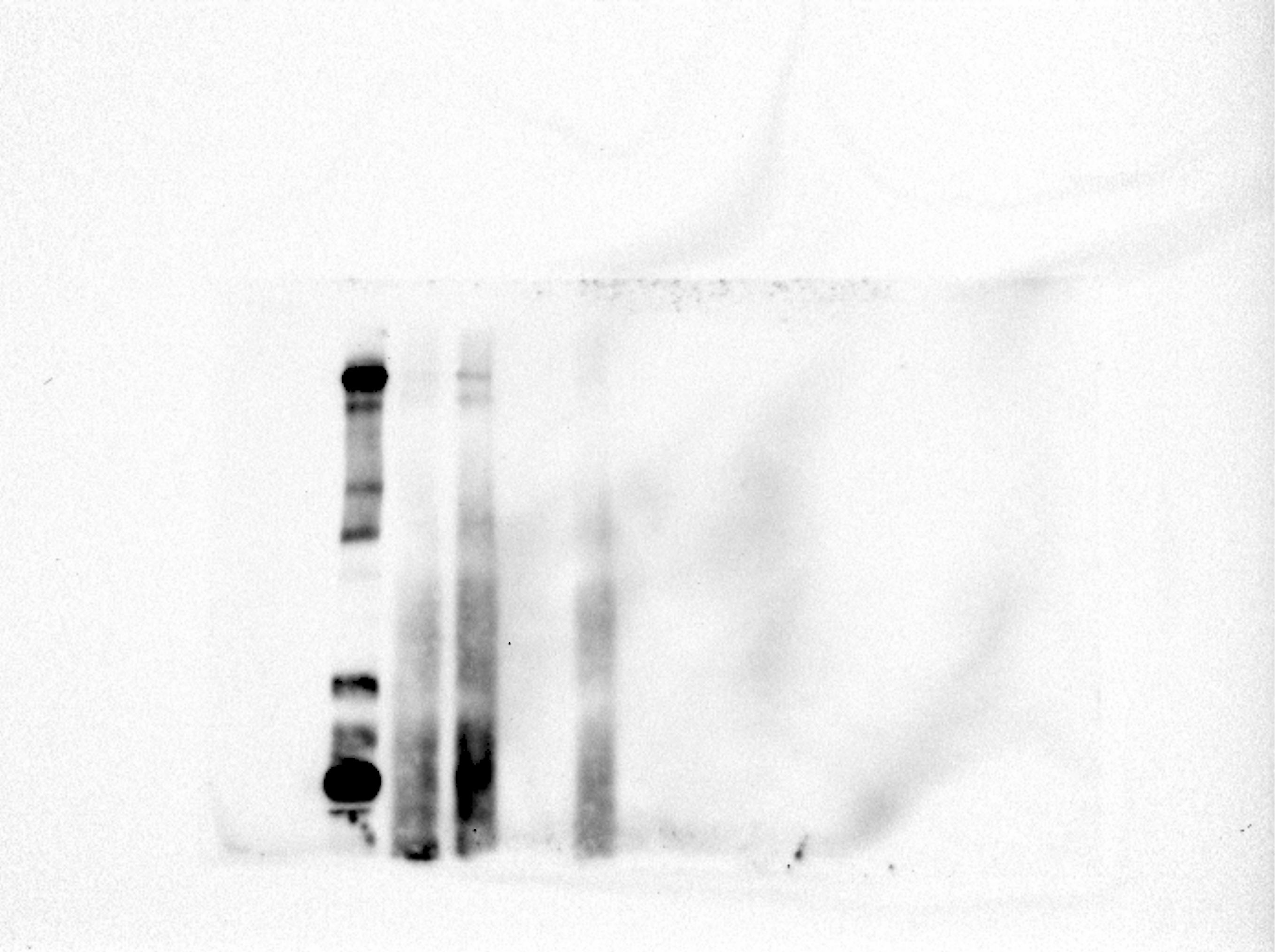

Supplement: Figure 1—source data 2. [file elife-89176-fig1-data2.zip › Figure 1 - source data 2/Figure 1F - MYCBP2.tif]

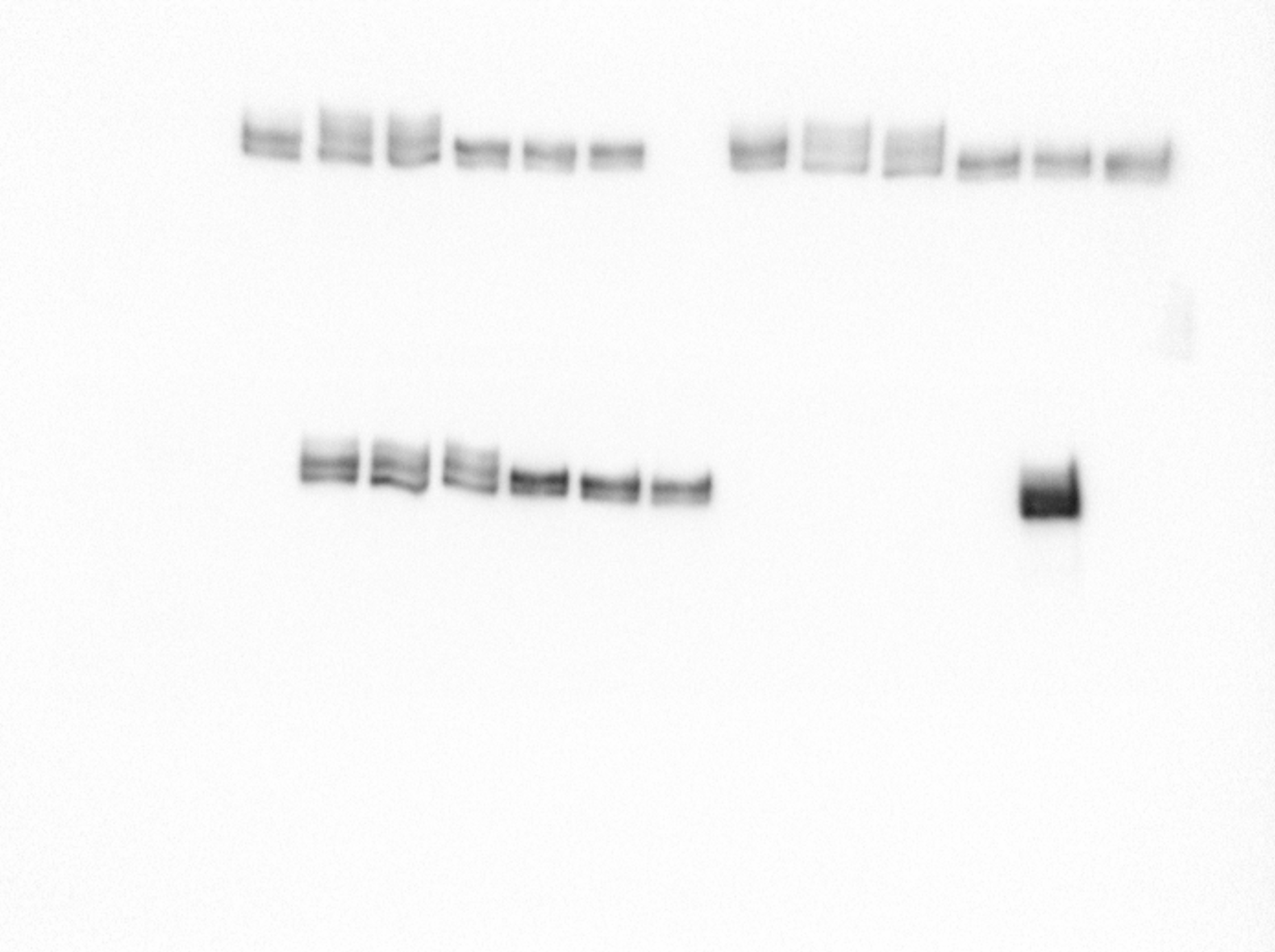

Supplement: Figure 1—source data 2. [file elife-89176-fig1-data2.zip › Figure 1 - source data 2/Figure 1G - EPHB2 .tif]

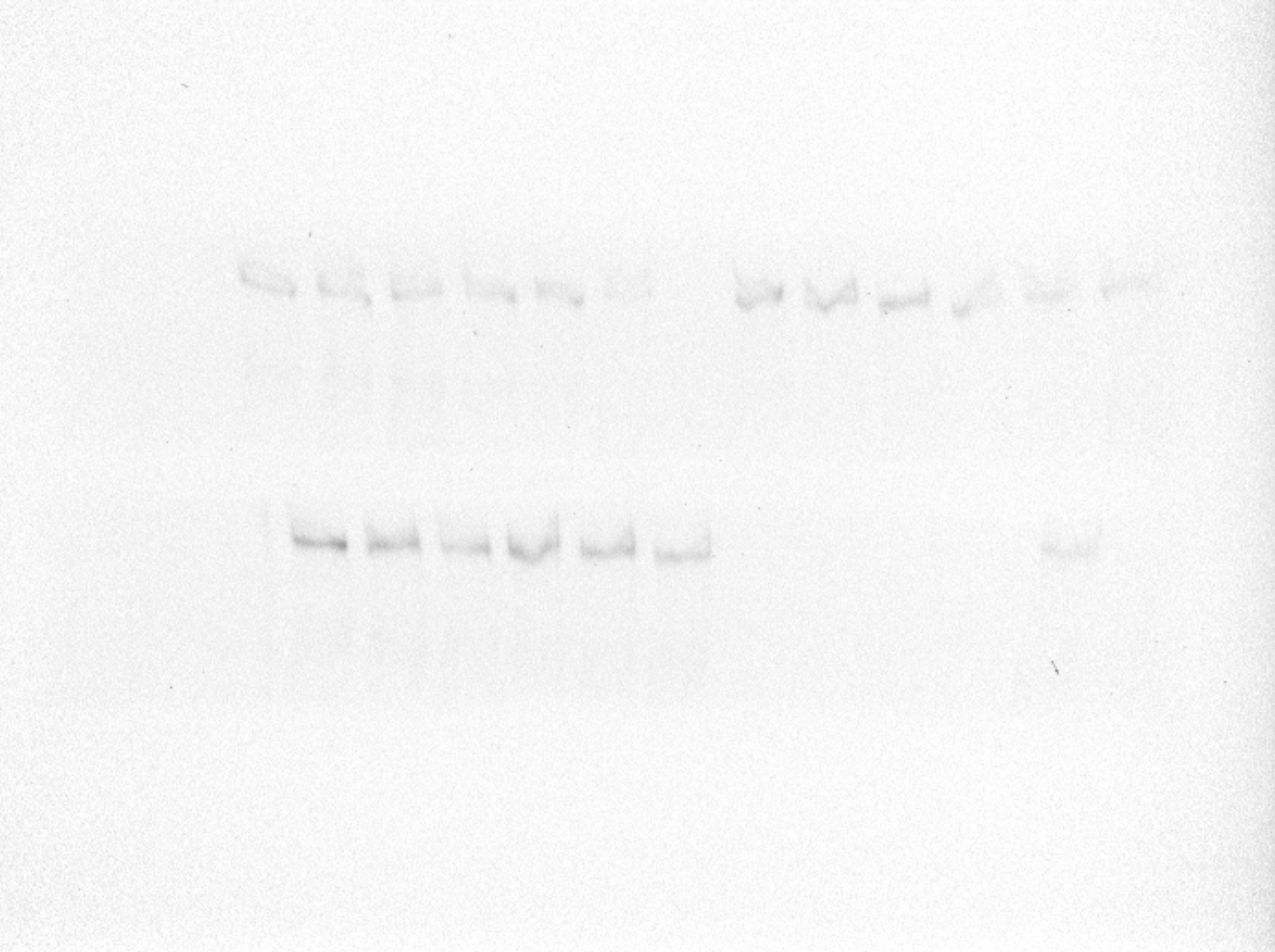

Supplement: Figure 1—source data 2. [file elife-89176-fig1-data2.zip › Figure 1 - source data 2/Figure 1G - MYCBP2.tif]

Figure 1F

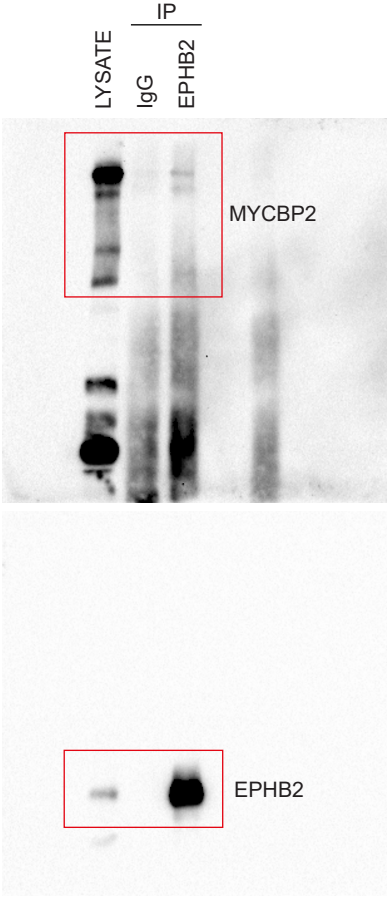

Figure 1G

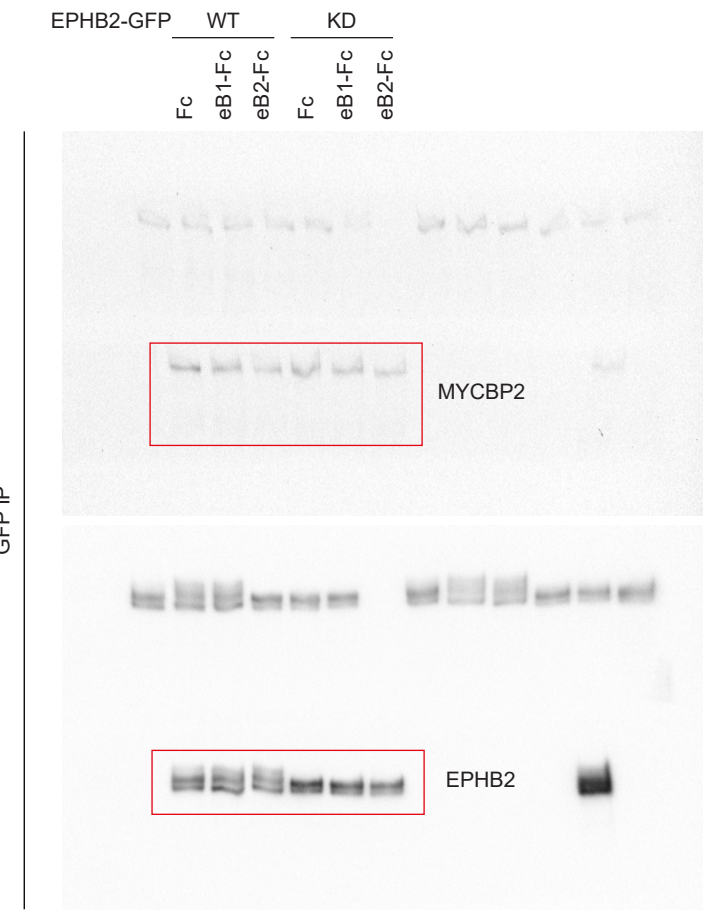

Supplement: Figure 1—source data 2. [file elife-89176-fig1-data2.zip › Figure 1 - source data 2/Figure 1-source data 2.pdf]

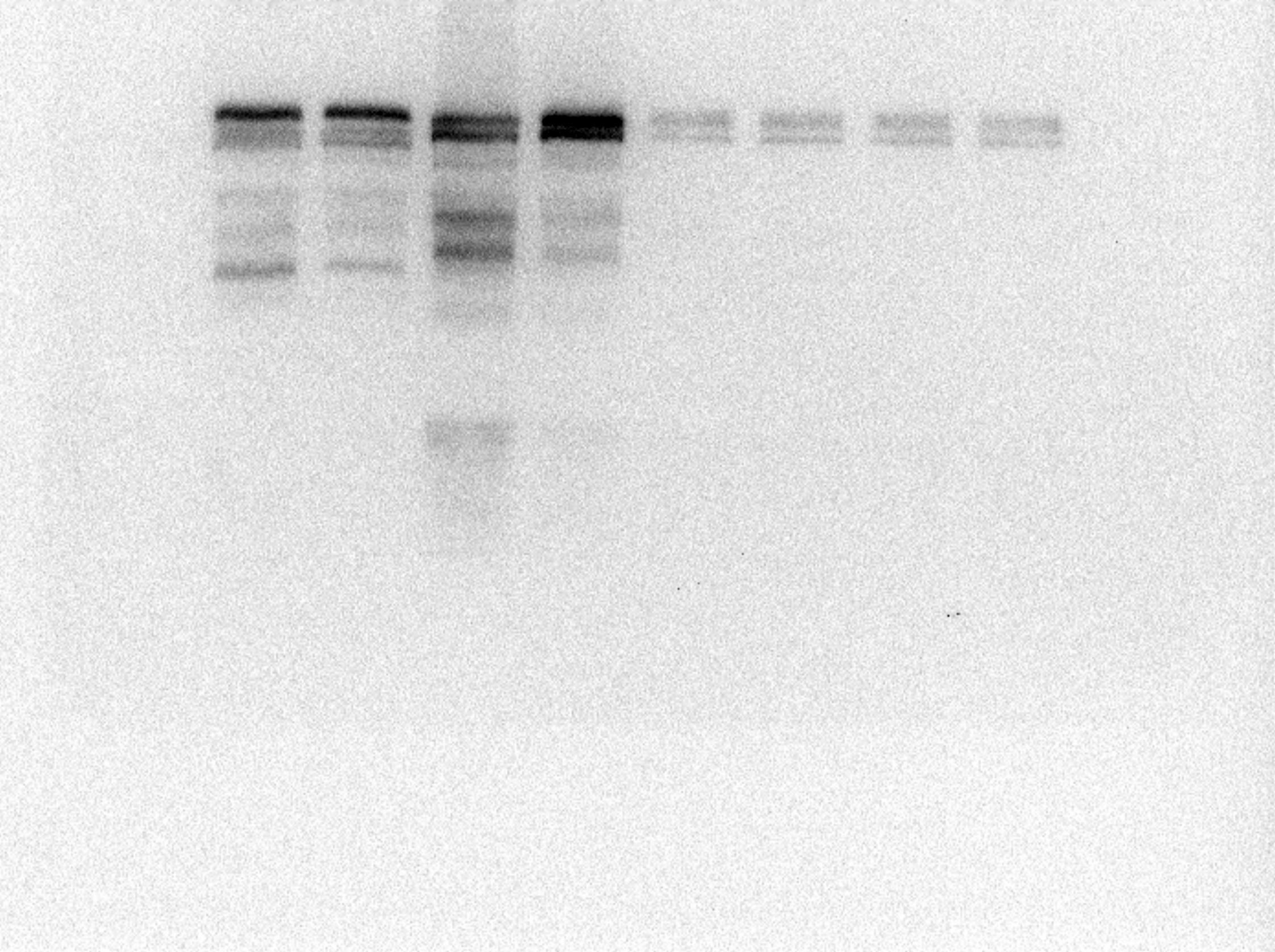

Supplement: Figure 2—source data 1. [file elife-89176-fig2-data1.zip › Figure 2 - source data 1/Figure 2A - FLAG IP.tif]

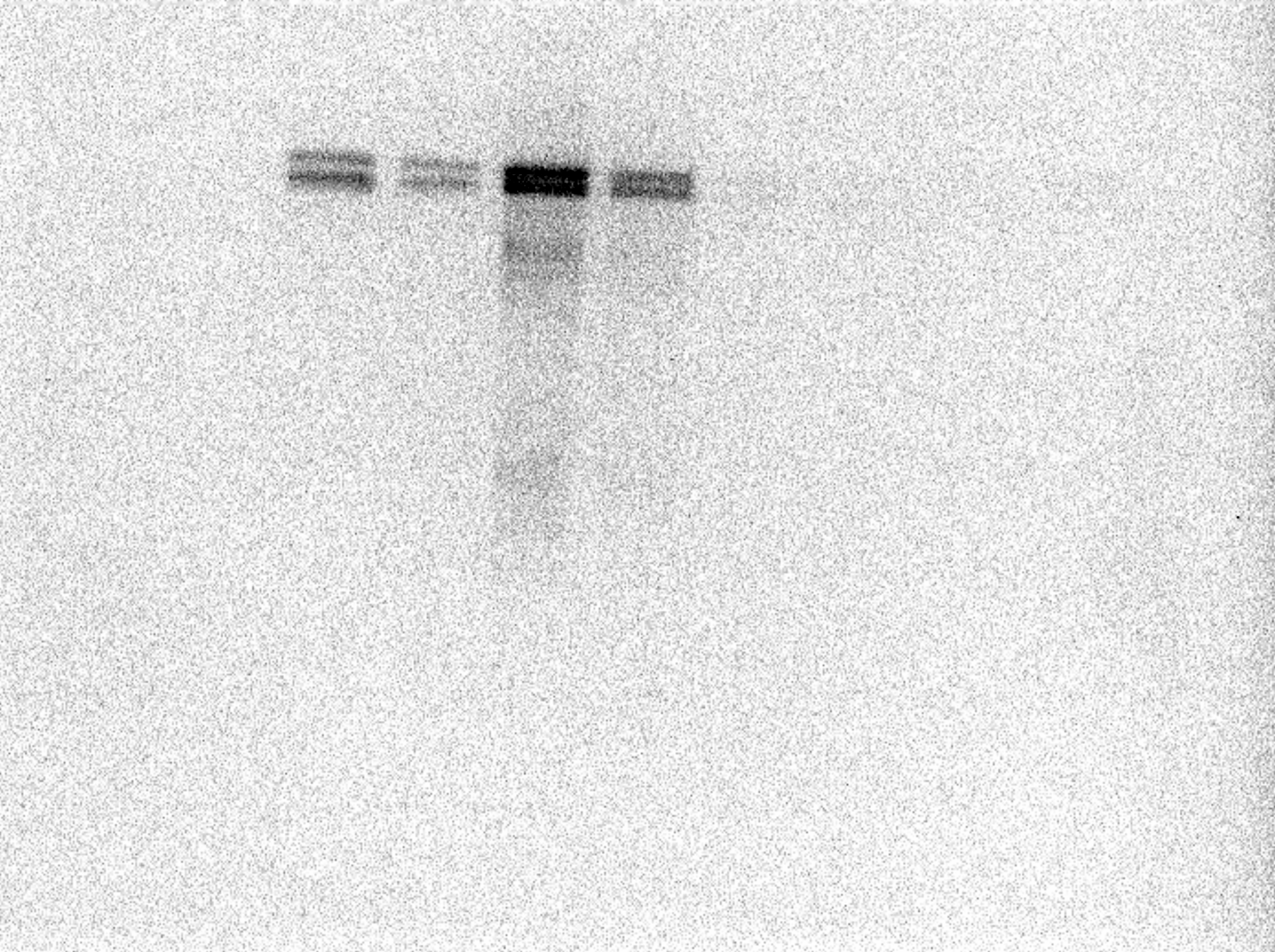

Supplement: Figure 2—source data 1. [file elife-89176-fig2-data1.zip › Figure 2 - source data 1/Figure 2A - FLAG Lysate.tif]

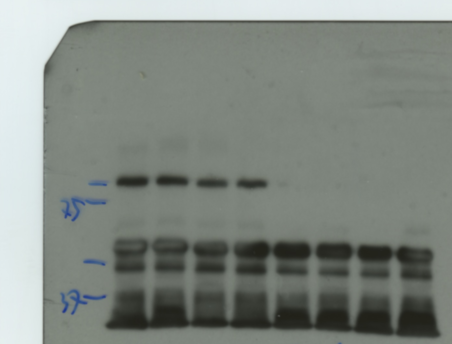

Supplement: Figure 2—source data 1. [file elife-89176-fig2-data1.zip › Figure 2 - source data 1/Figure 2A - MYC IP.tif]

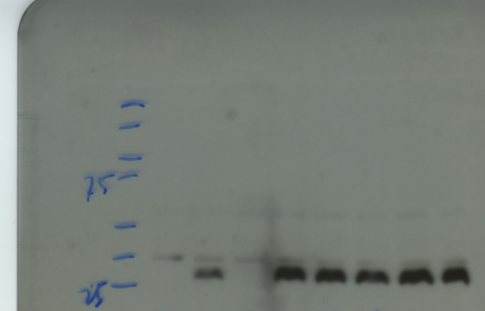

Supplement: Figure 2—source data 1. [file elife-89176-fig2-data1.zip › Figure 2 - source data 1/Figure 2A - MYC Lysate.tif]

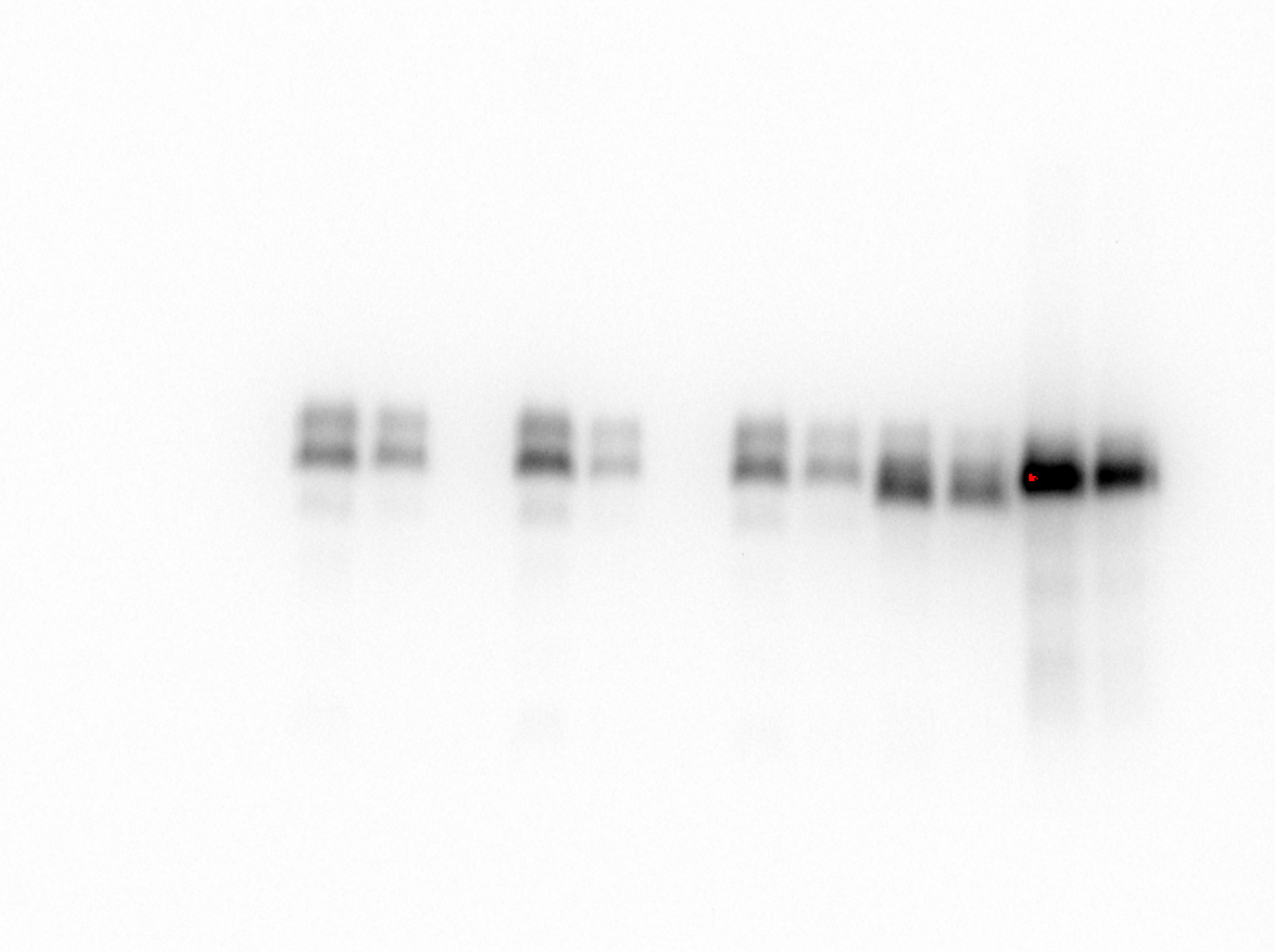

Supplement: Figure 2—source data 1. [file elife-89176-fig2-data1.zip › Figure 2 - source data 1/Figure 2B - FLAG IP.tif]

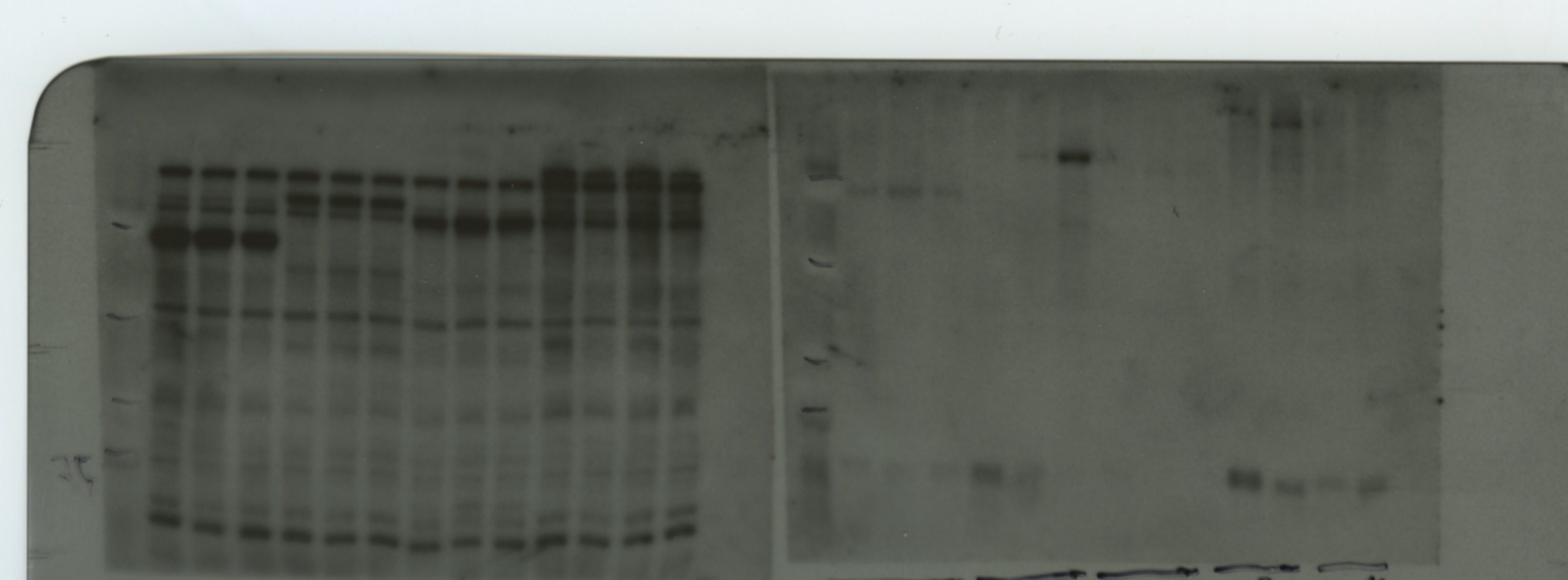

Supplement: Figure 2—source data 1. [file elife-89176-fig2-data1.zip › Figure 2 - source data 1/Figure 2B - GFP IP and Lysate.tif]

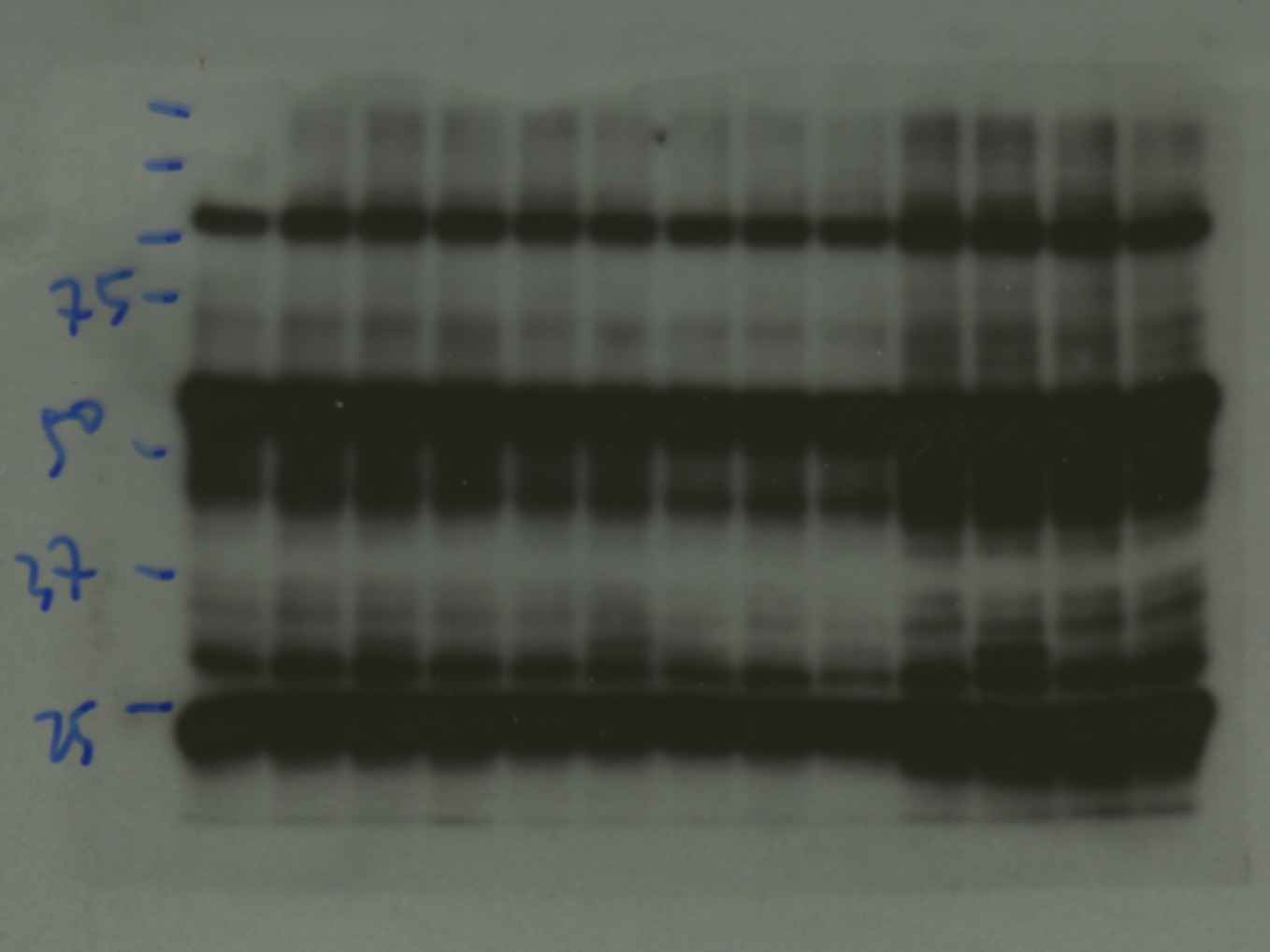

Supplement: Figure 2—source data 1. [file elife-89176-fig2-data1.zip › Figure 2 - source data 1/Figure 2B - MYC IP.tif]

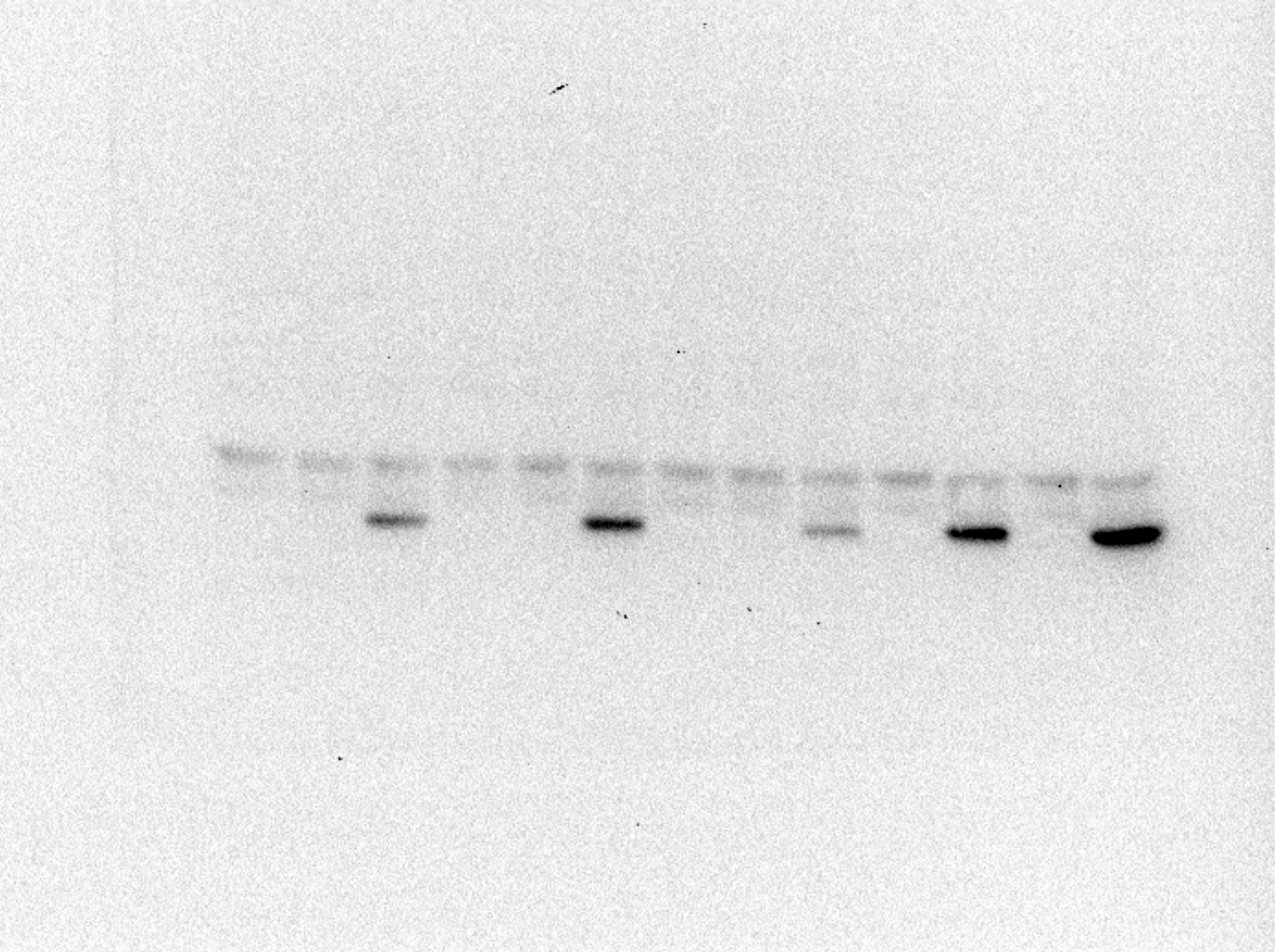

Supplement: Figure 2—source data 1. [file elife-89176-fig2-data1.zip › Figure 2 - source data 1/Figure 2B - MYC Lysate.tif]

Figure 2A

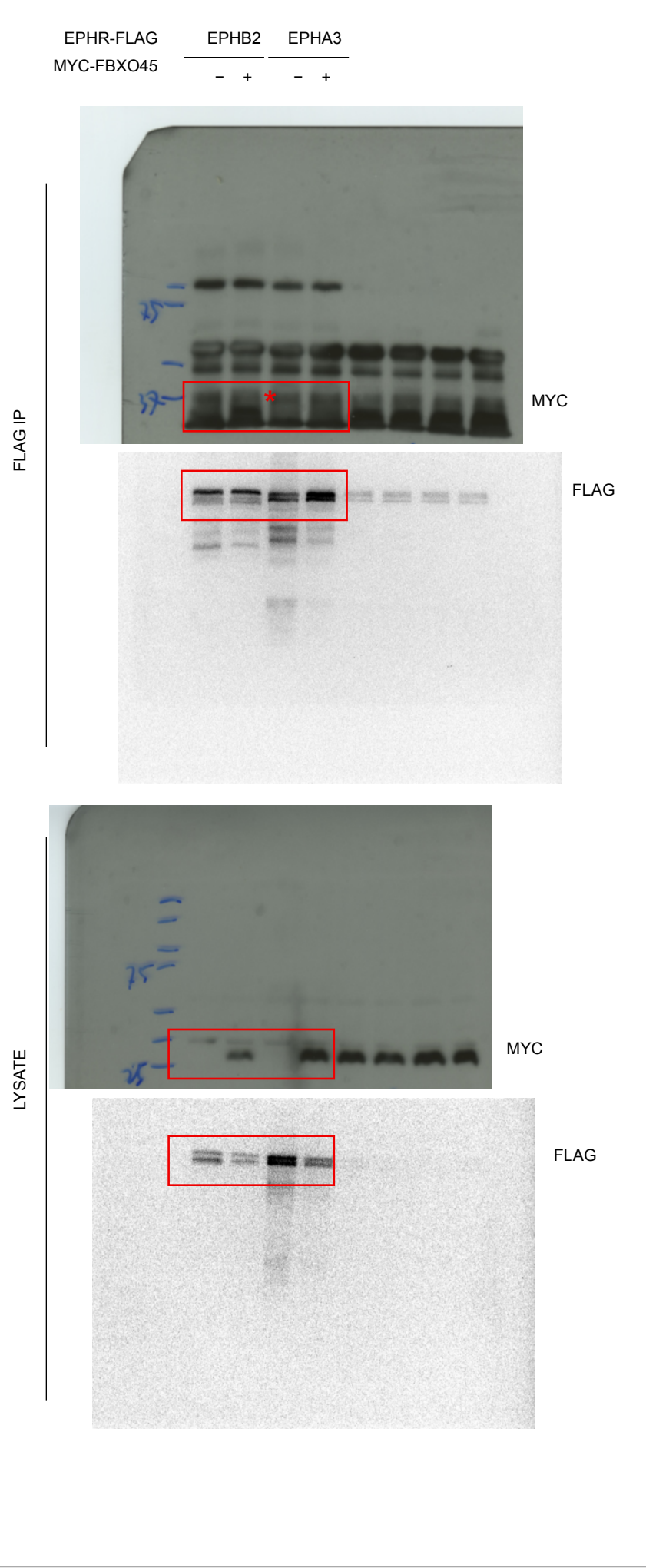

Figure 2B

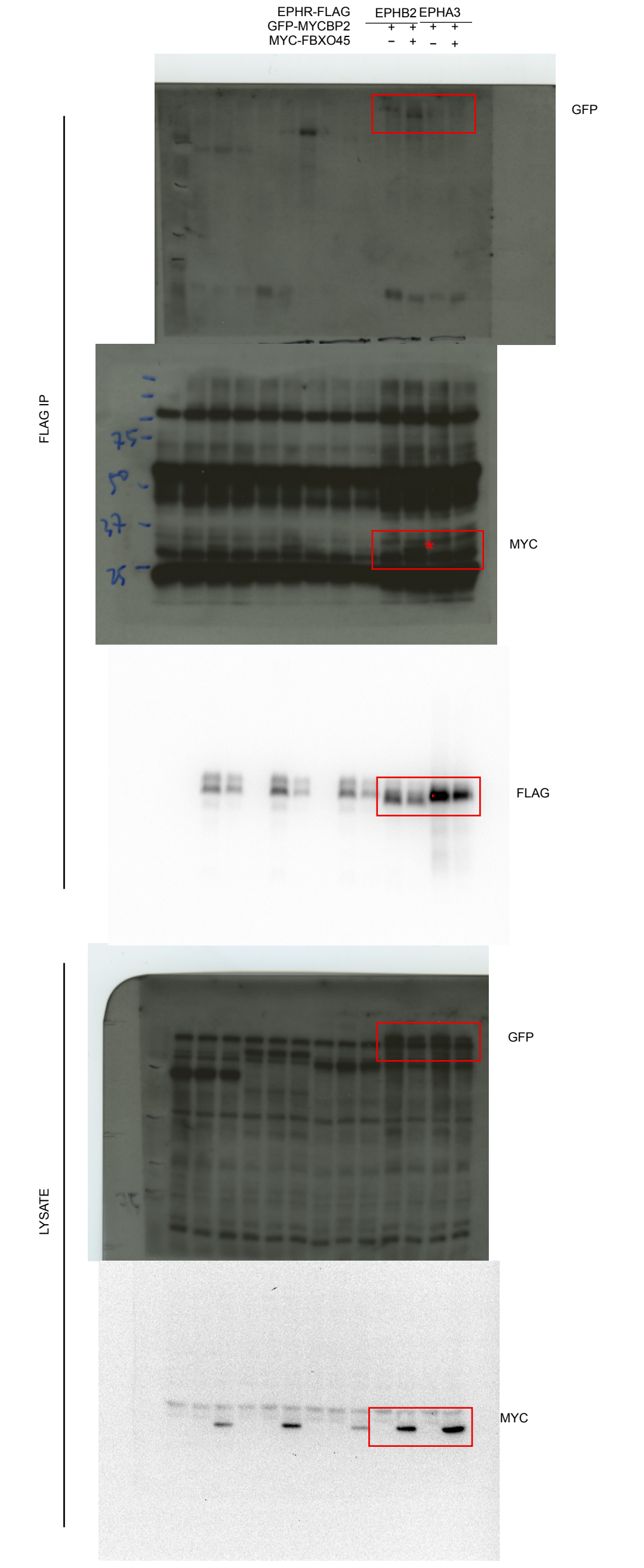

Figure 2E

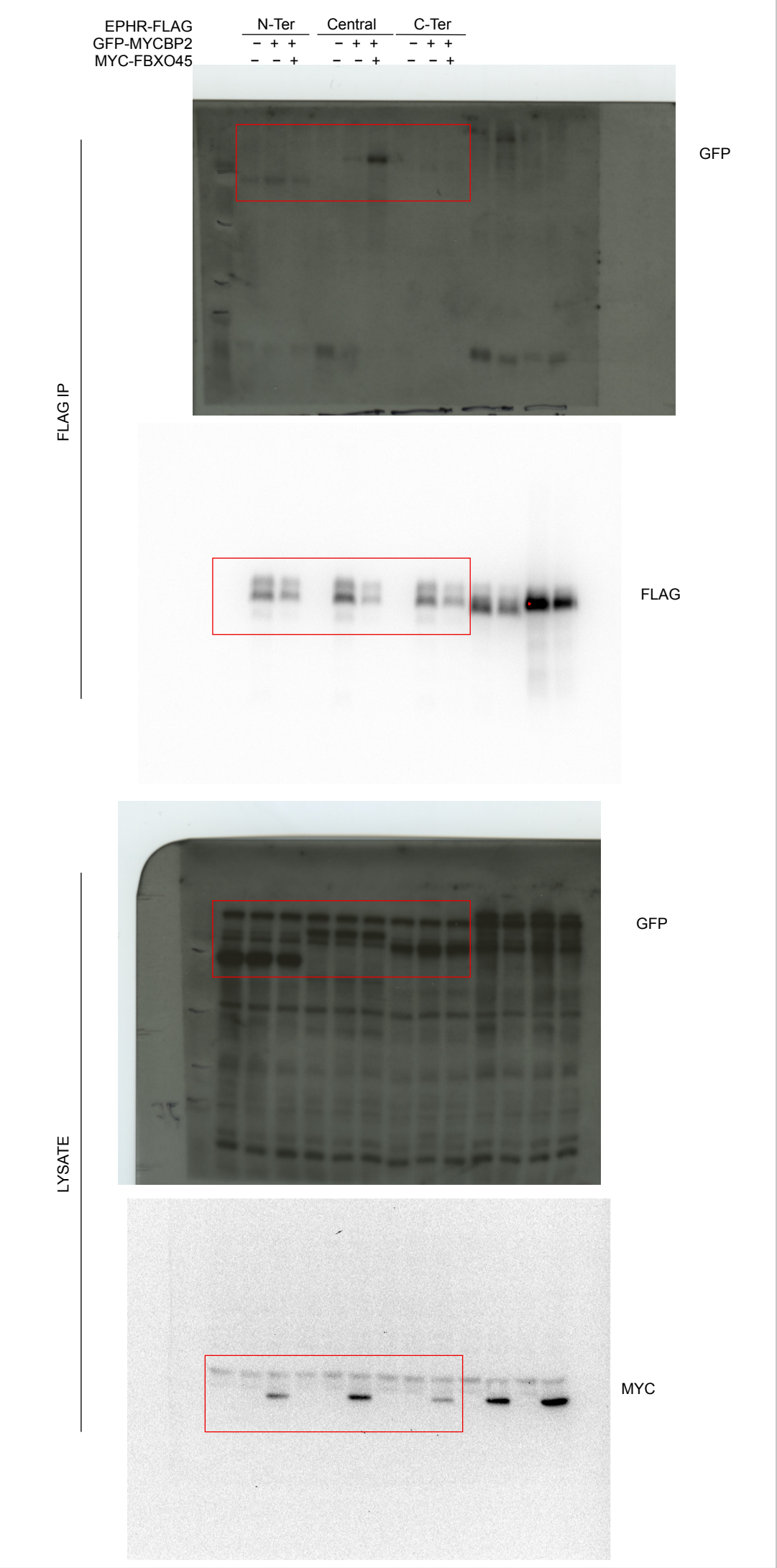

Supplement: Figure 2—source data 1. [file elife-89176-fig2-data1.zip › Figure 2 - source data 1/Figure 2-source data 1.pdf]

**Figure 2G**

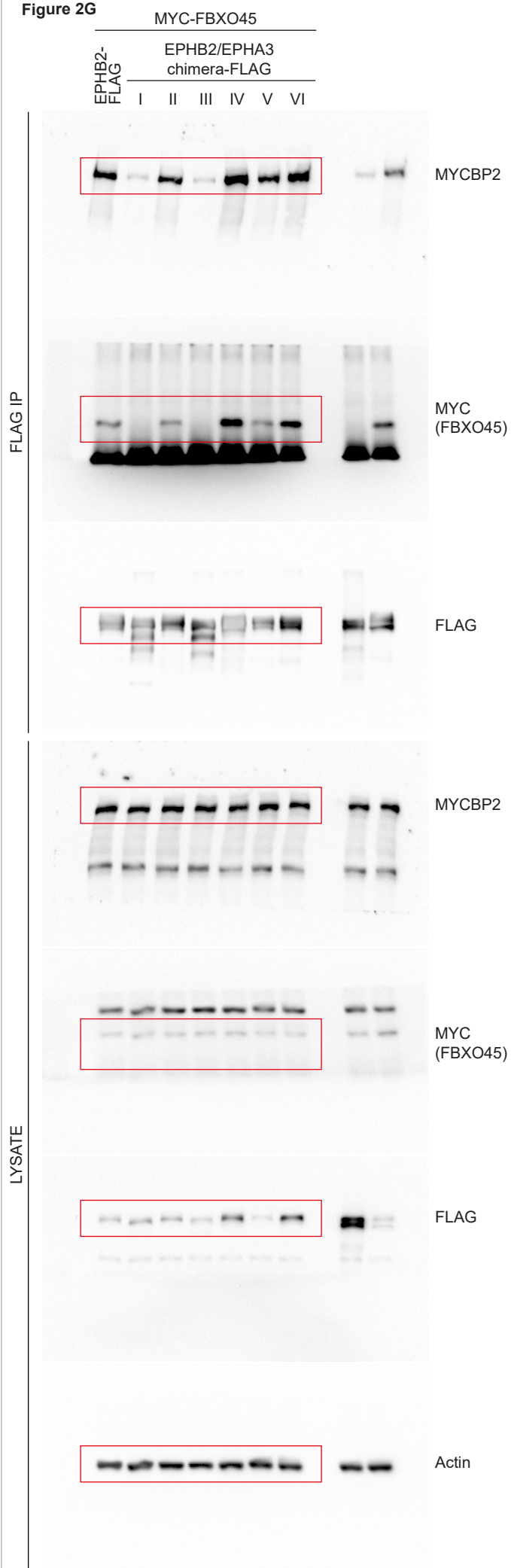

**Figure 2I**

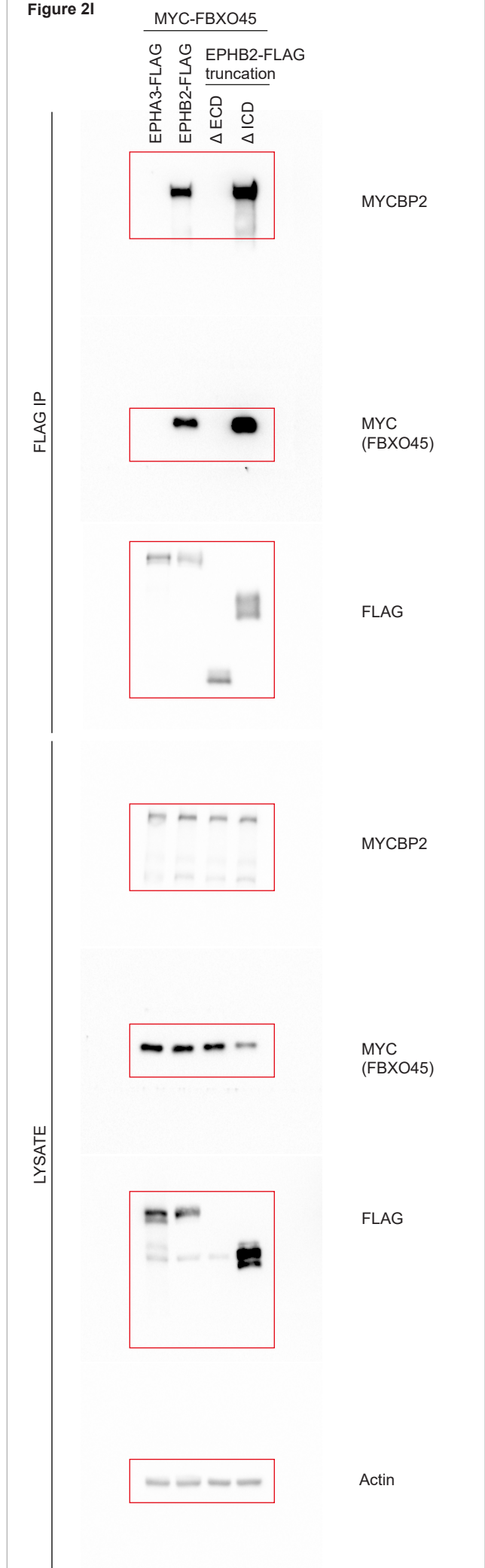

Supplement: Figure 2—source data 2. [file elife-89176-fig2-data2.zip › Figure 2 - source data 2/Figure 2 - source data 2.pdf]

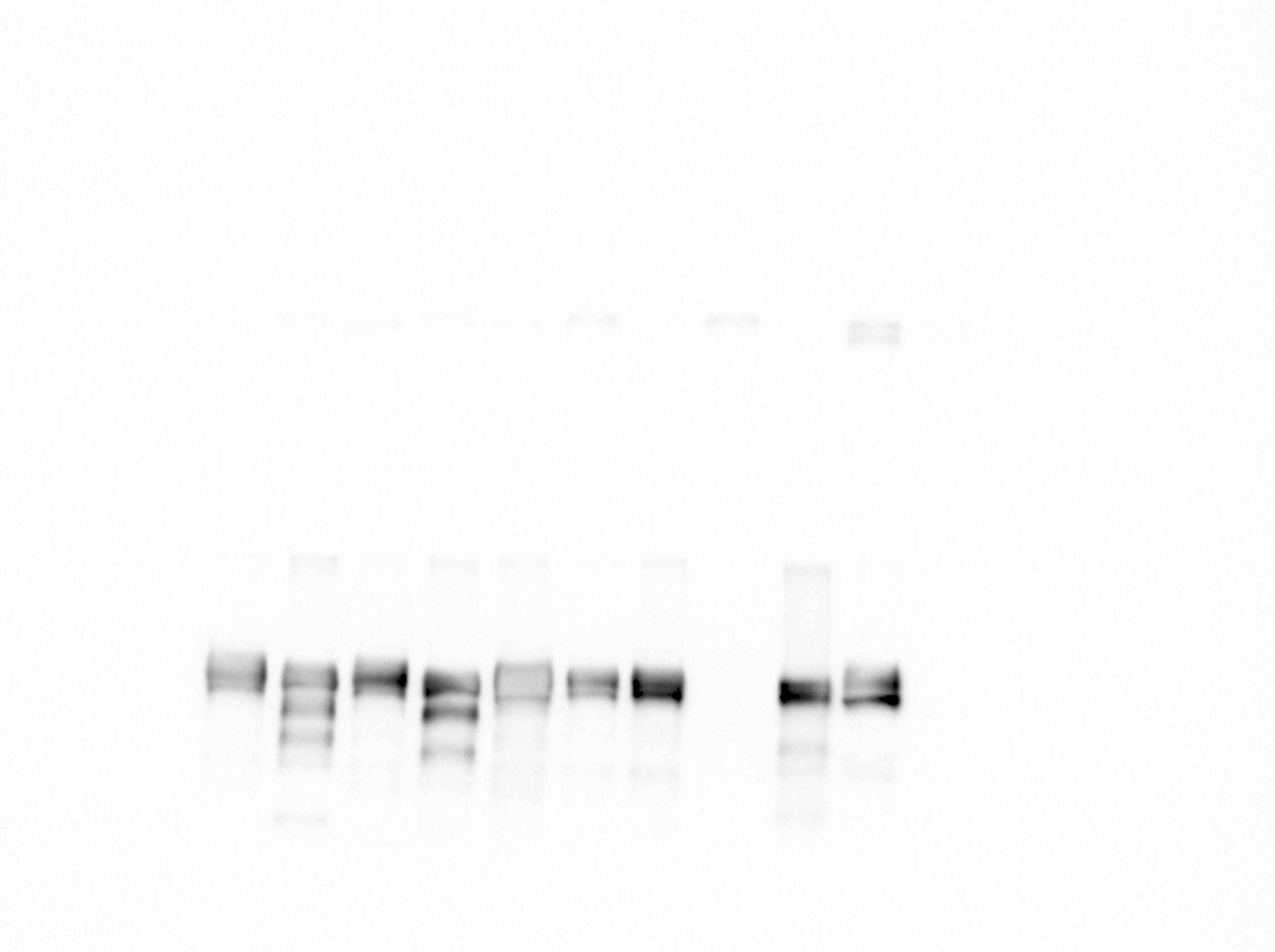

Supplement: Figure 2—source data 2. [file elife-89176-fig2-data2.zip › Figure 2 - source data 2/Figure 2G - IP - WB FLAG.tif]

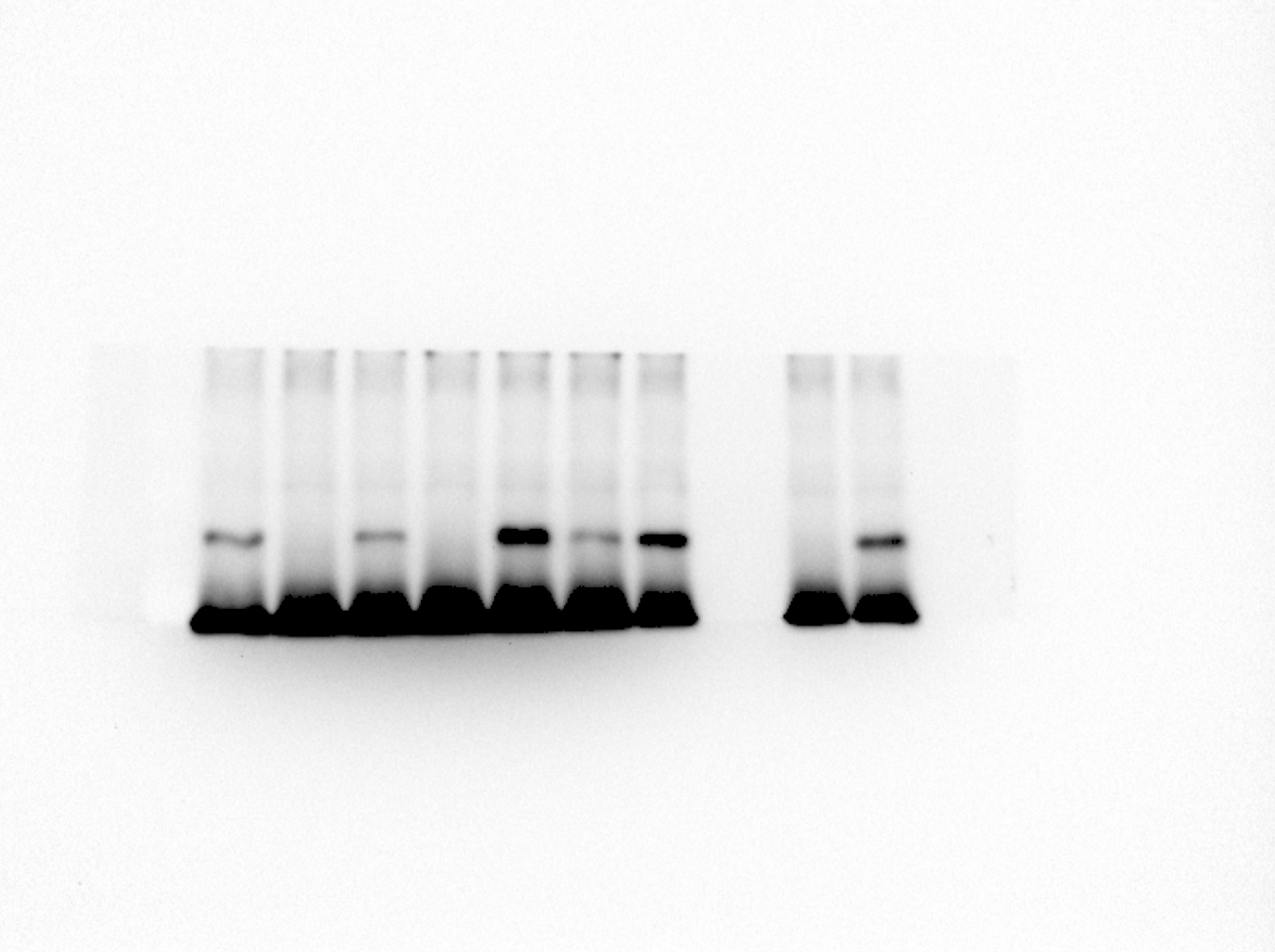

Supplement: Figure 2—source data 2. [file elife-89176-fig2-data2.zip › Figure 2 - source data 2/Figure 2G - IP - WB MYC.tif]

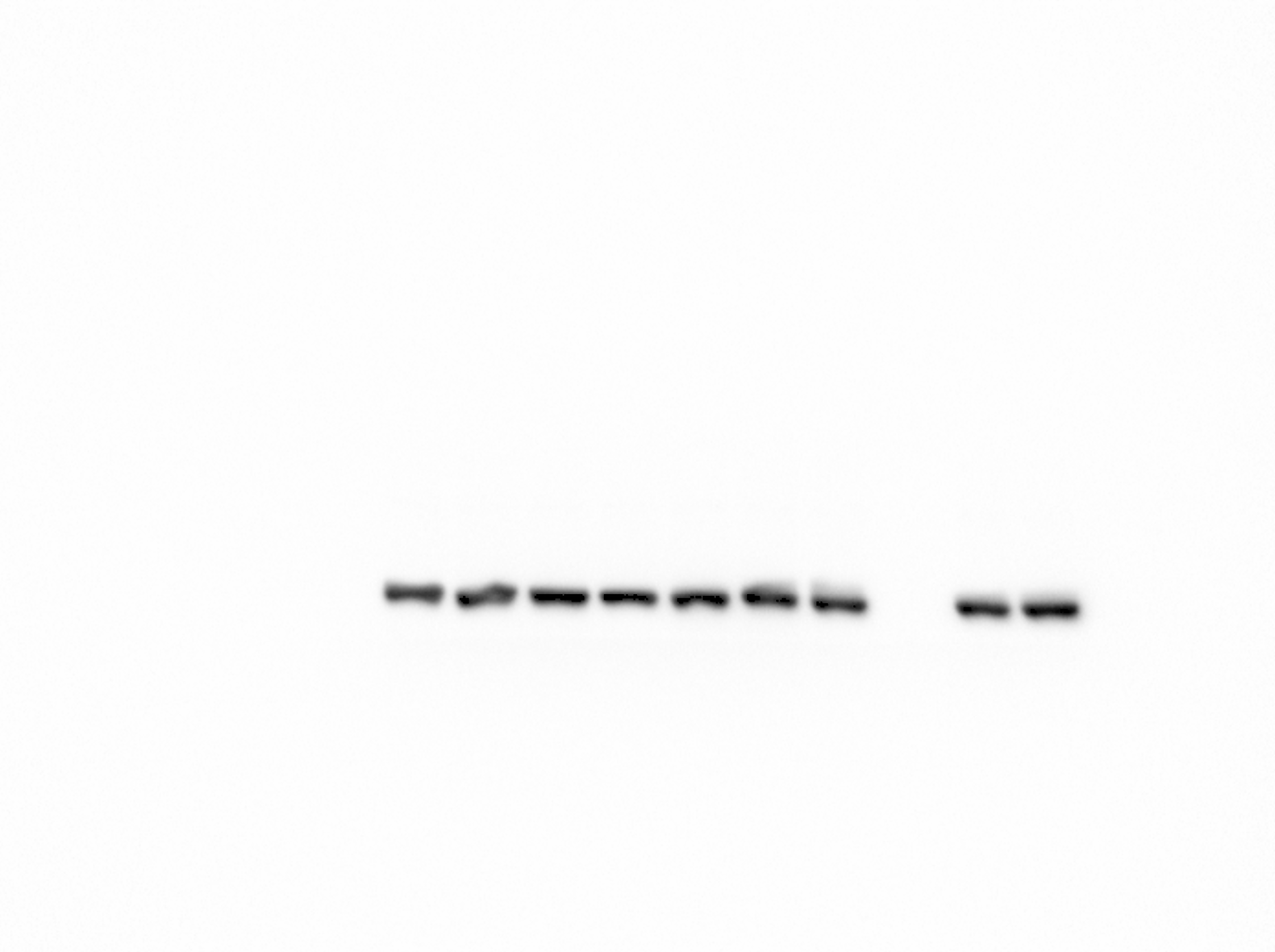

Supplement: Figure 2—source data 2. [file elife-89176-fig2-data2.zip › Figure 2 - source data 2/Figure 2G - Lysate - WB Actin.tif]

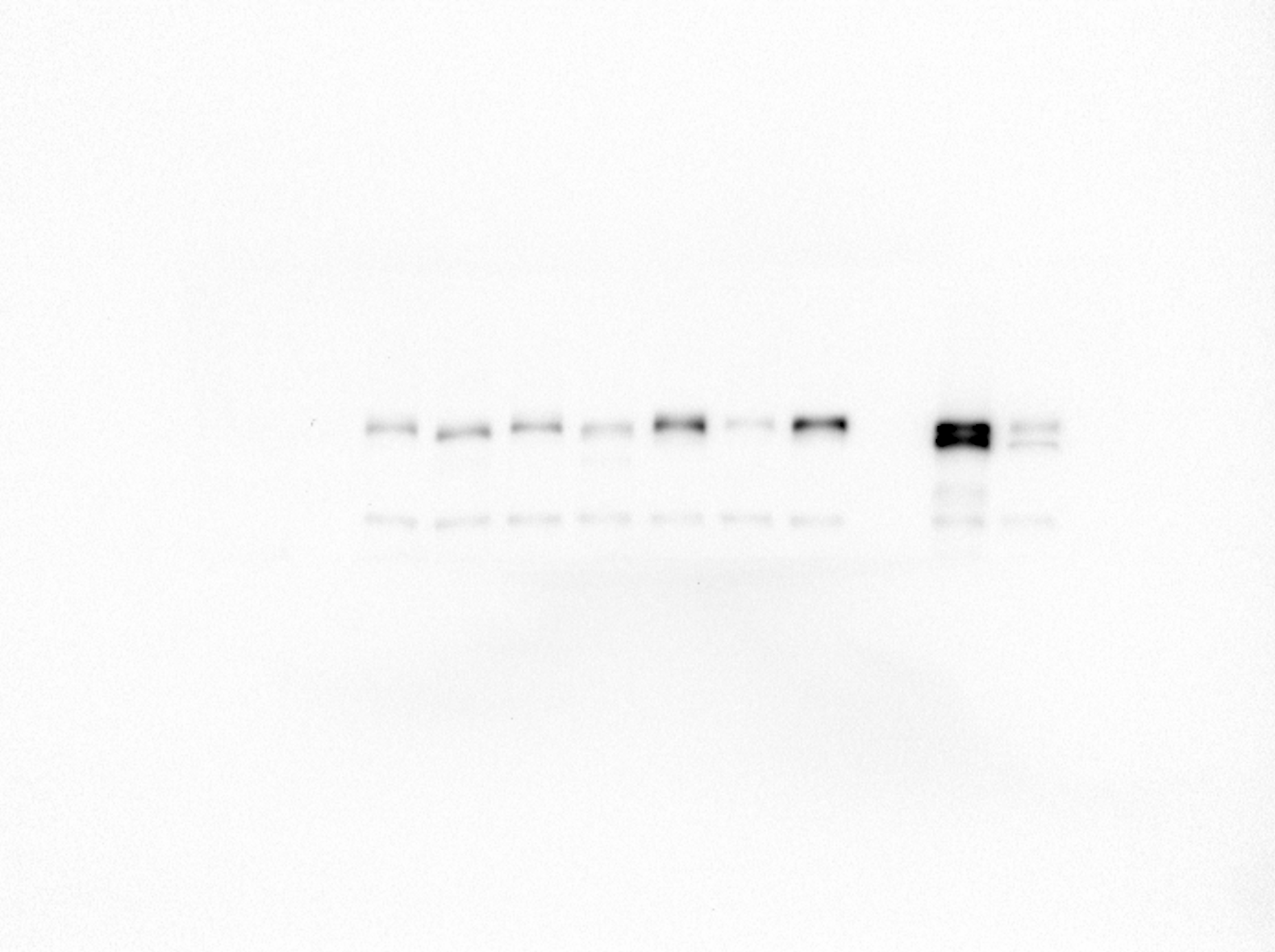

Supplement: Figure 2—source data 2. [file elife-89176-fig2-data2.zip › Figure 2 - source data 2/Figure 2G - Lysate - WB FLAG.tif]

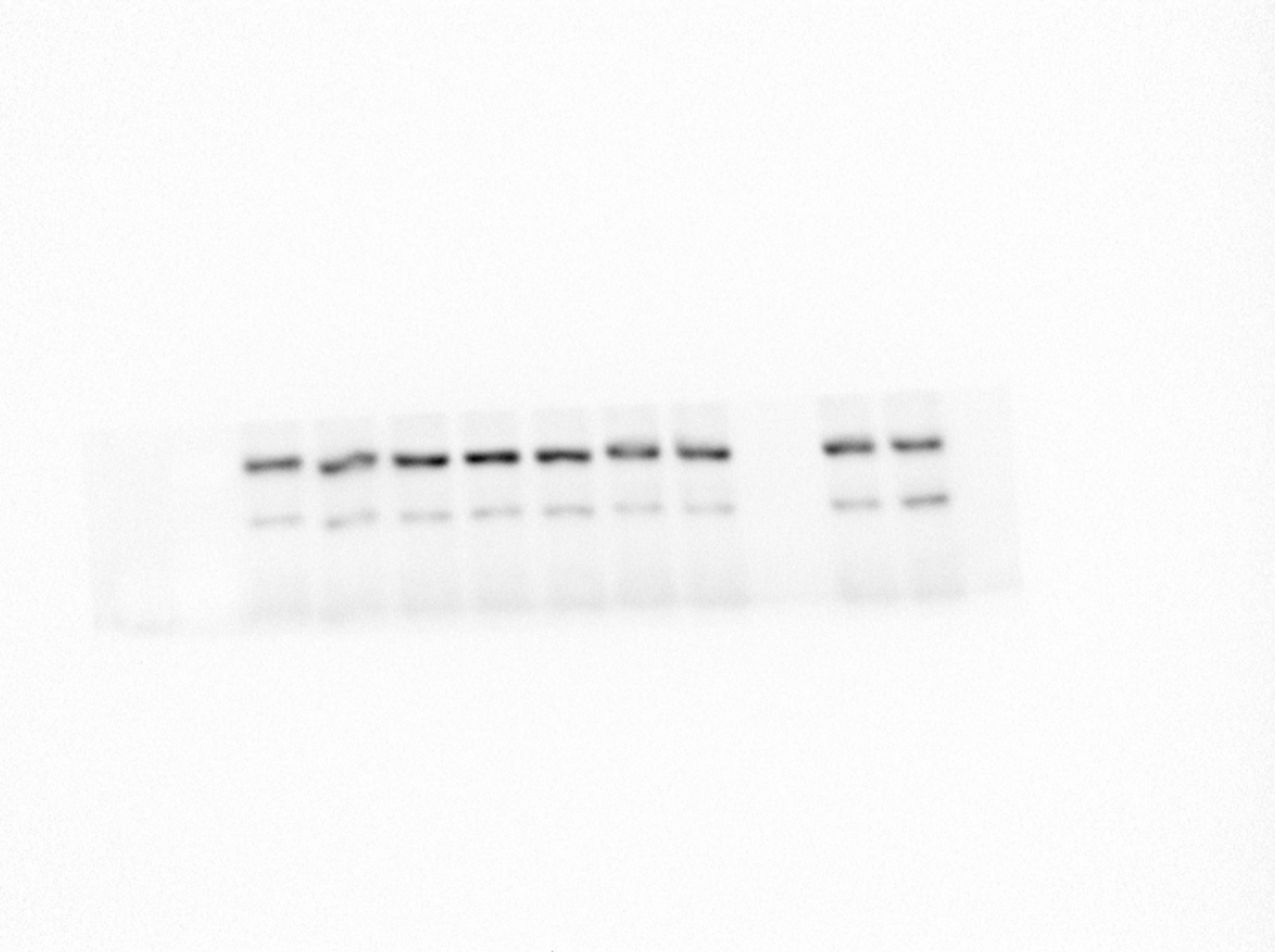

Supplement: Figure 2—source data 2. [file elife-89176-fig2-data2.zip › Figure 2 - source data 2/Figure 2G - Lysate - WB MYC.tif]

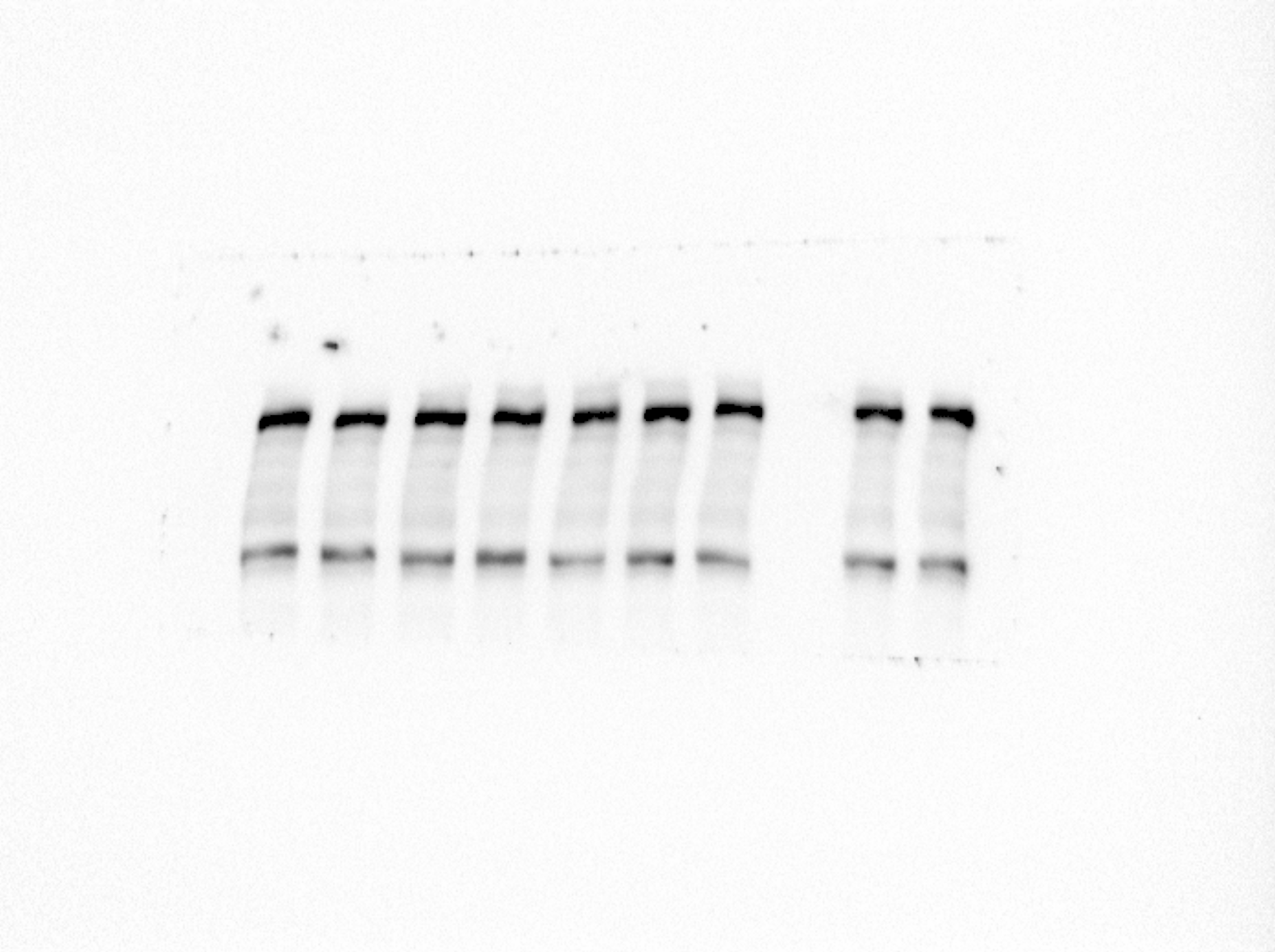

Supplement: Figure 2—source data 2. [file elife-89176-fig2-data2.zip › Figure 2 - source data 2/Figure 2G - Lysate - WB MYCBP2.tif]

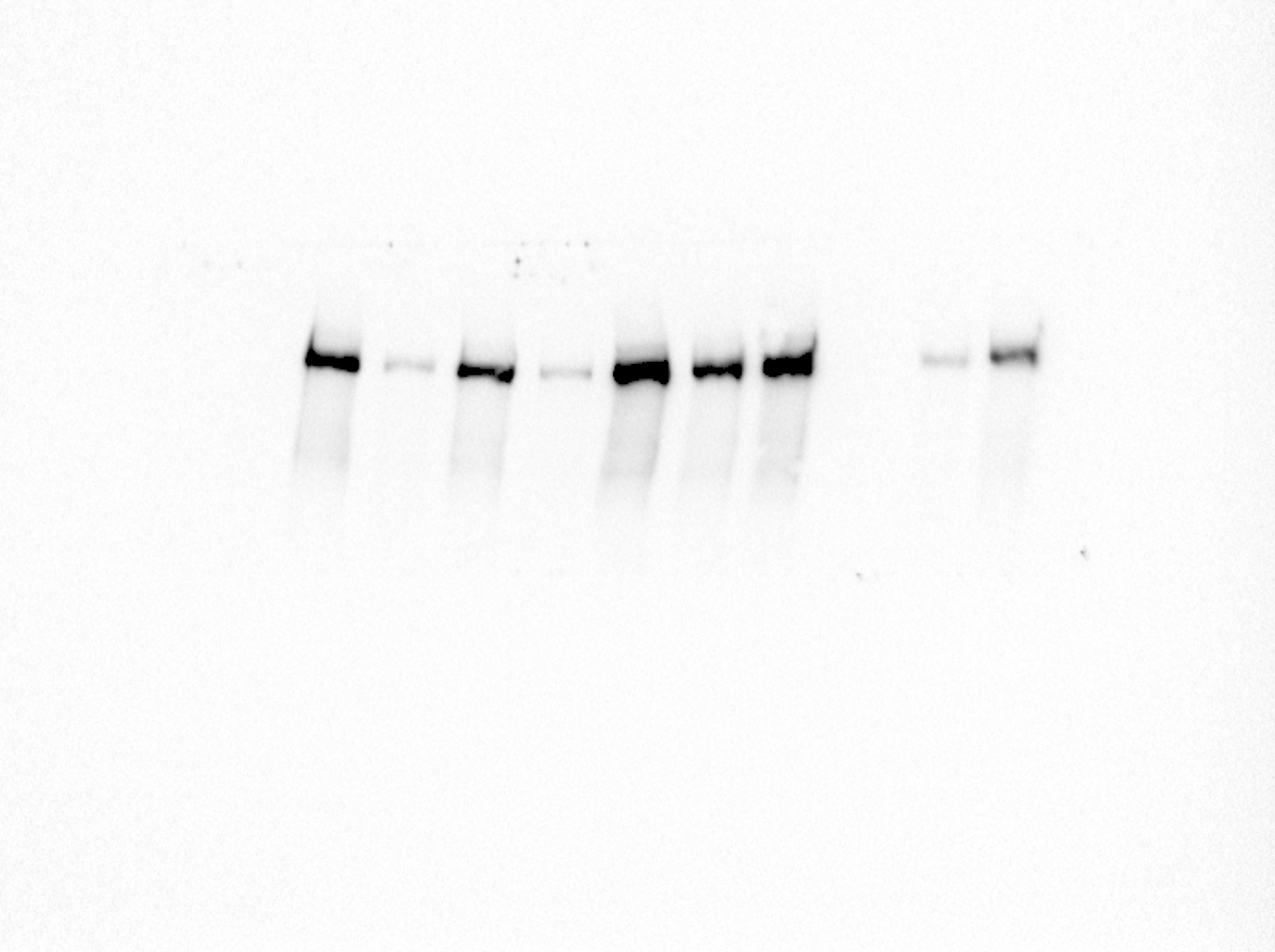

Supplement: Figure 2—source data 2. [file elife-89176-fig2-data2.zip › Figure 2 - source data 2/Figure 2G -IP - WB MYCBP2.tif]

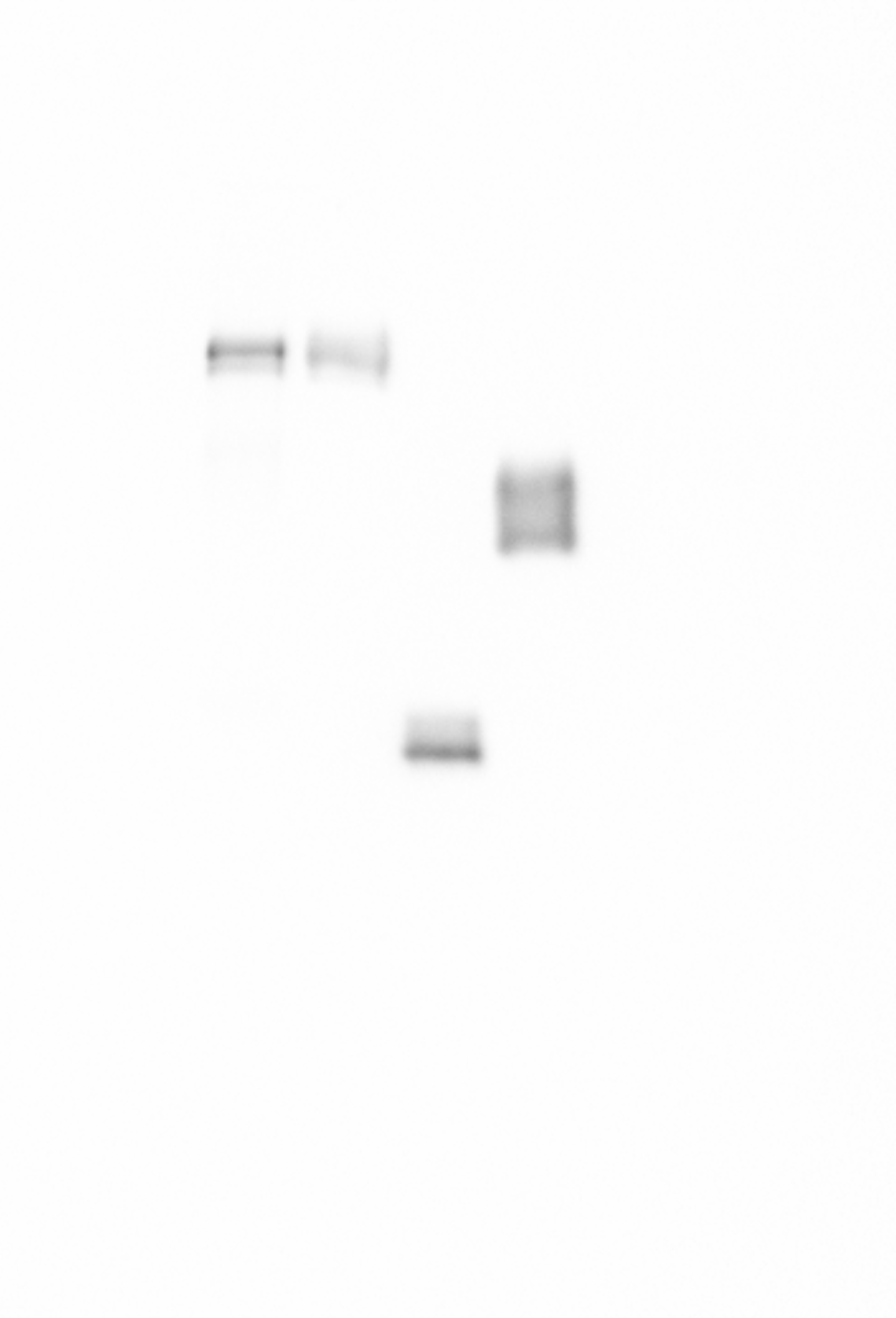

Supplement: Figure 2—source data 2. [file elife-89176-fig2-data2.zip › Figure 2 - source data 2/Figure 2I - IP - FLAG.tif]

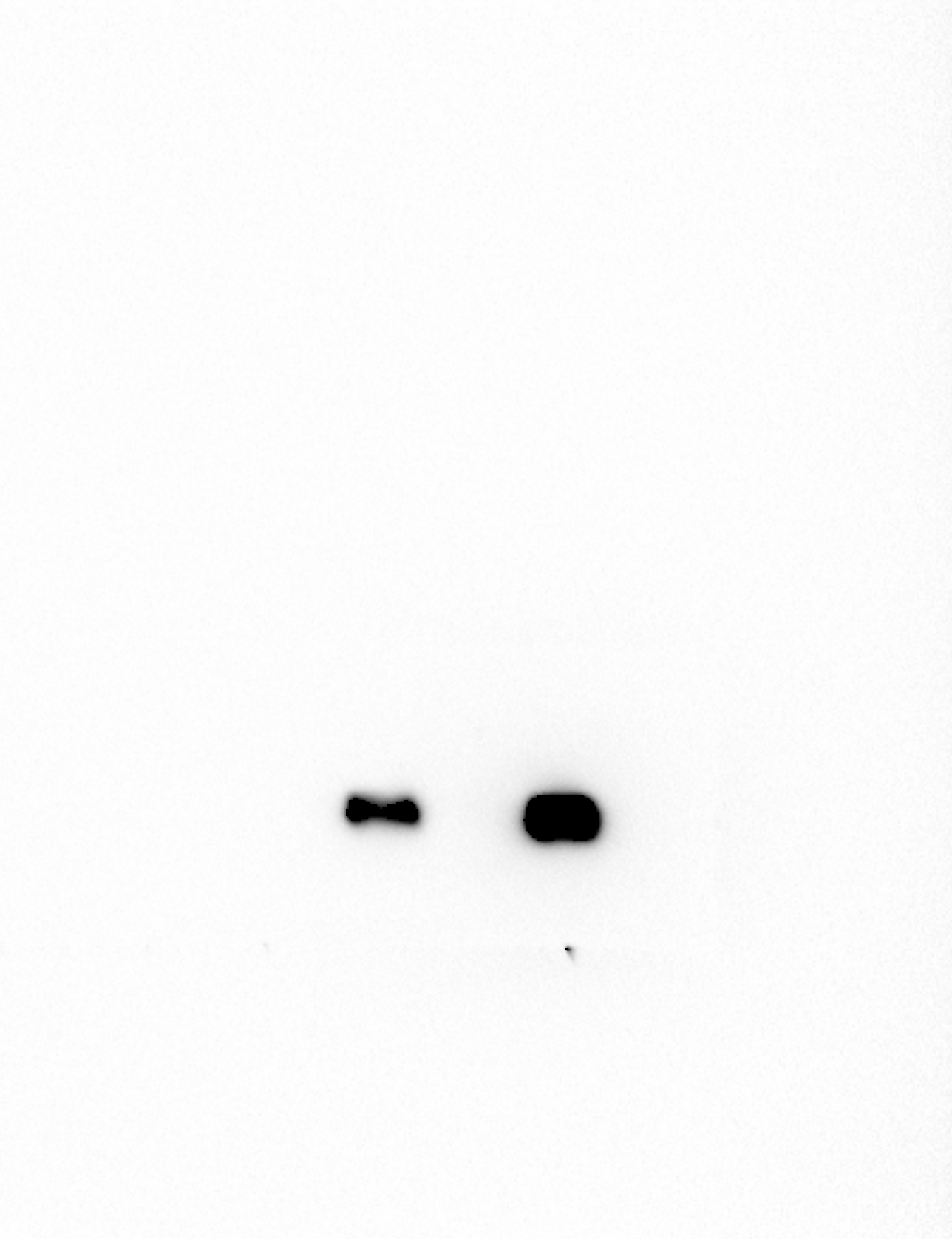

Supplement: Figure 2—source data 2. [file elife-89176-fig2-data2.zip › Figure 2 - source data 2/Figure 2I - IP - WB MYC.tif]

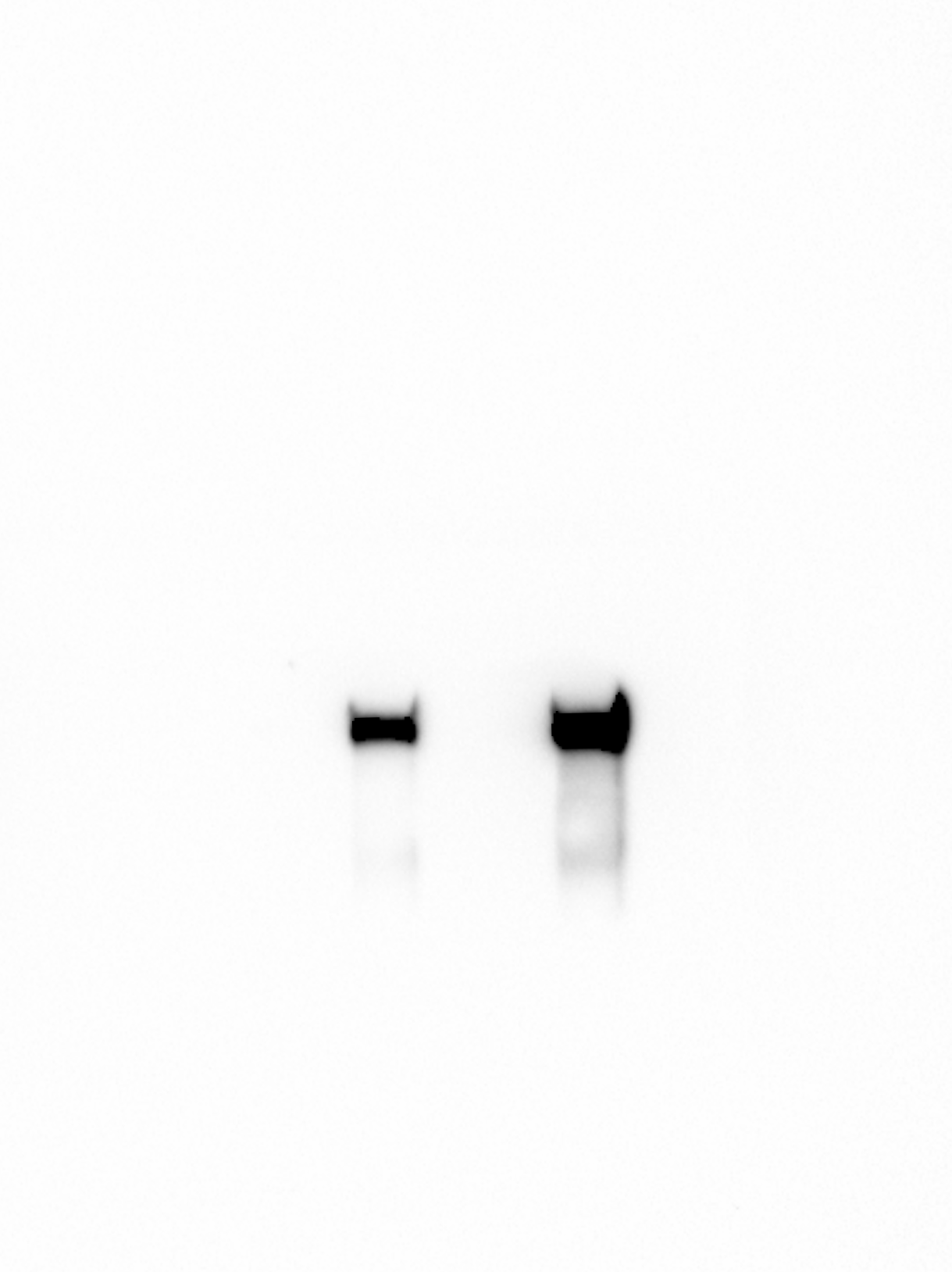

Supplement: Figure 2—source data 2. [file elife-89176-fig2-data2.zip › Figure 2 - source data 2/Figure 2I - IP - WB MYCBP2.tif]

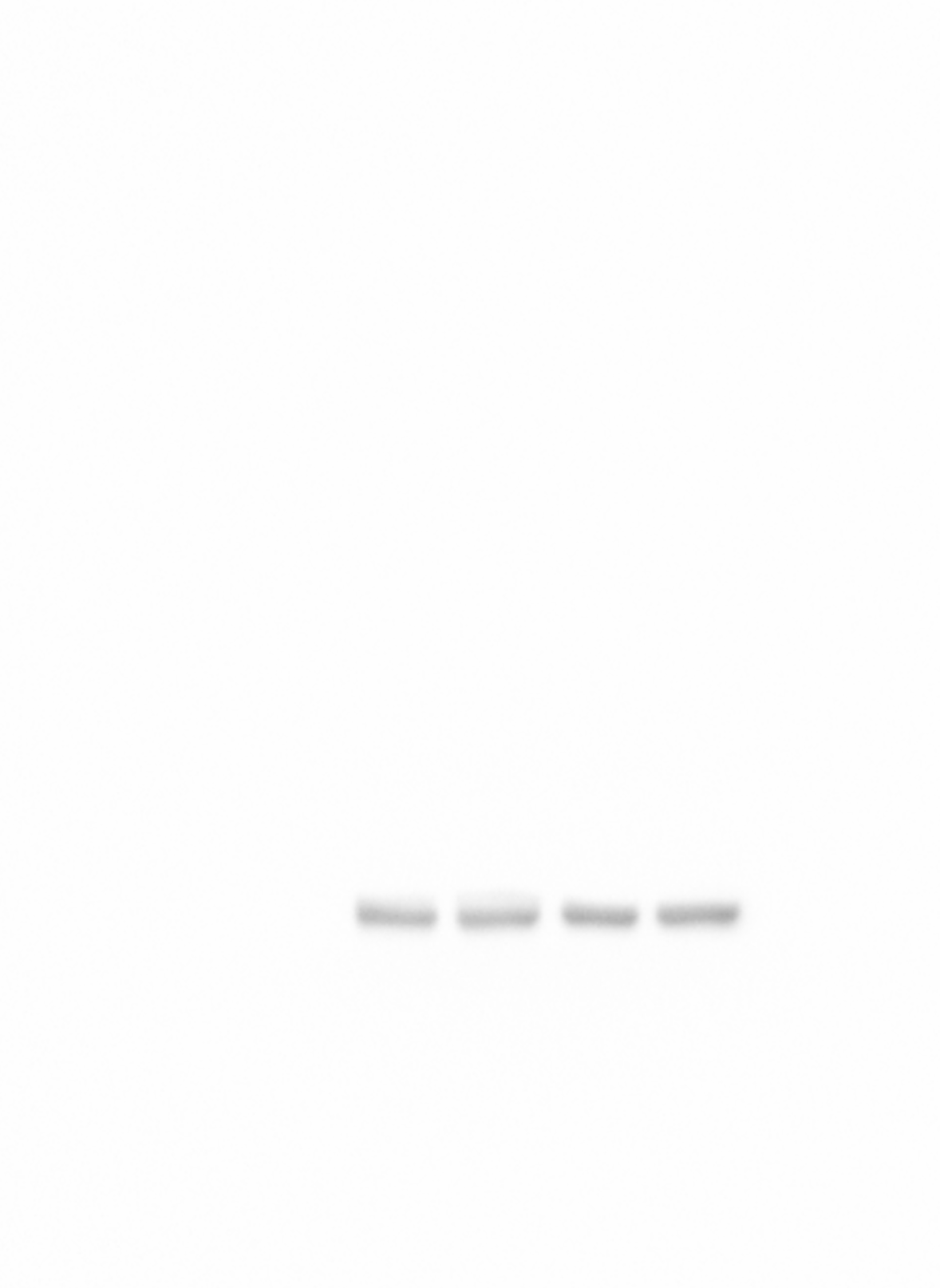

Supplement: Figure 2—source data 2. [file elife-89176-fig2-data2.zip › Figure 2 - source data 2/Figure 2I - Lysate - WB Actin.tif]

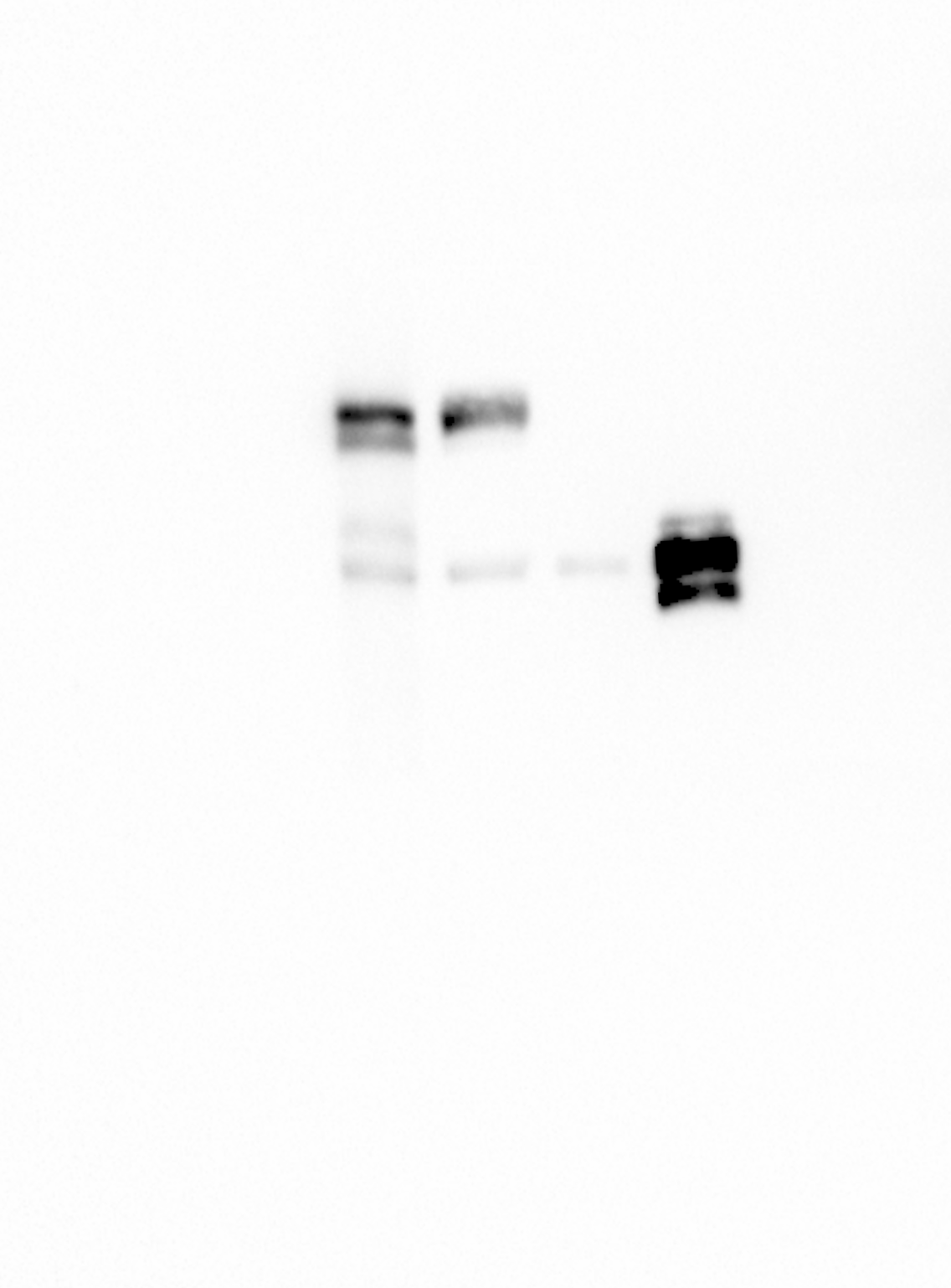

Supplement: Figure 2—source data 2. [file elife-89176-fig2-data2.zip › Figure 2 - source data 2/Figure 2I - Lysate - WB FLAG.tif]

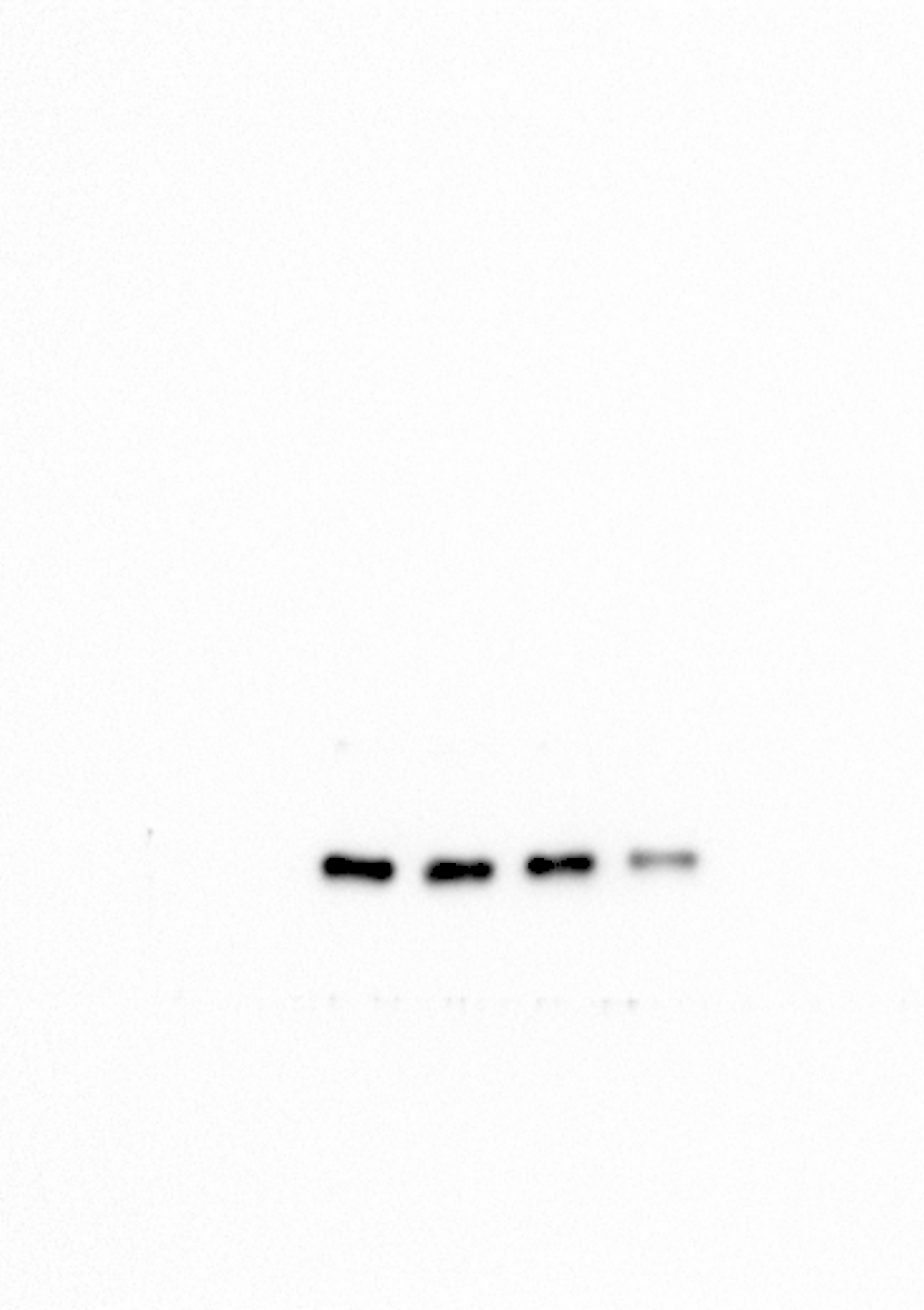

Supplement: Figure 2—source data 2. [file elife-89176-fig2-data2.zip › Figure 2 - source data 2/Figure 2I - Lysate - WB MYC.tif]

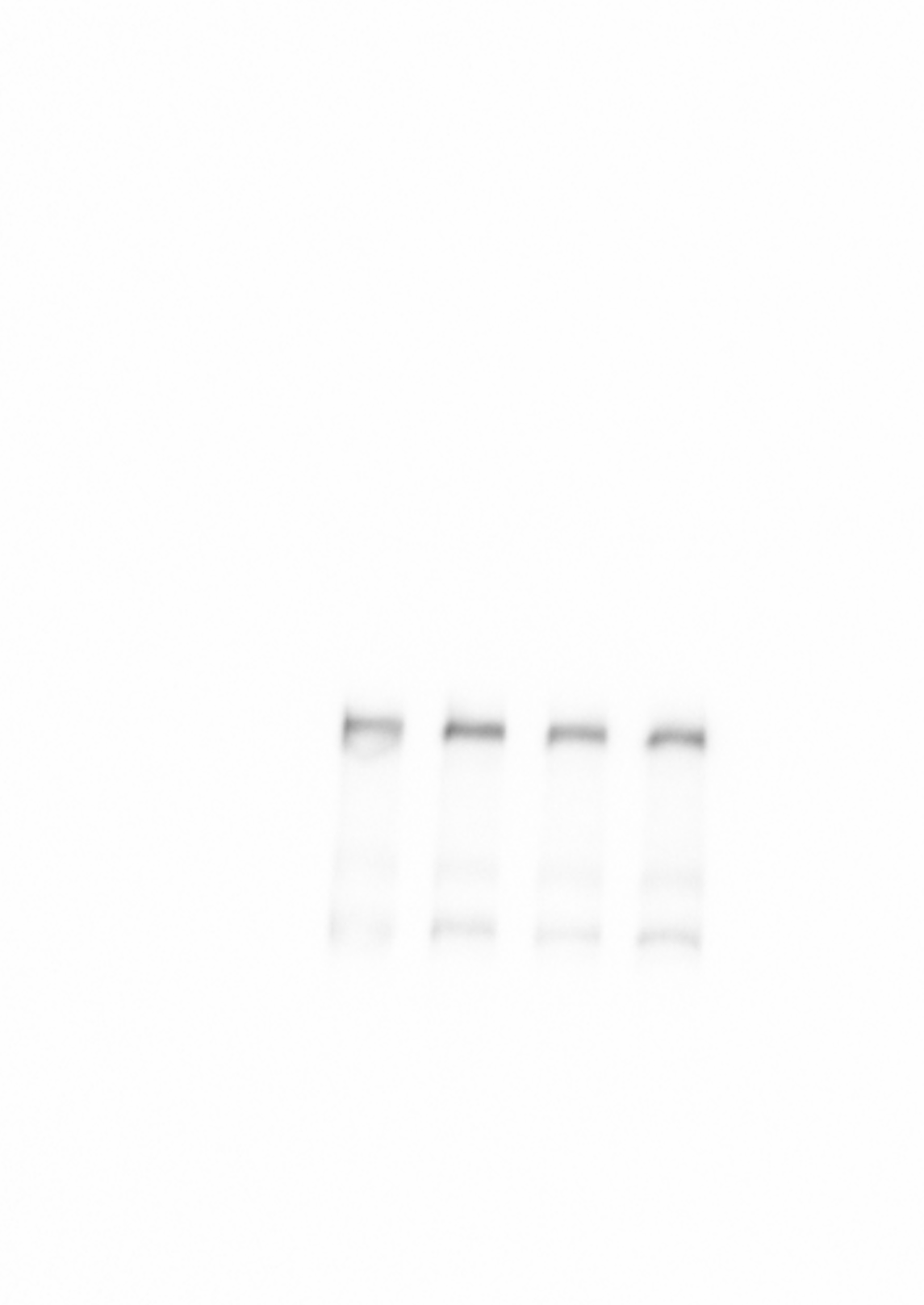

Supplement: Figure 2—source data 2. [file elife-89176-fig2-data2.zip › Figure 2 - source data 2/Figure 2I - Lysate - WB MYCBP2.tif]

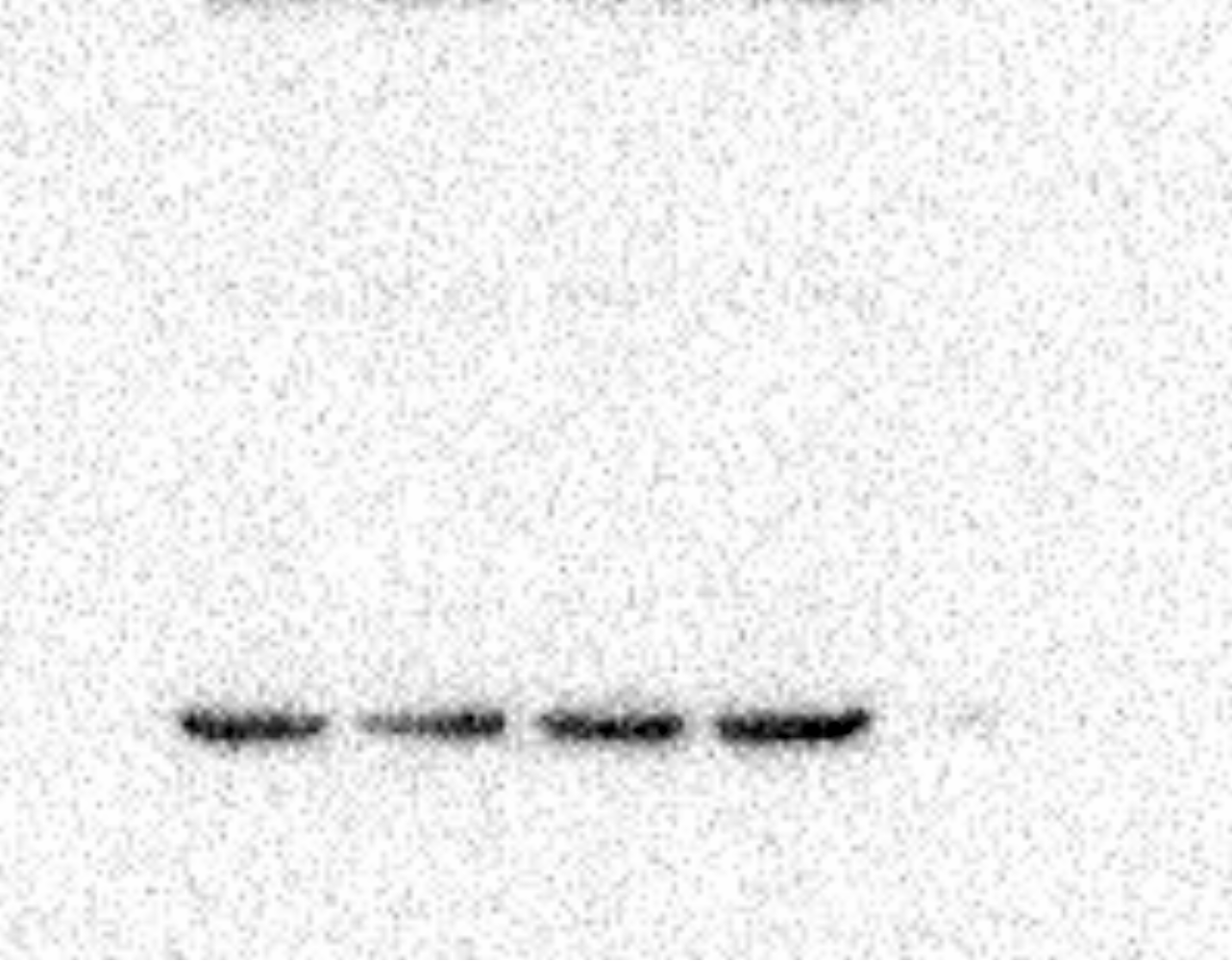

Supplement: Figure 3—source data 1. [file elife-89176-fig3-data1.zip › Figure 3 - source data 1/Figure 3B - Actin.tif]

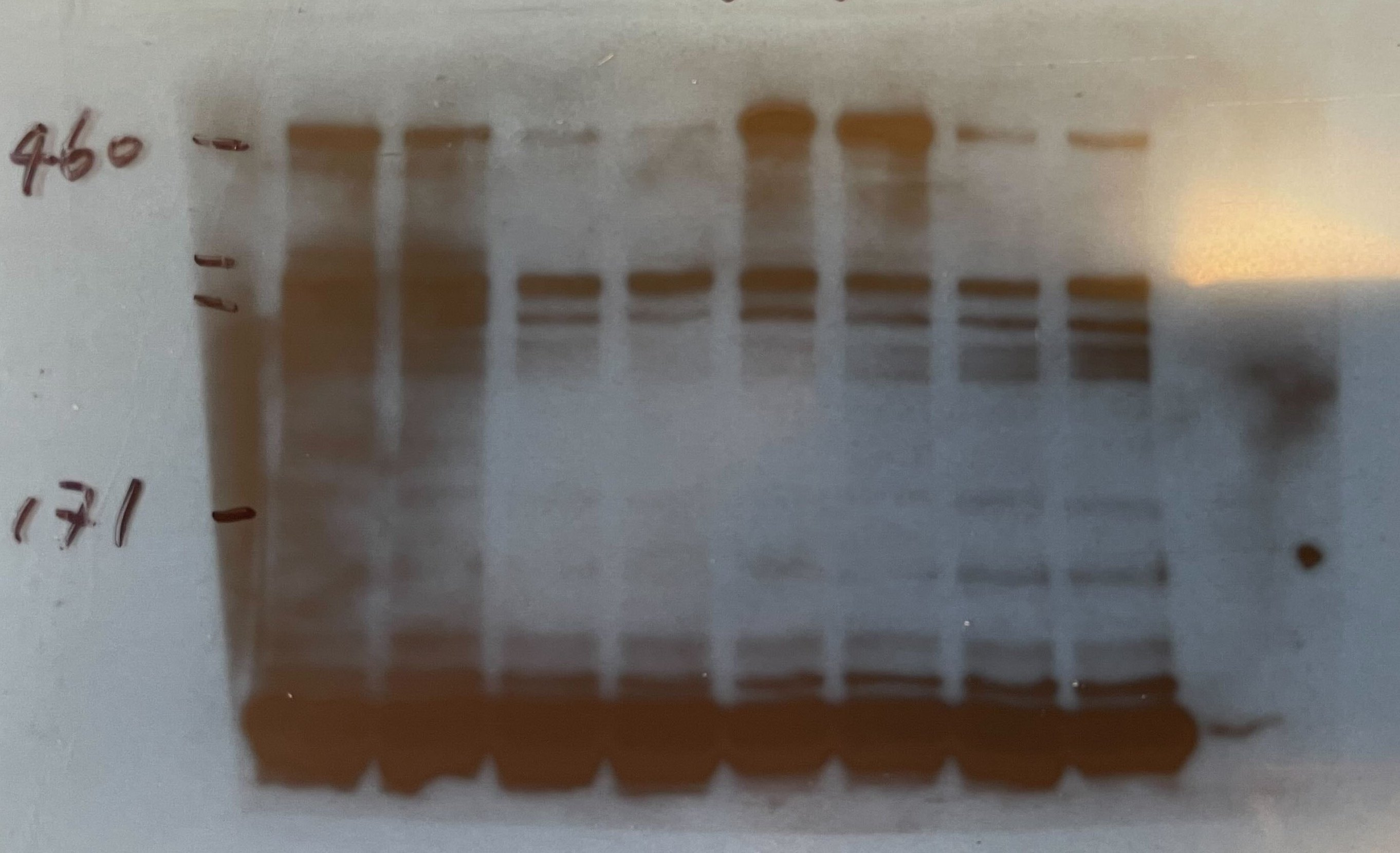

Supplement: Figure 3—source data 1. [file elife-89176-fig3-data1.zip › Figure 3 - source data 1/Figure 3B - MYCBP2.tif]

Figure 3B

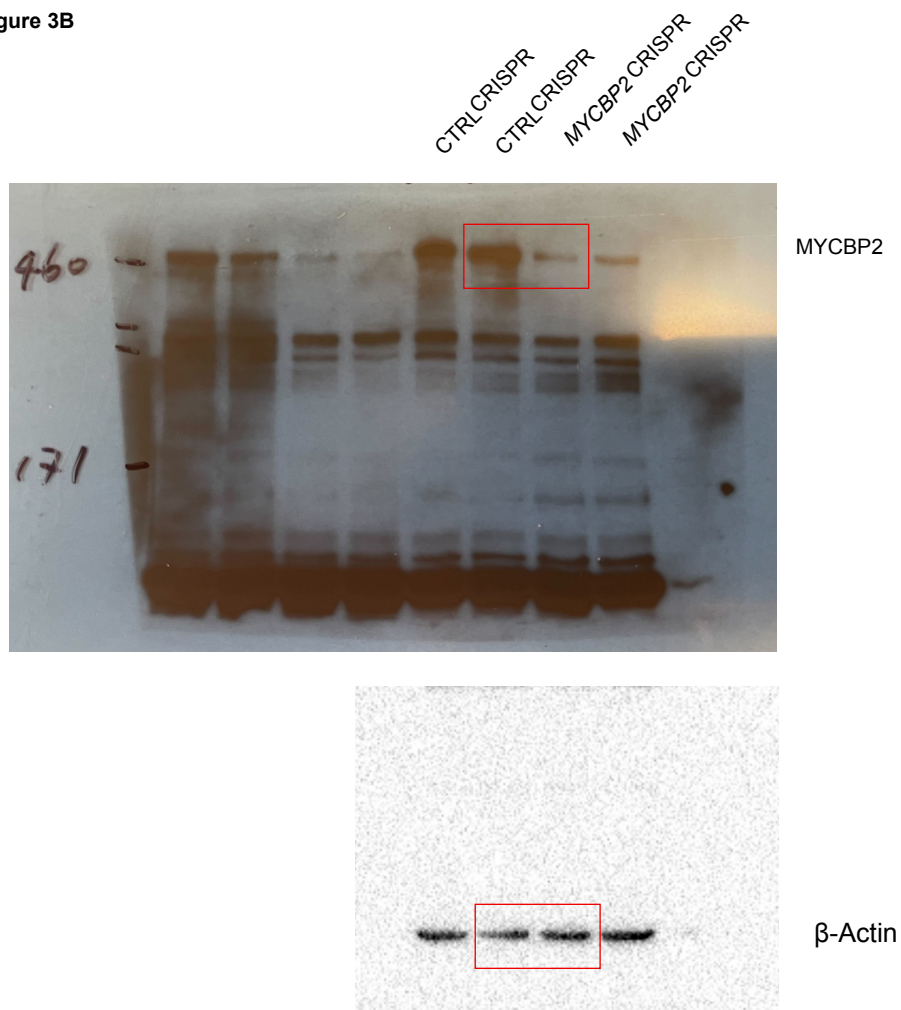

Supplement: Figure 3—source data 1. [file elife-89176-fig3-data1.zip › Figure 3 - source data 1/Figure 3-source data 1.pdf]

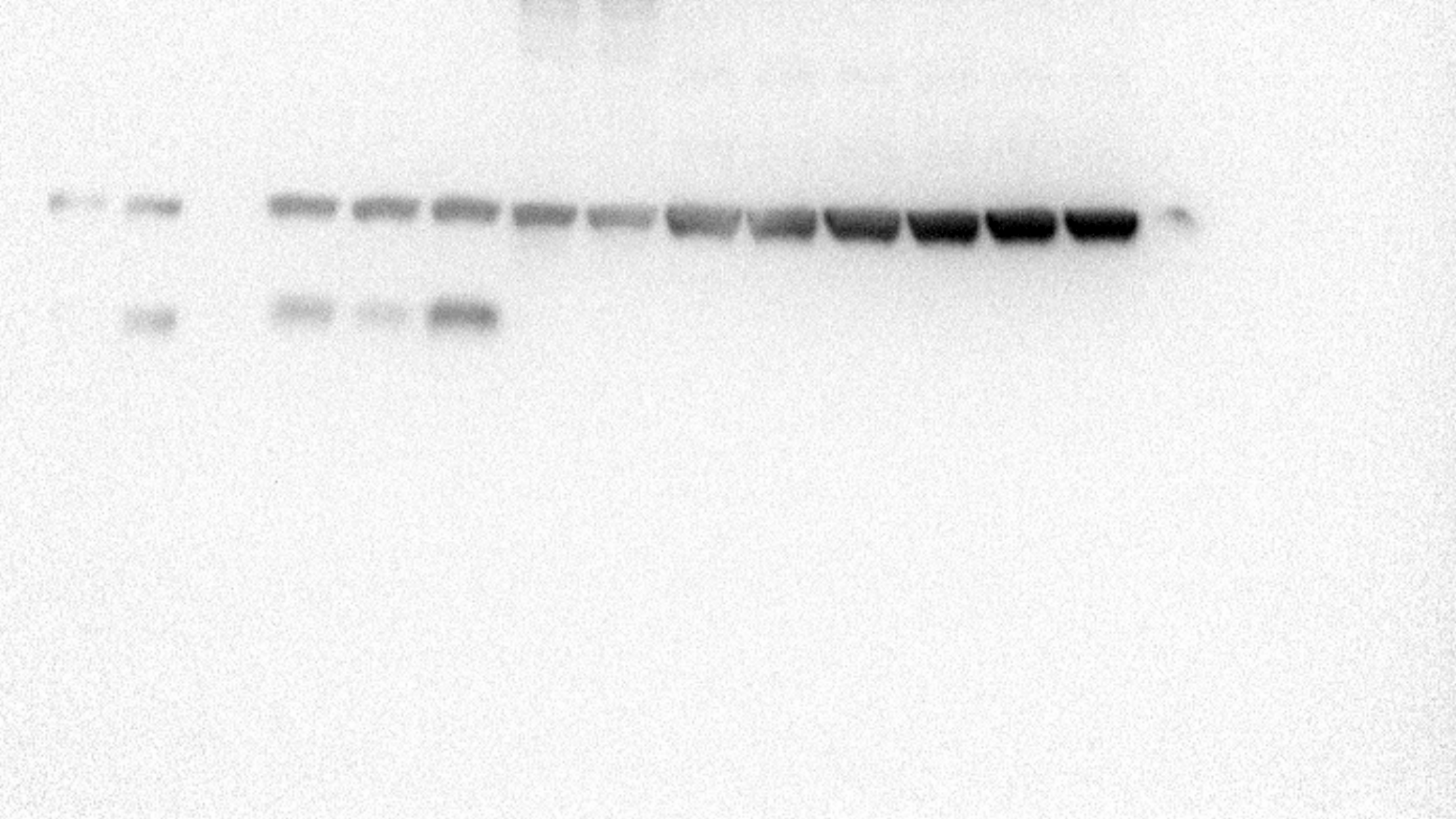

Supplement: Figure 4—source data 1. [file elife-89176-fig4-data1.zip › Figure 4 - source data 1/Figure 4A - Actin.tif]

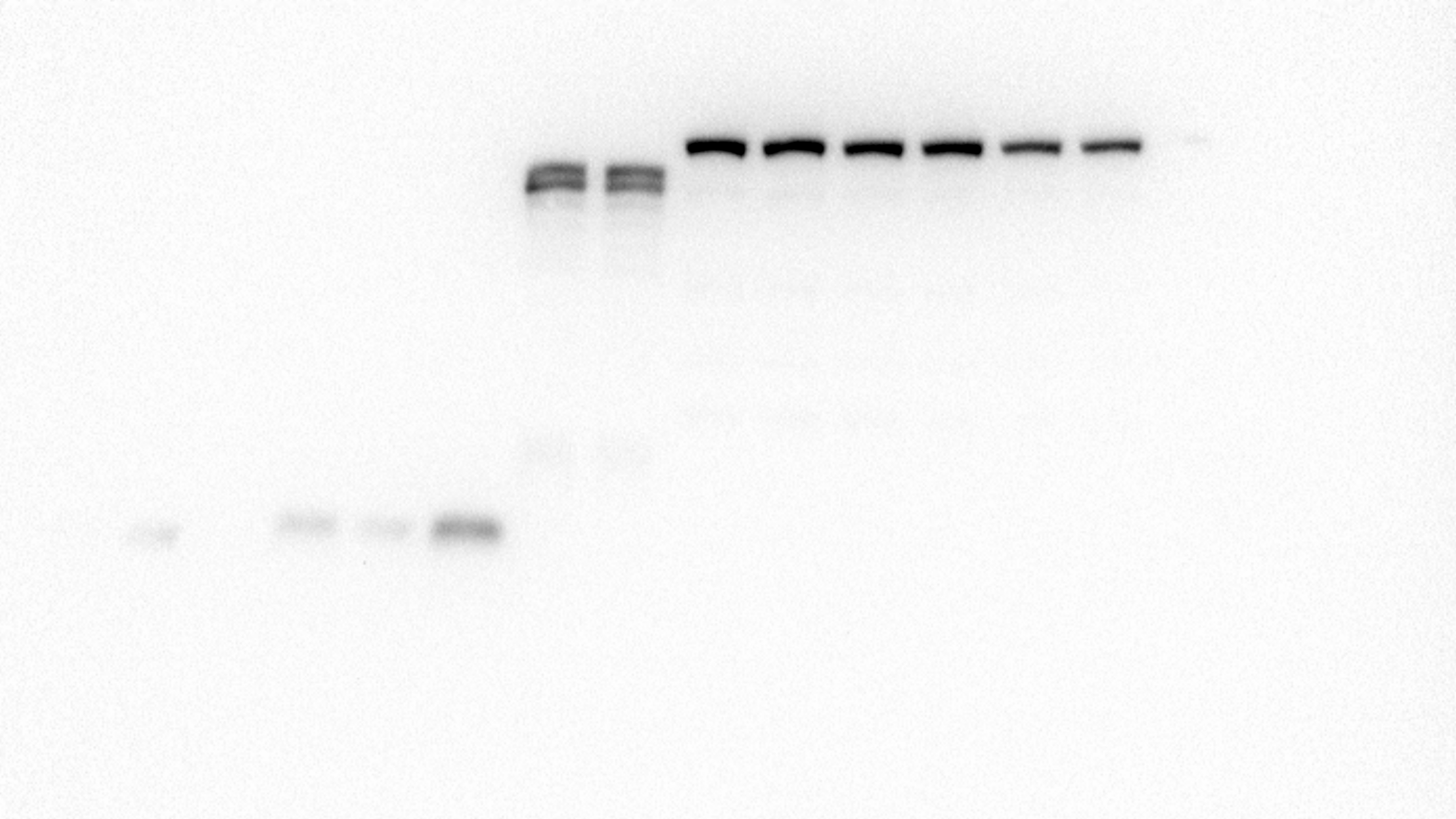

Supplement: Figure 4—source data 1. [file elife-89176-fig4-data1.zip › Figure 4 - source data 1/Figure 4A - FLAG(EPHB2).tif]

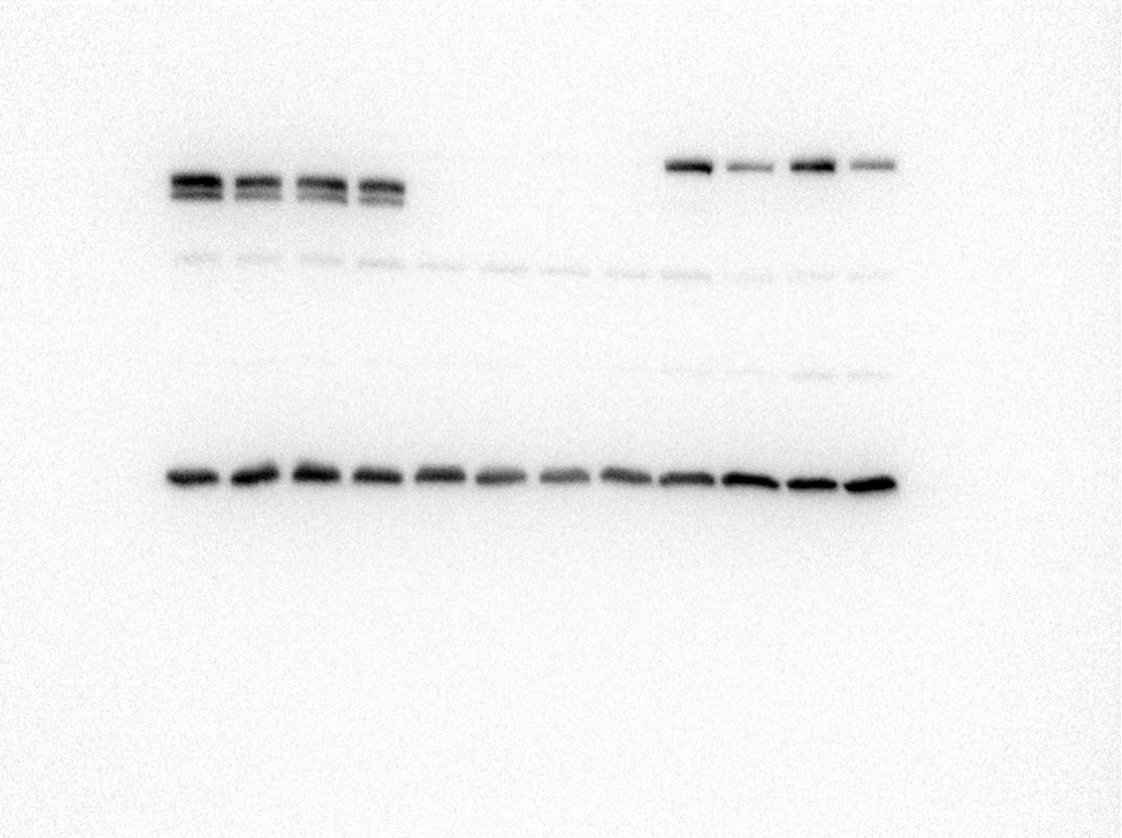

Supplement: Figure 4—source data 1. [file elife-89176-fig4-data1.zip › Figure 4 - source data 1/Figure 4B - FLAG(EPHB2) and Actin.tif]

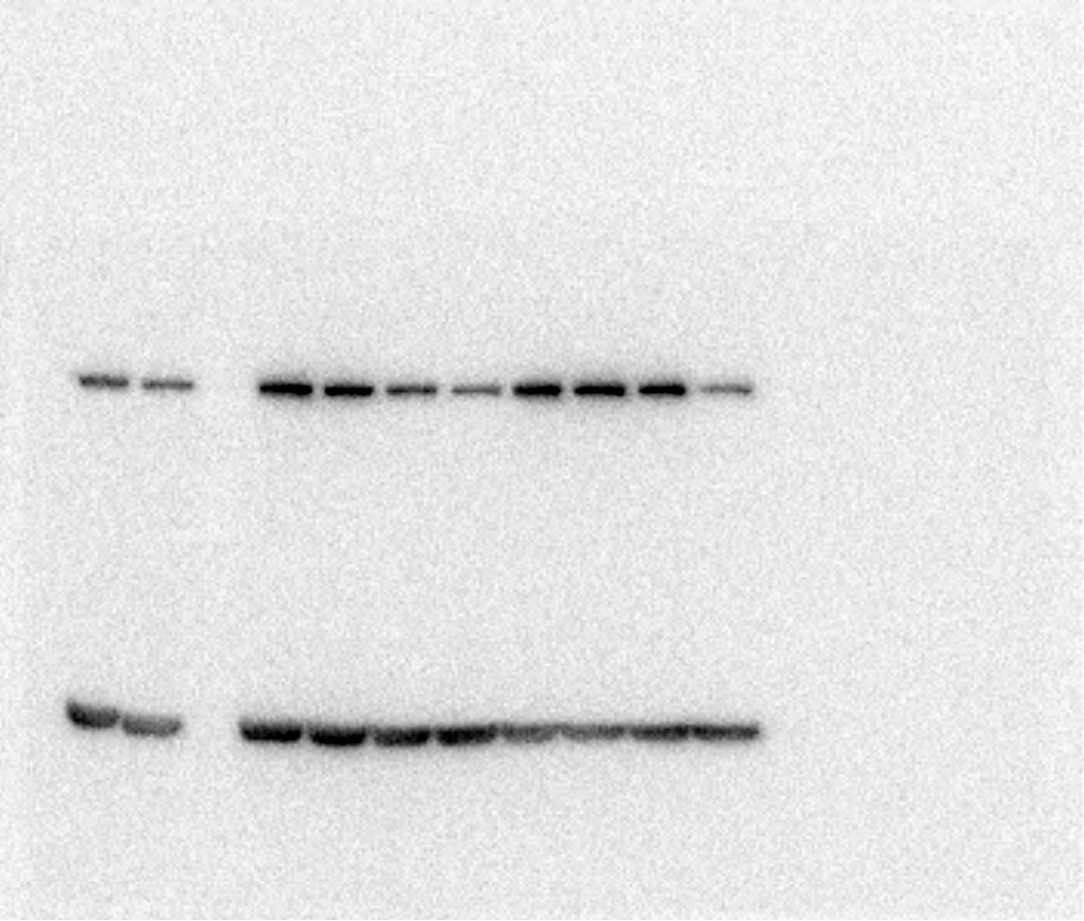

Supplement: Figure 4—source data 1. [file elife-89176-fig4-data1.zip › Figure 4 - source data 1/Figure 4D - FLAG(EPHB2) and Actin.tif]

Figure 4A

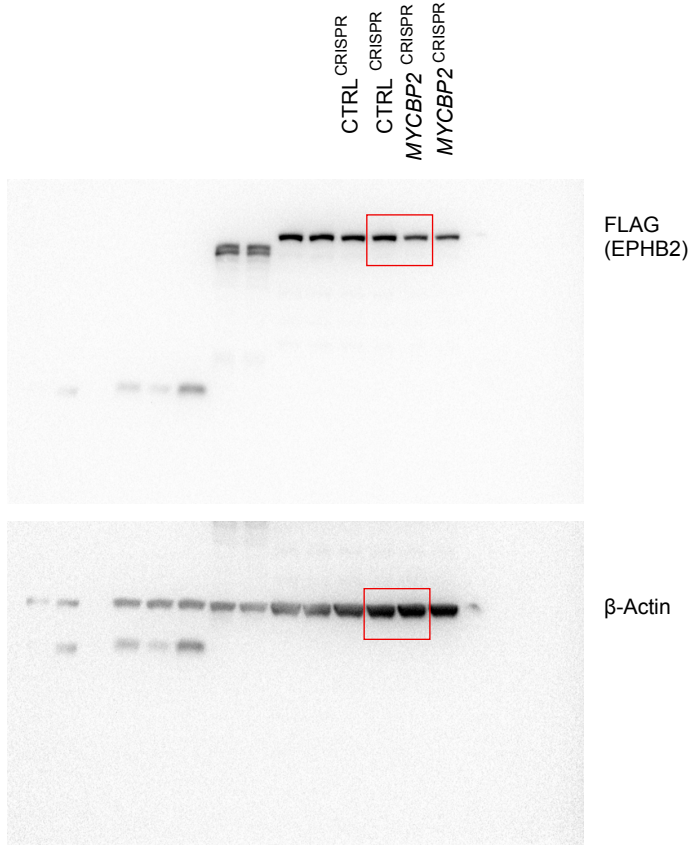

Figure 4B

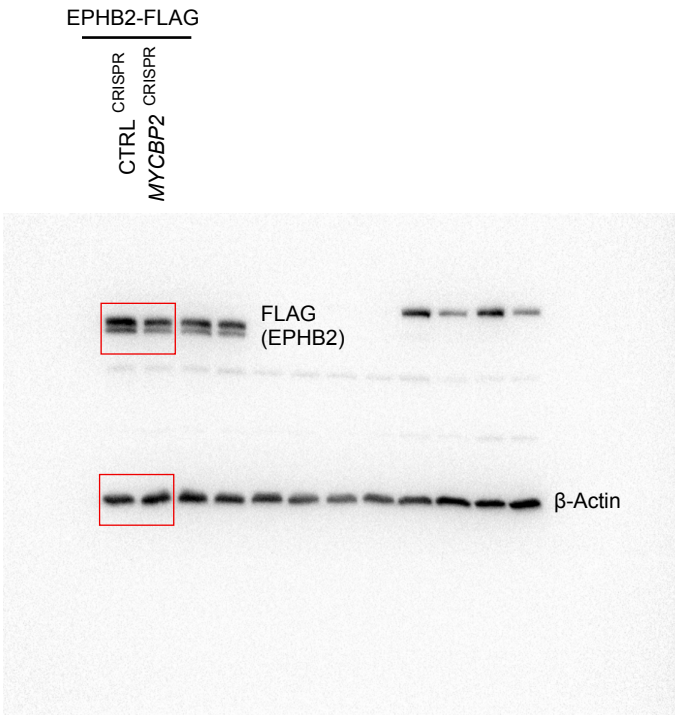

Figure 4D

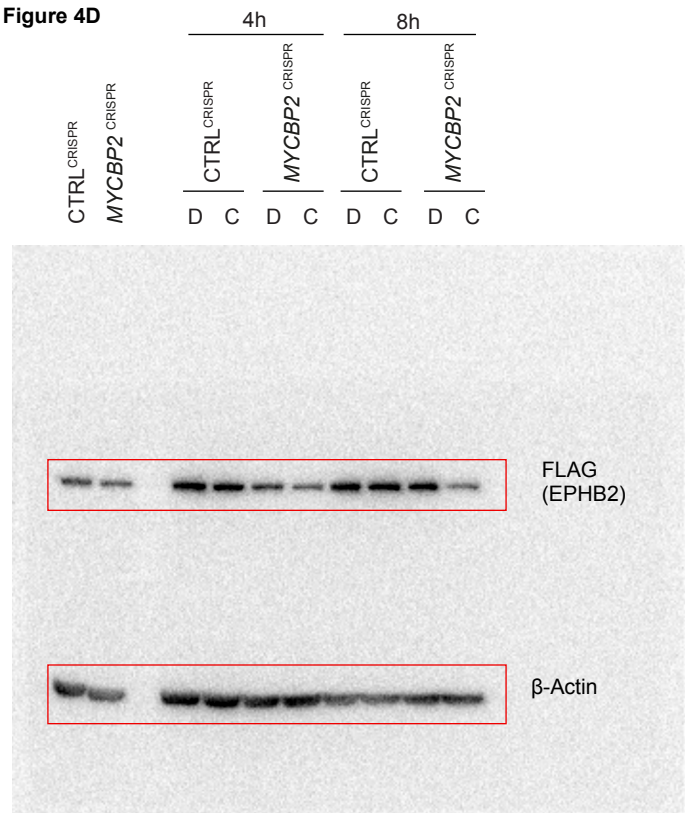

Supplement: Figure 4—source data 1. [file elife-89176-fig4-data1.zip › Figure 4 - source data 1/Figure 4-source data1.pdf]

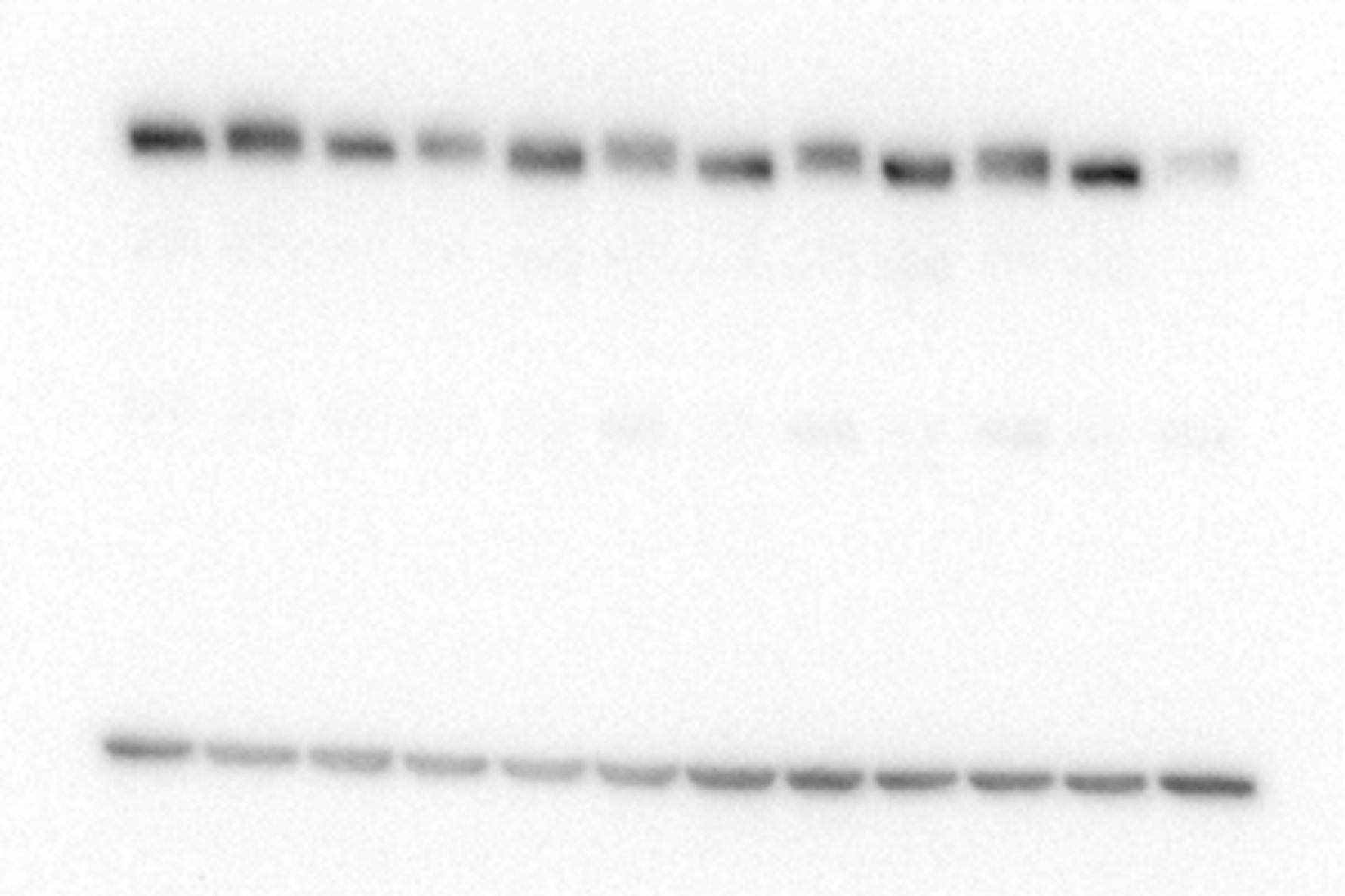

Supplement: Figure 4—source data 2. [file elife-89176-fig4-data2.zip › Figure 4 - source data 2/Figure 4F - FLAG(EPHB2) and Actin 1h.tif]

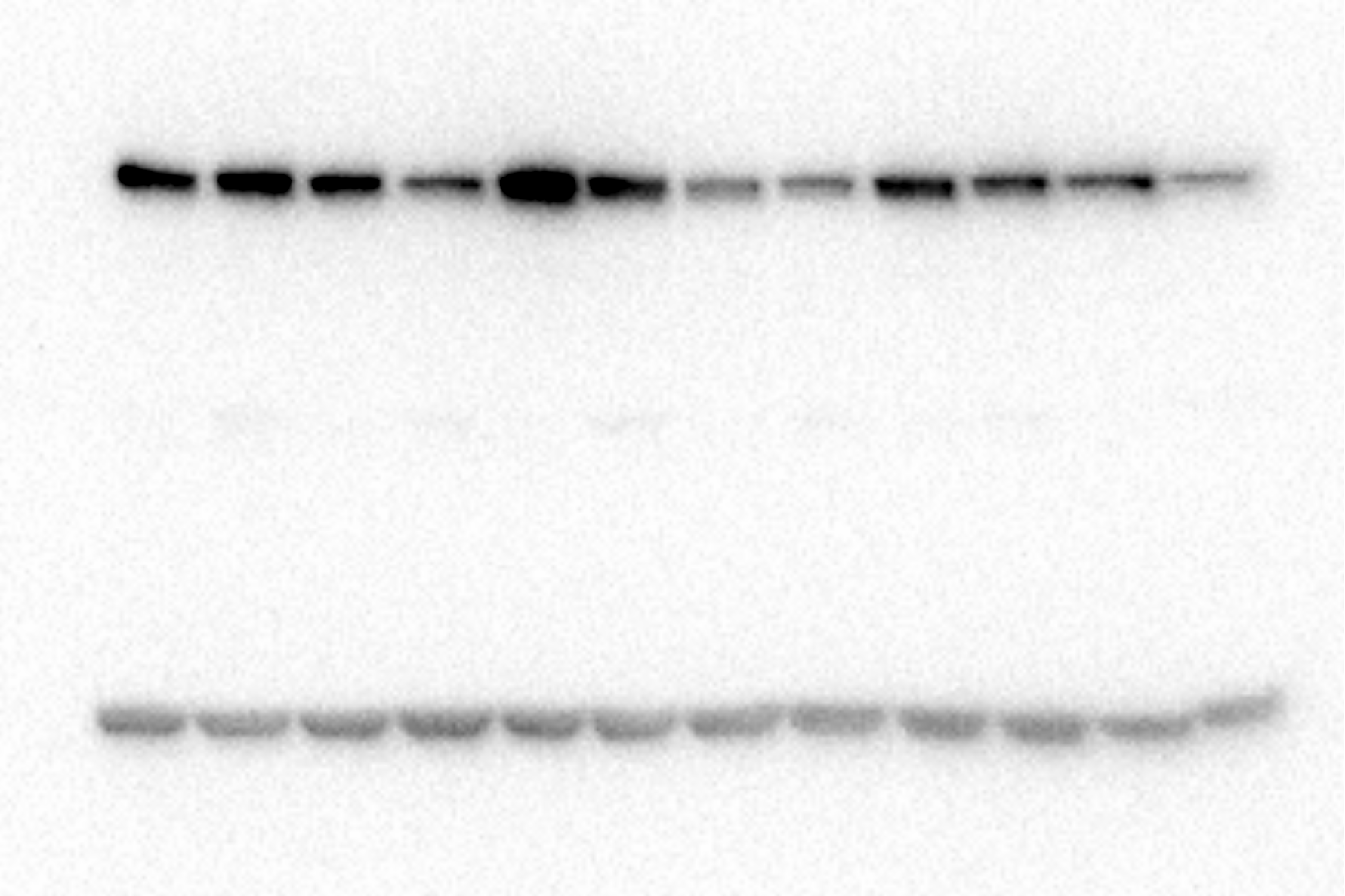

Supplement: Figure 4—source data 2. [file elife-89176-fig4-data2.zip › Figure 4 - source data 2/Figure 4F - FLAG(EPHB2) and Actin 2h.tif]

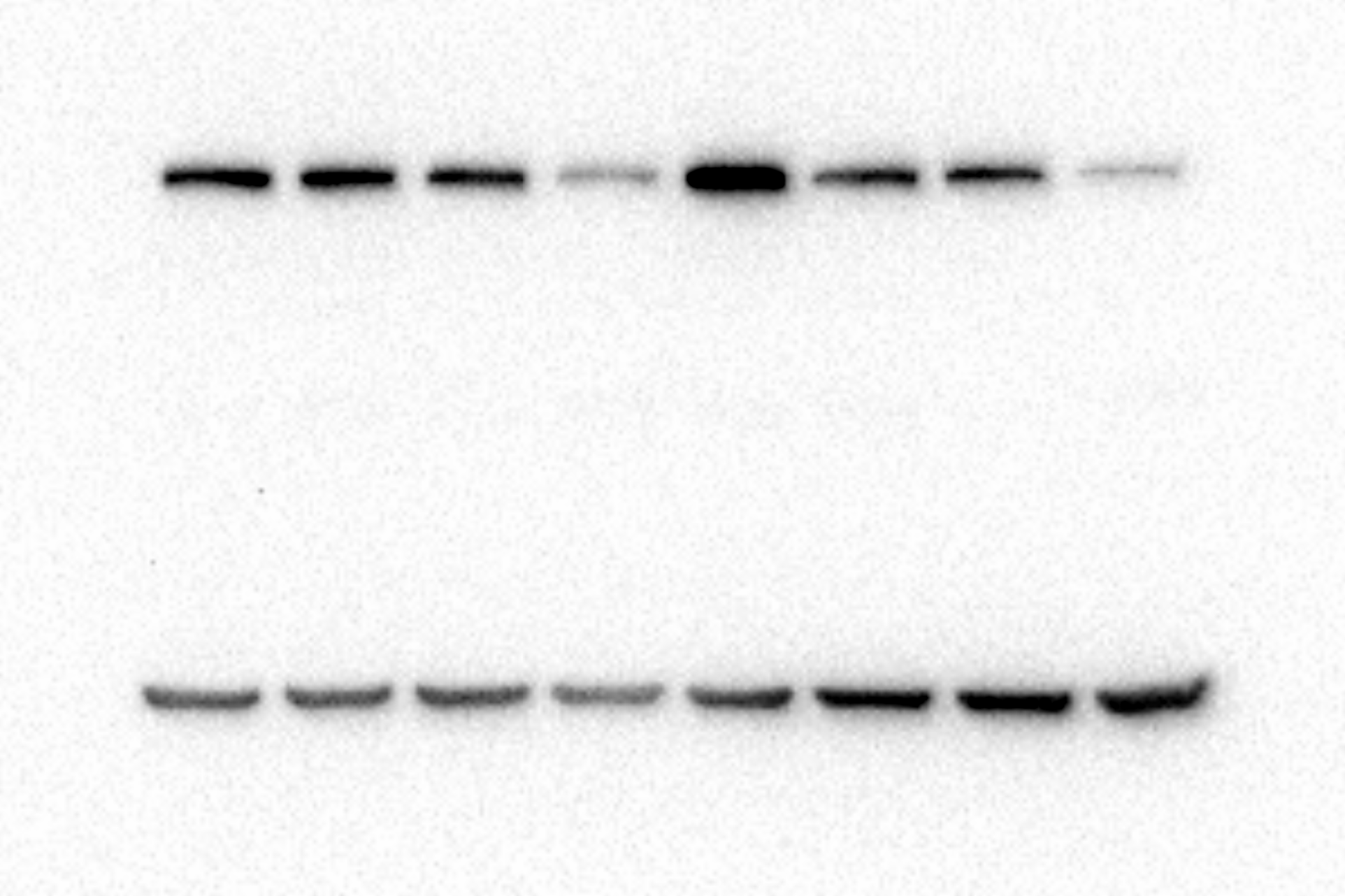

Supplement: Figure 4—source data 2. [file elife-89176-fig4-data2.zip › Figure 4 - source data 2/Figure 4F - FLAG(EPHB2) and Actin 4h 8h.tif]

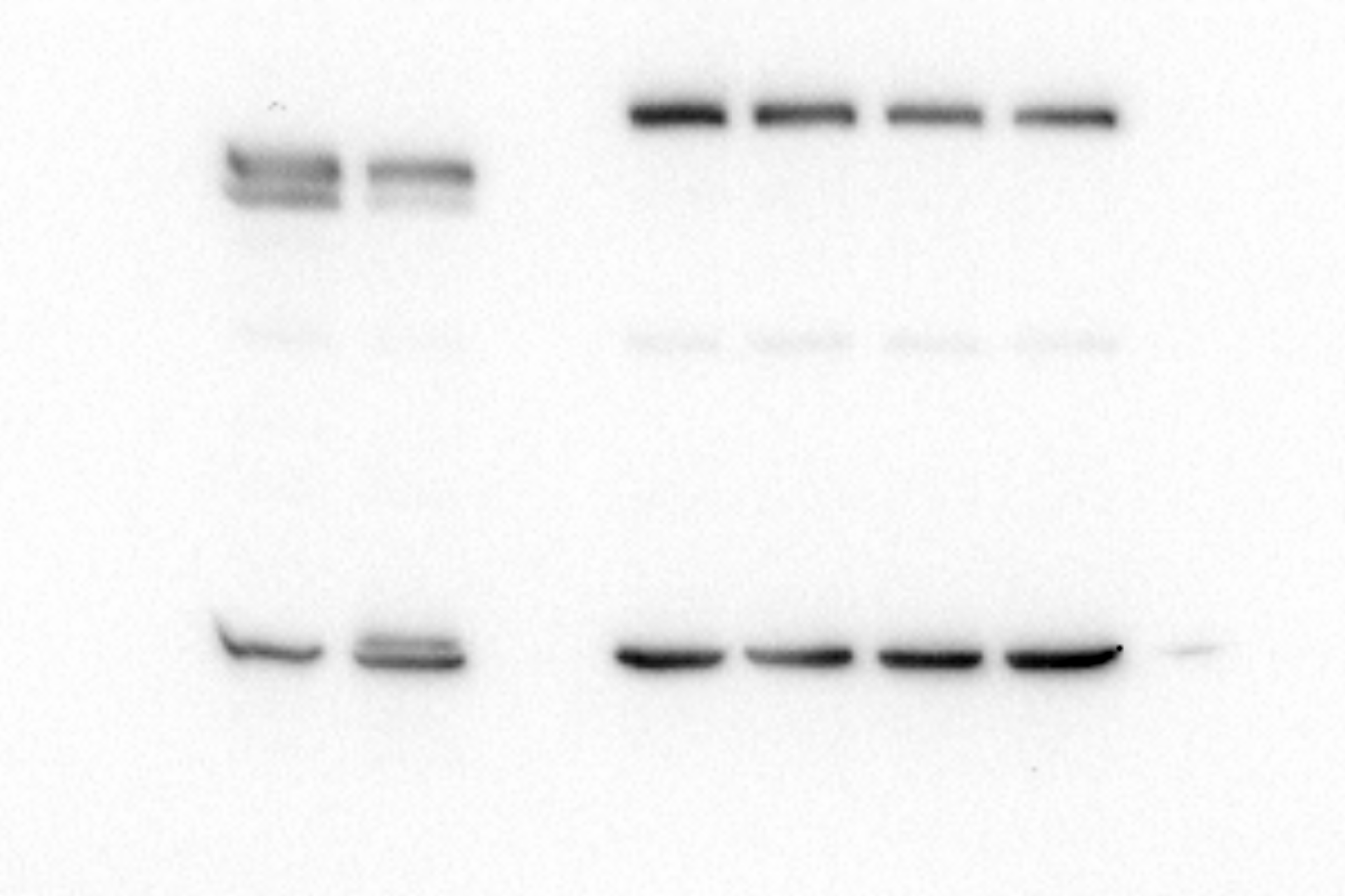

Supplement: Figure 4—source data 2. [file elife-89176-fig4-data2.zip › Figure 4 - source data 2/Figure 4F- FLAG(EPHB2) and Actin no treatment.tif]

Figure 4F

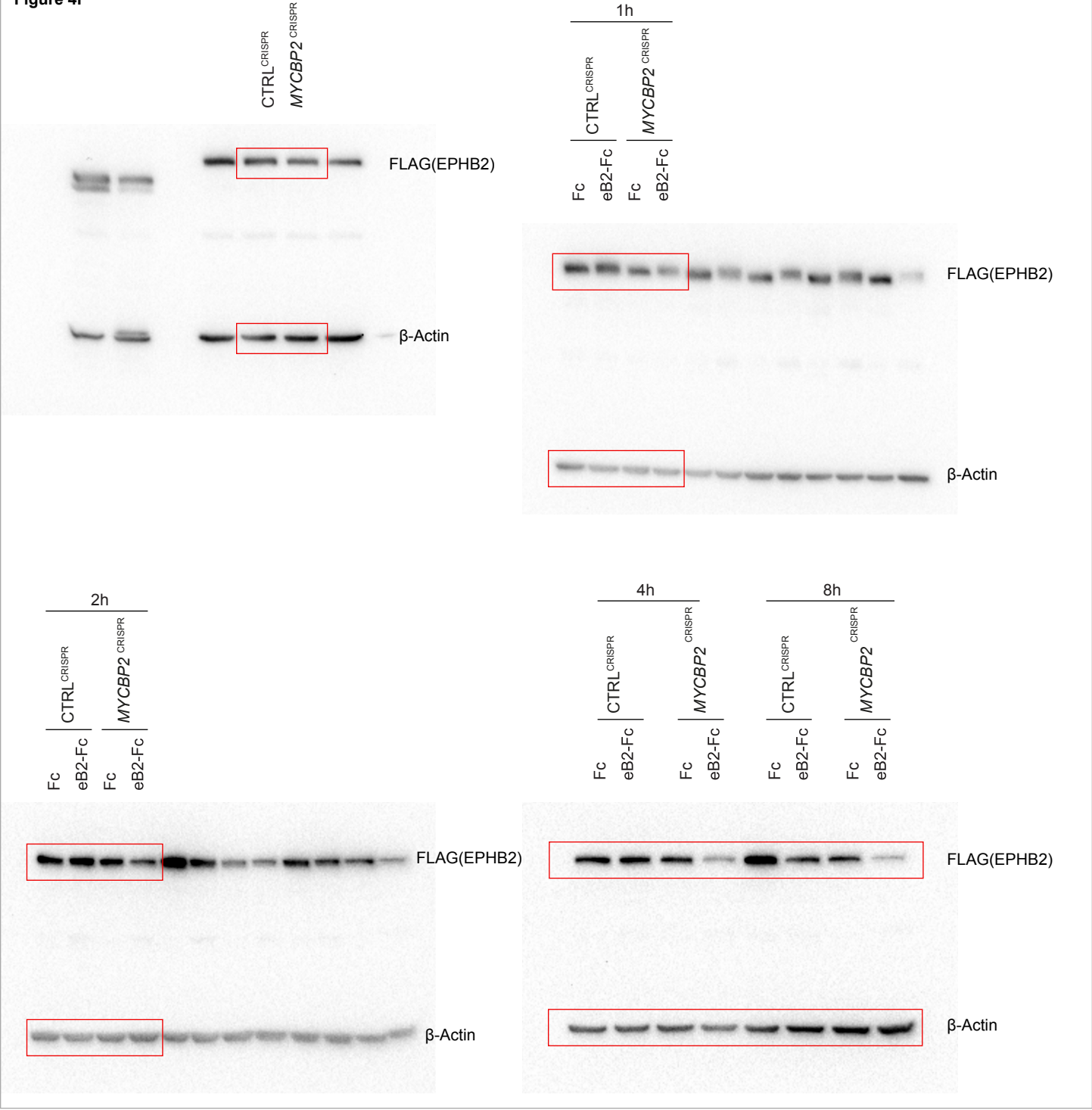

Supplement: Figure 4—source data 2. [file elife-89176-fig4-data2.zip › Figure 4 - source data 2/Figure 4-source data 2.pdf]

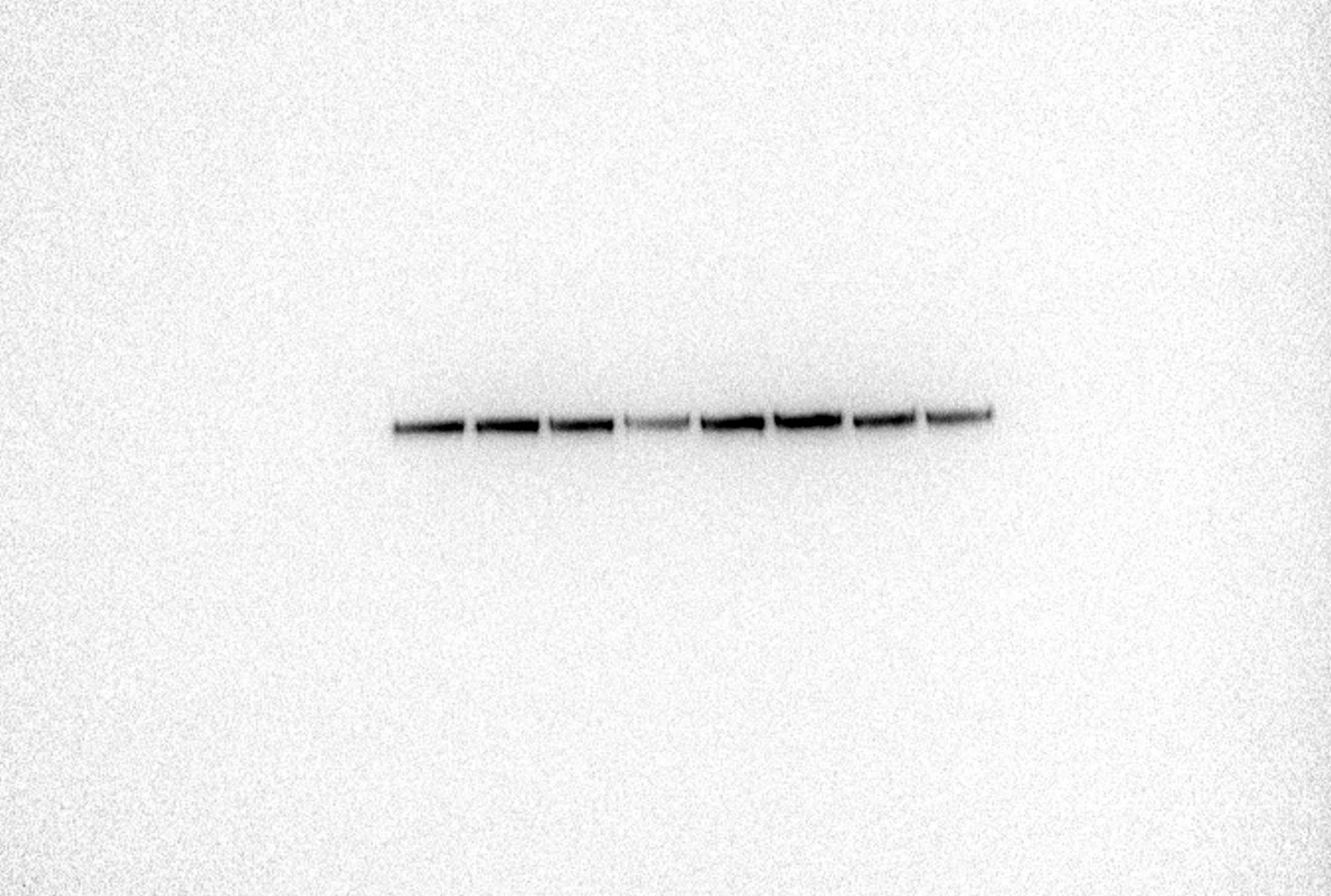

Supplement: Figure 4—source data 3. [file elife-89176-fig4-data3.zip › Figure 4 - source data 3/Figure 4H - FLAG(EPHB2).tif]

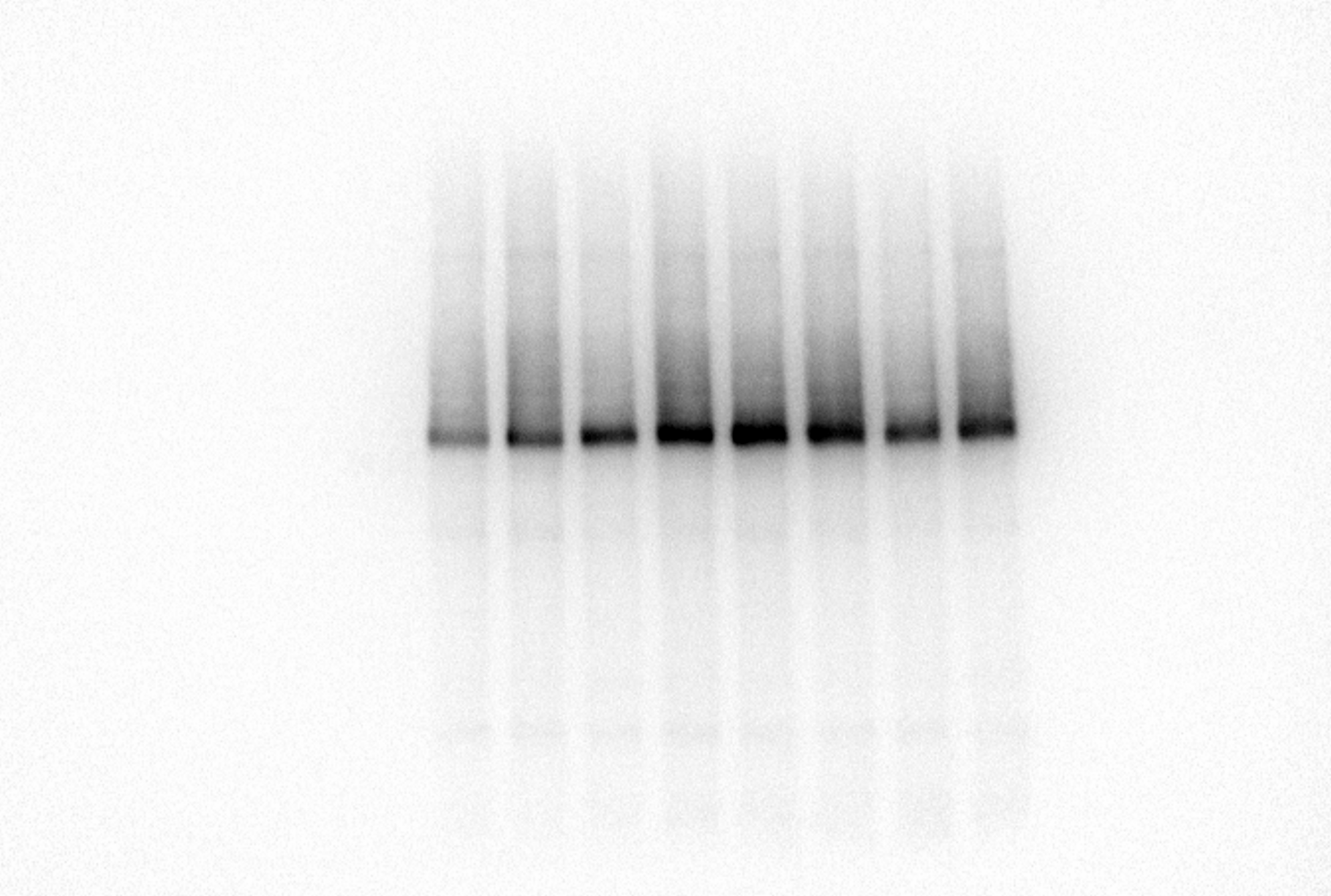

Supplement: Figure 4—source data 3. [file elife-89176-fig4-data3.zip › Figure 4 - source data 3/Figure 4H - Ub-EPHB2.tif]

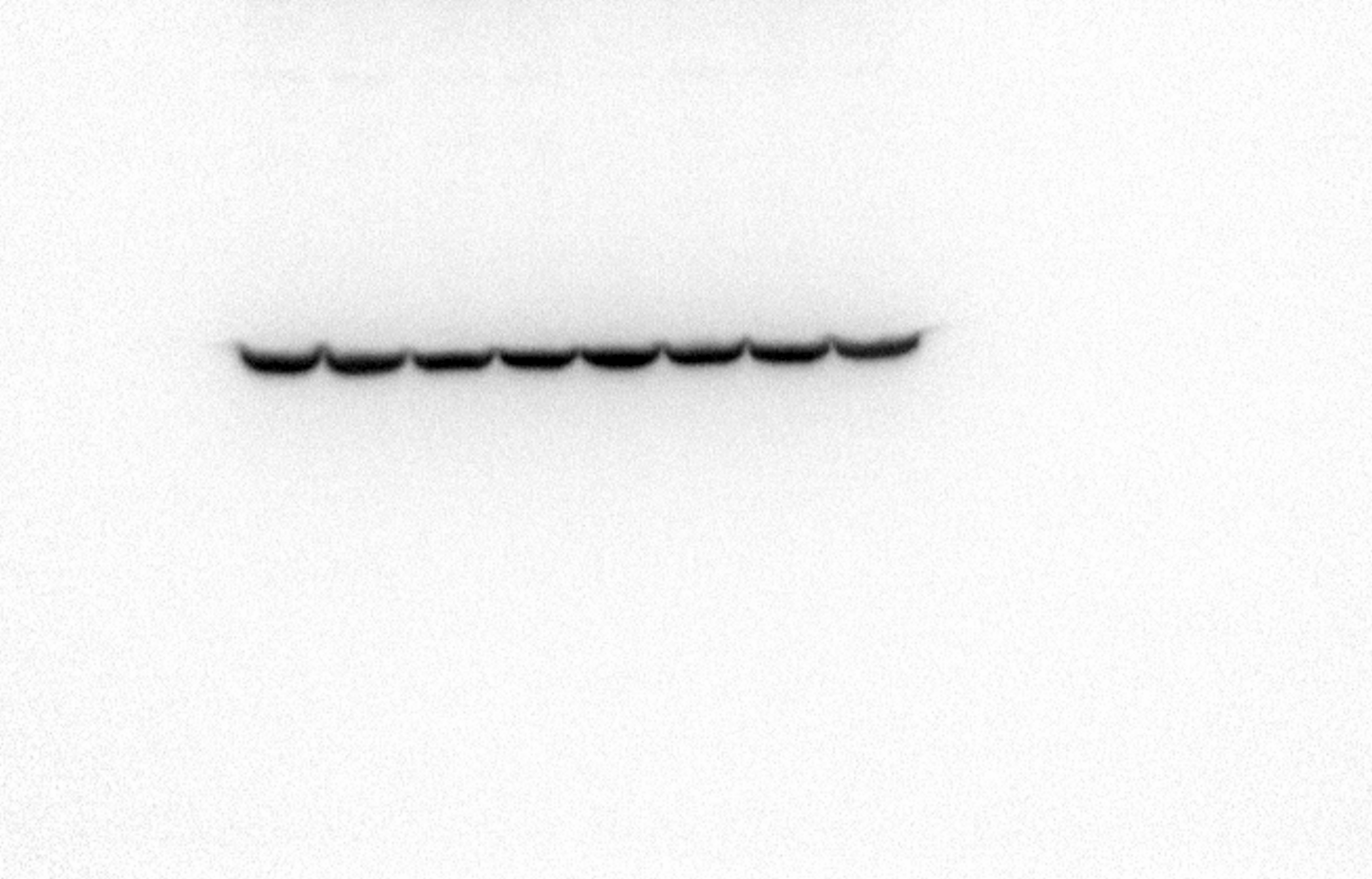

Supplement: Figure 4—source data 3. [file elife-89176-fig4-data3.zip › Figure 4 - source data 3/Figure 4J - Actin.tif]

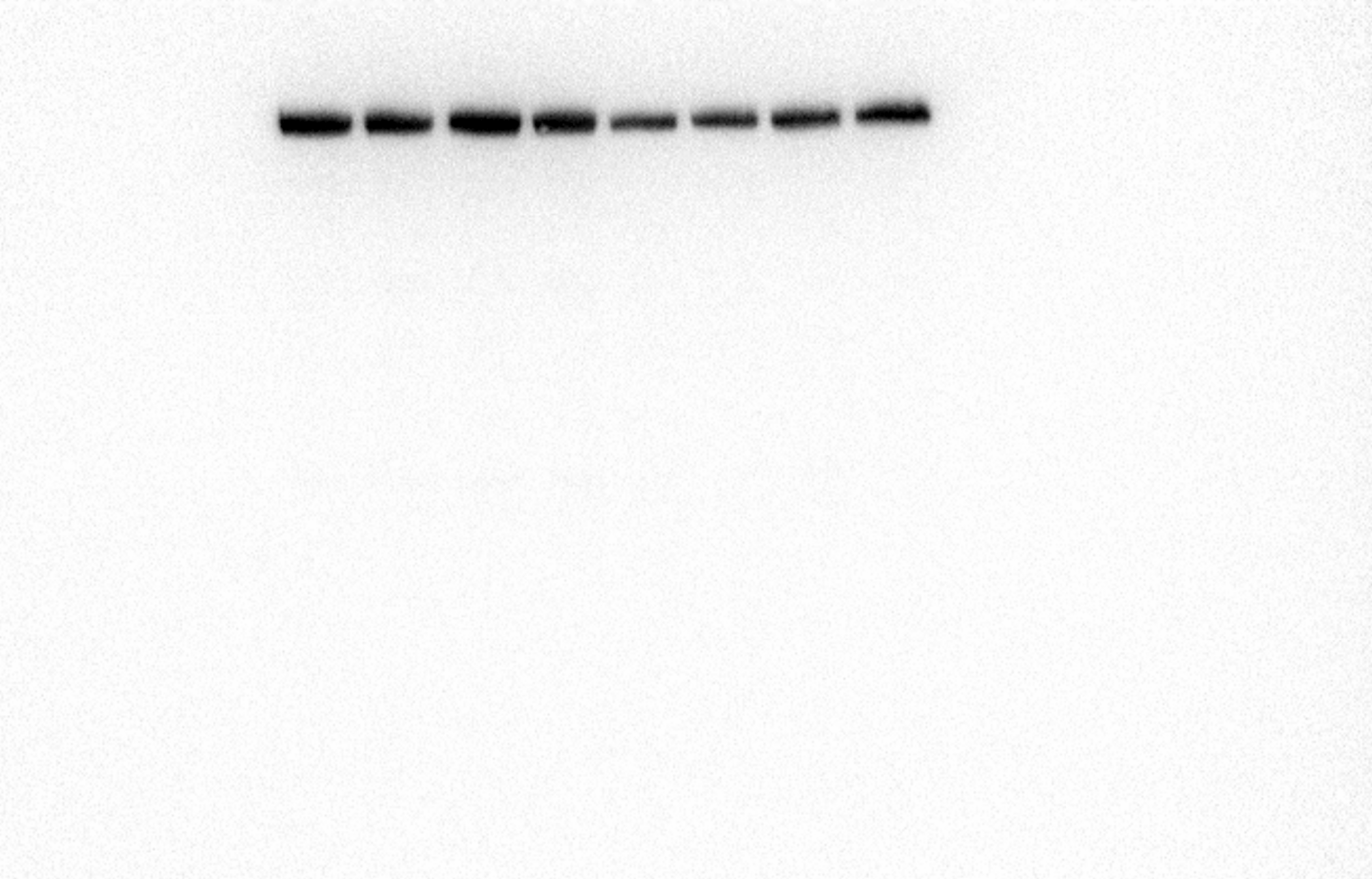

Supplement: Figure 4—source data 3. [file elife-89176-fig4-data3.zip › Figure 4 - source data 3/Figure 4J - FLAG(EPHB2).tif]

Figure 4H

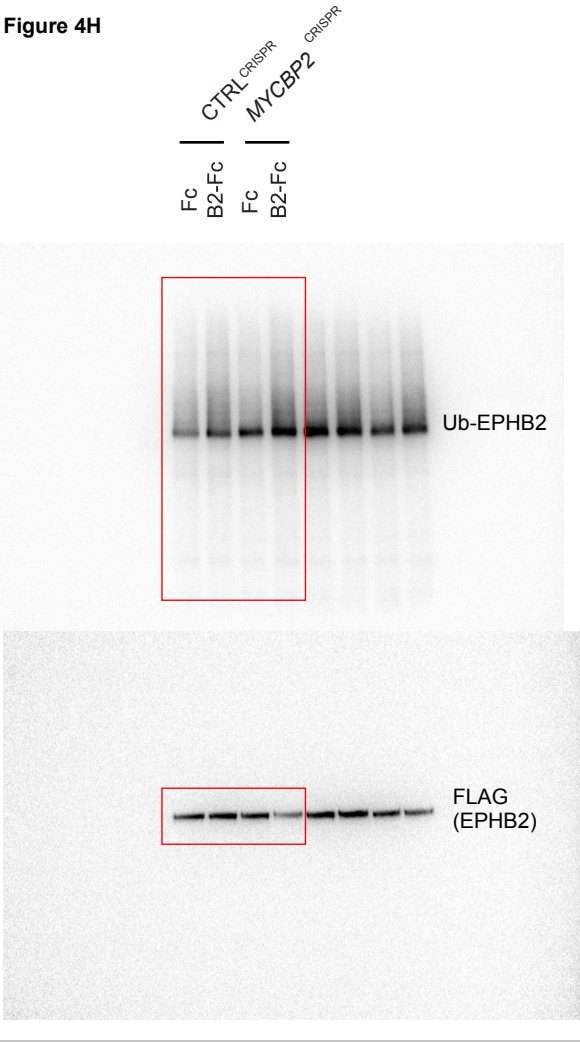

Figure 4J

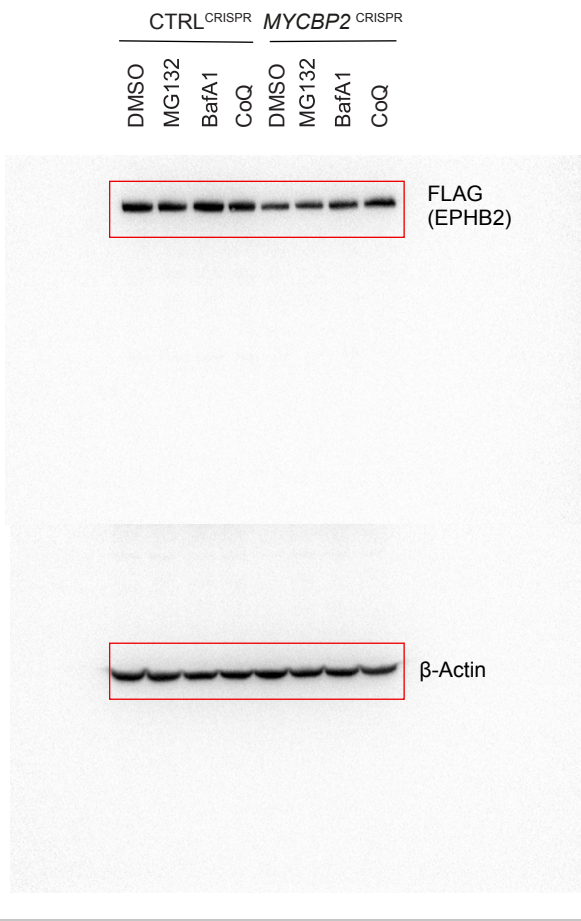

Supplement: Figure 4—source data 3. [file elife-89176-fig4-data3.zip › Figure 4 - source data 3/Figure 4-source data 3.pdf]

Figure 4L

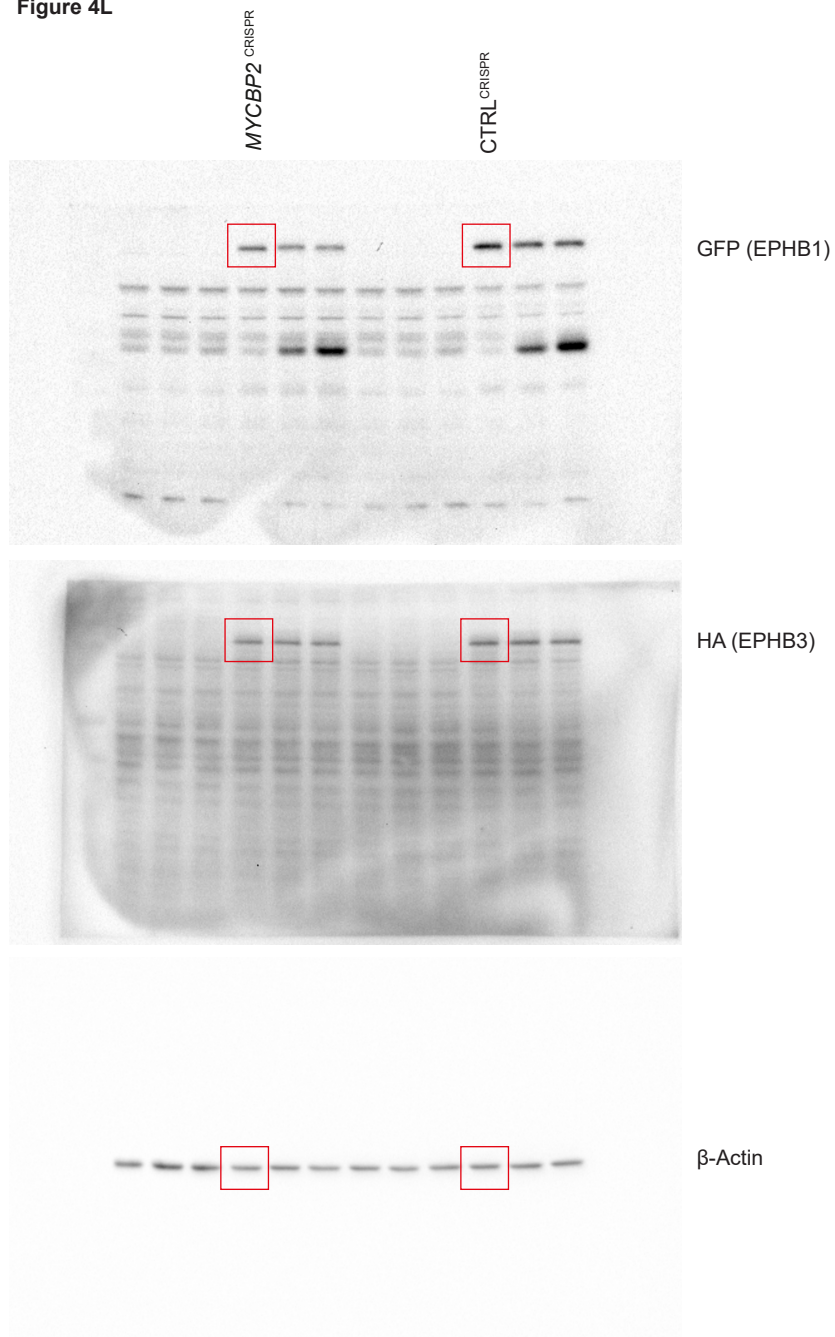

Supplement: Figure 4—source data 4. [file elife-89176-fig4-data4.zip › Figure 4 - source data 4/Figure 4 - source data 4.pdf]

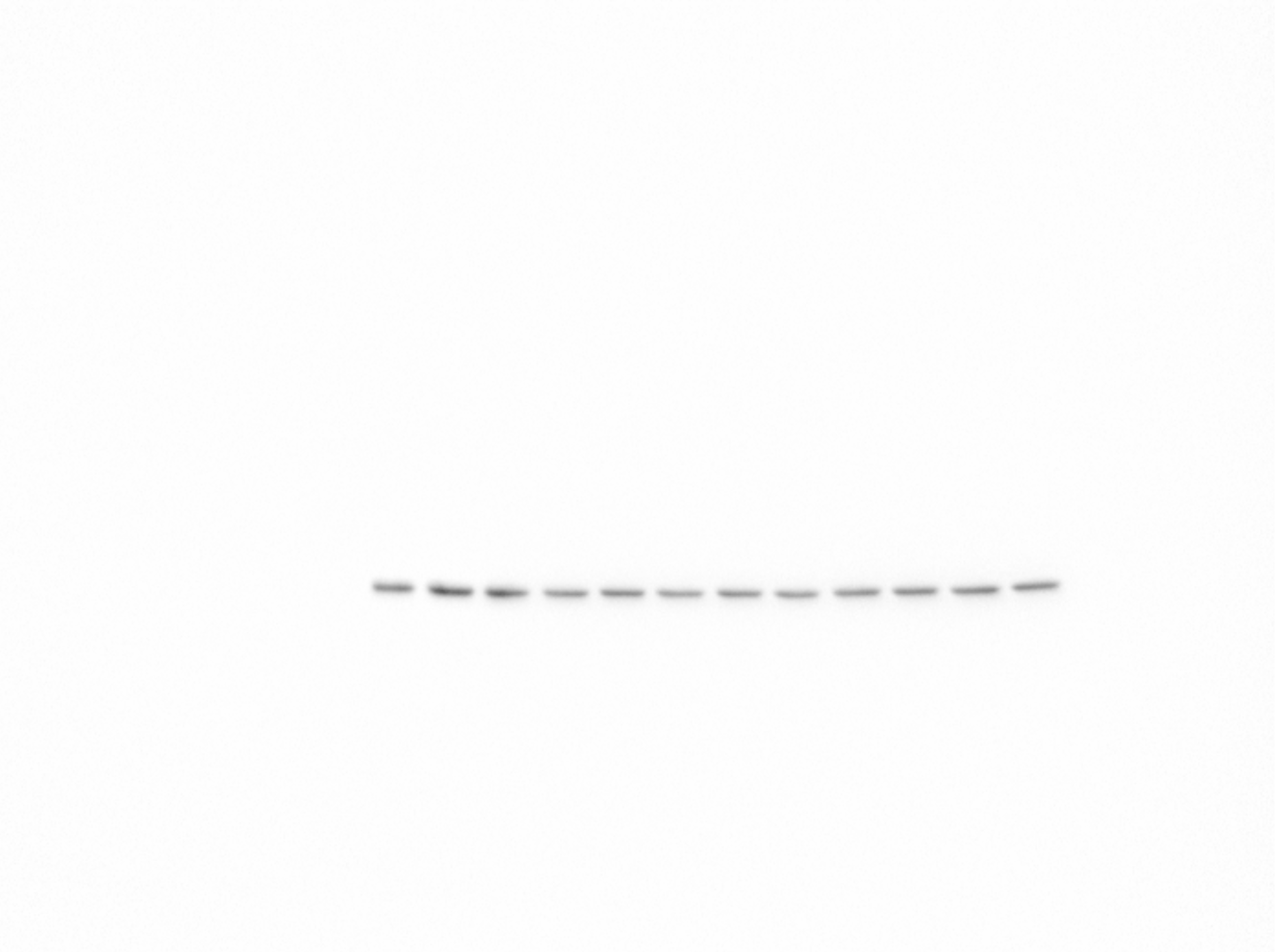

Supplement: Figure 4—source data 4. [file elife-89176-fig4-data4.zip › Figure 4 - source data 4/Figure 4L - B-actin.tif]

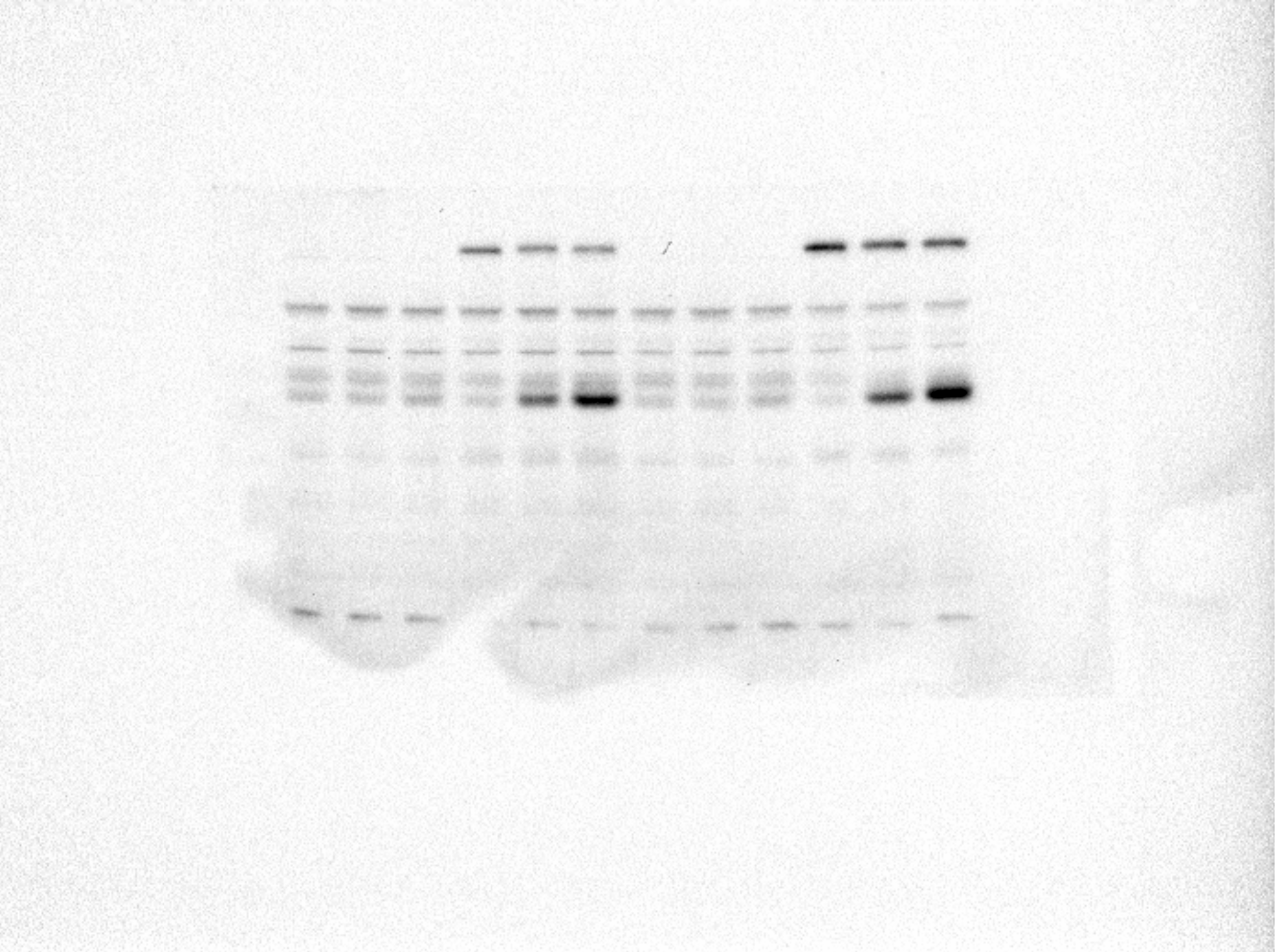

Supplement: Figure 4—source data 4. [file elife-89176-fig4-data4.zip › Figure 4 - source data 4/Figure 4L - GFP.tif]

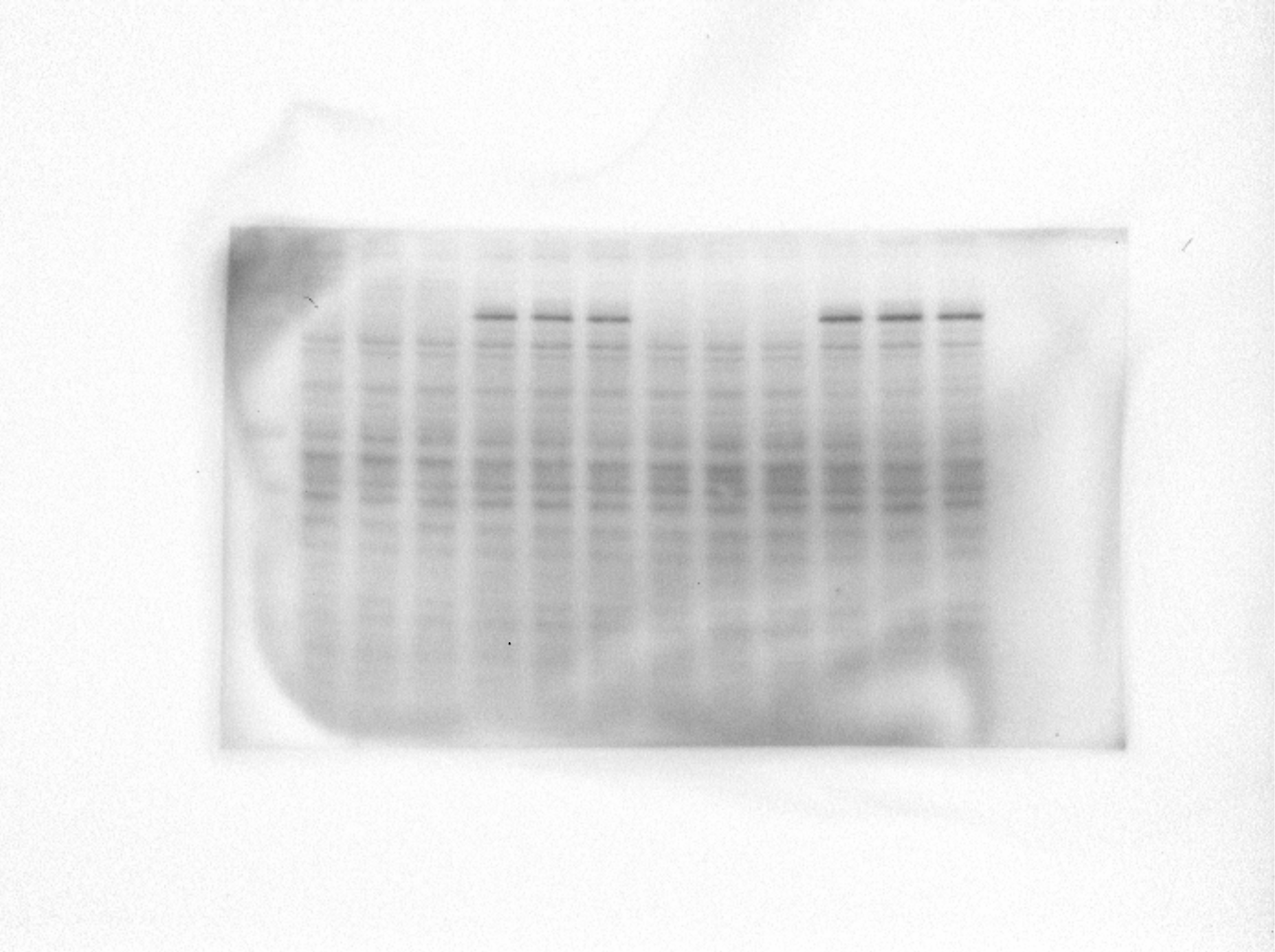

Supplement: Figure 4—source data 4. [file elife-89176-fig4-data4.zip › Figure 4 - source data 4/Figure 4L - HA.tif]

**Figure 5A**

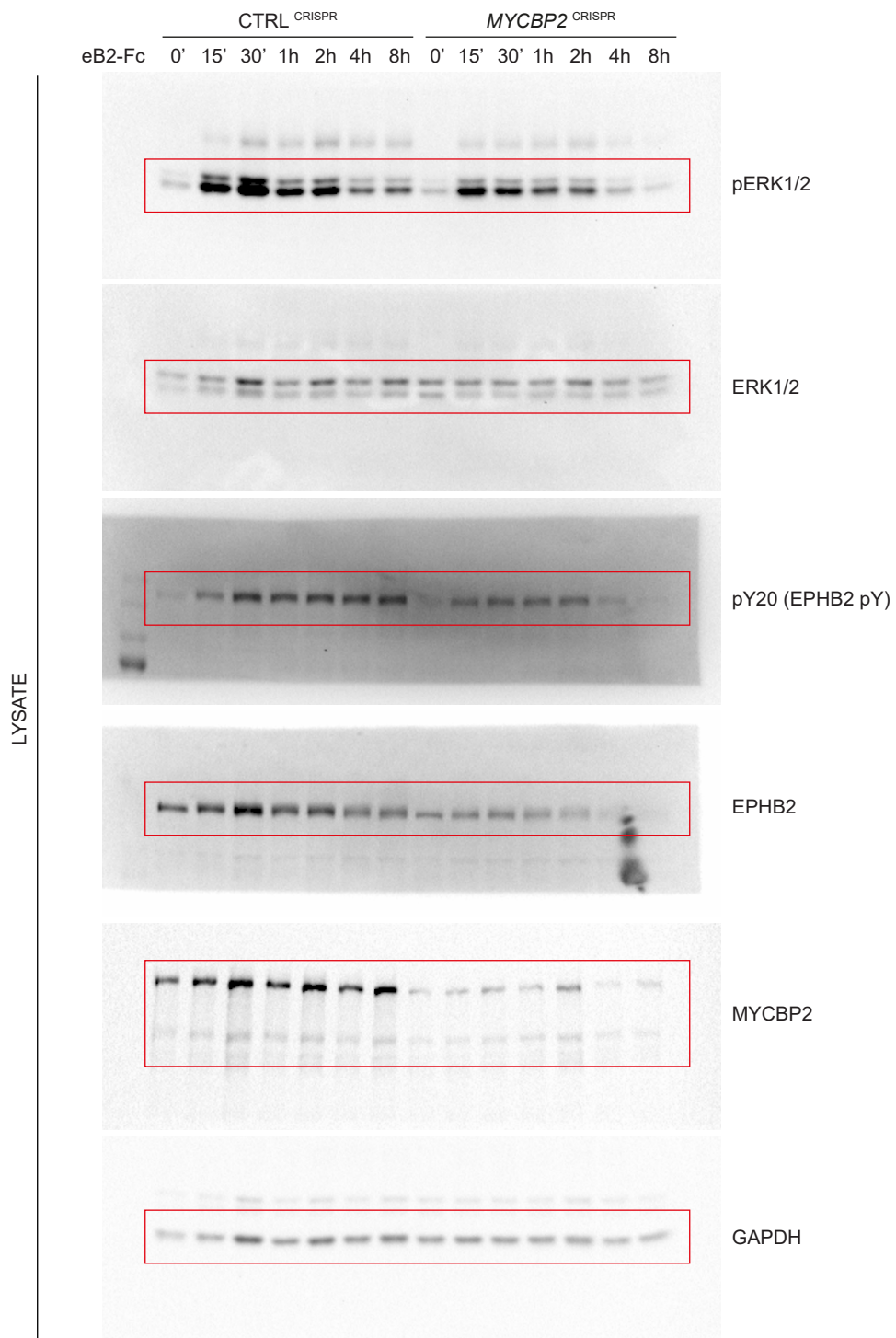

Supplement: Figure 5—source data 1. [file elife-89176-fig5-data1.zip › Figure 5 - source data 1/Figure 5 - source data 1.pdf]

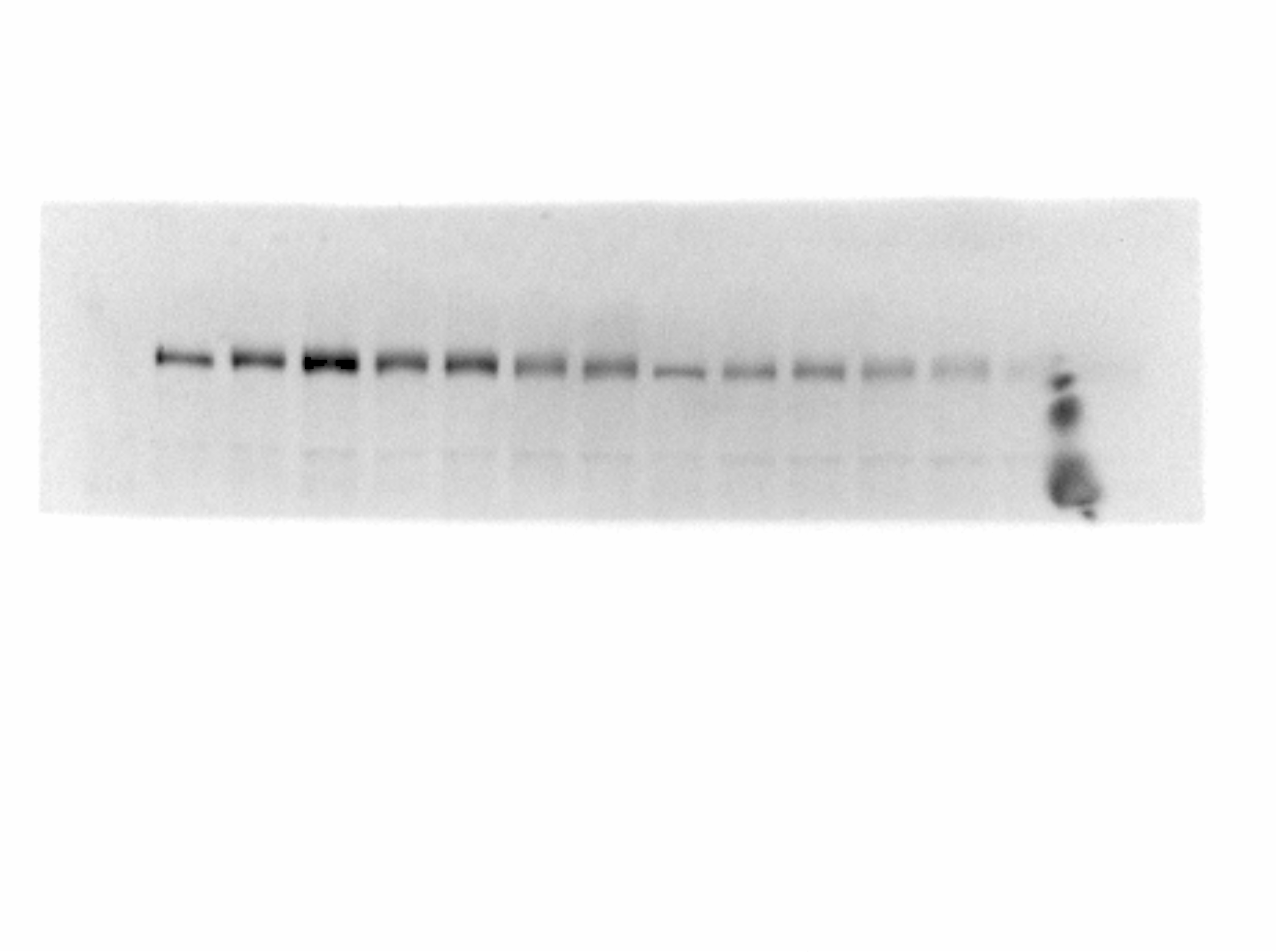

Supplement: Figure 5—source data 1. [file elife-89176-fig5-data1.zip › Figure 5 - source data 1/Figure 5A - EPHB2.tif]

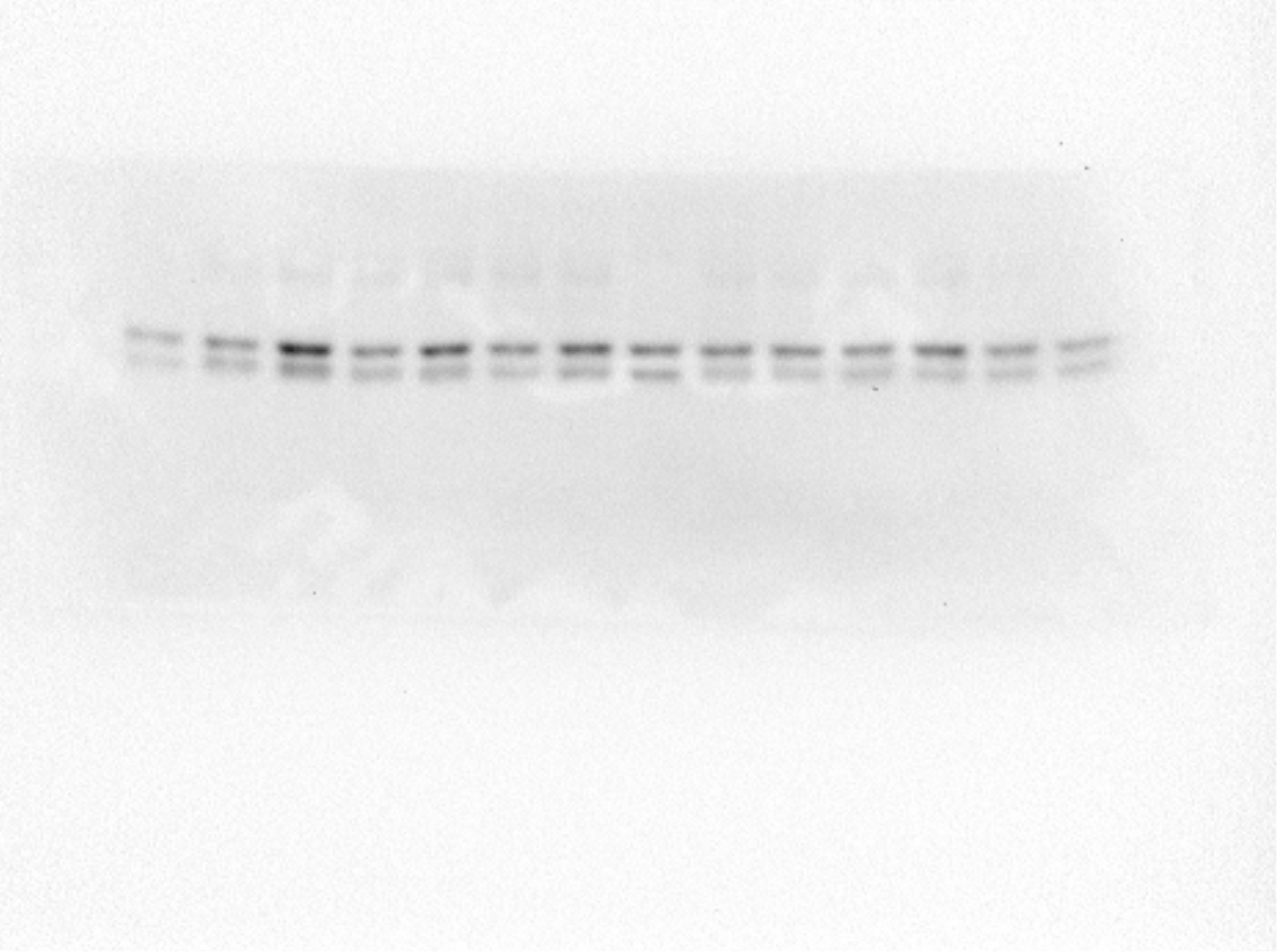

Supplement: Figure 5—source data 1. [file elife-89176-fig5-data1.zip › Figure 5 - source data 1/Figure 5A - ERK1&2.tif]

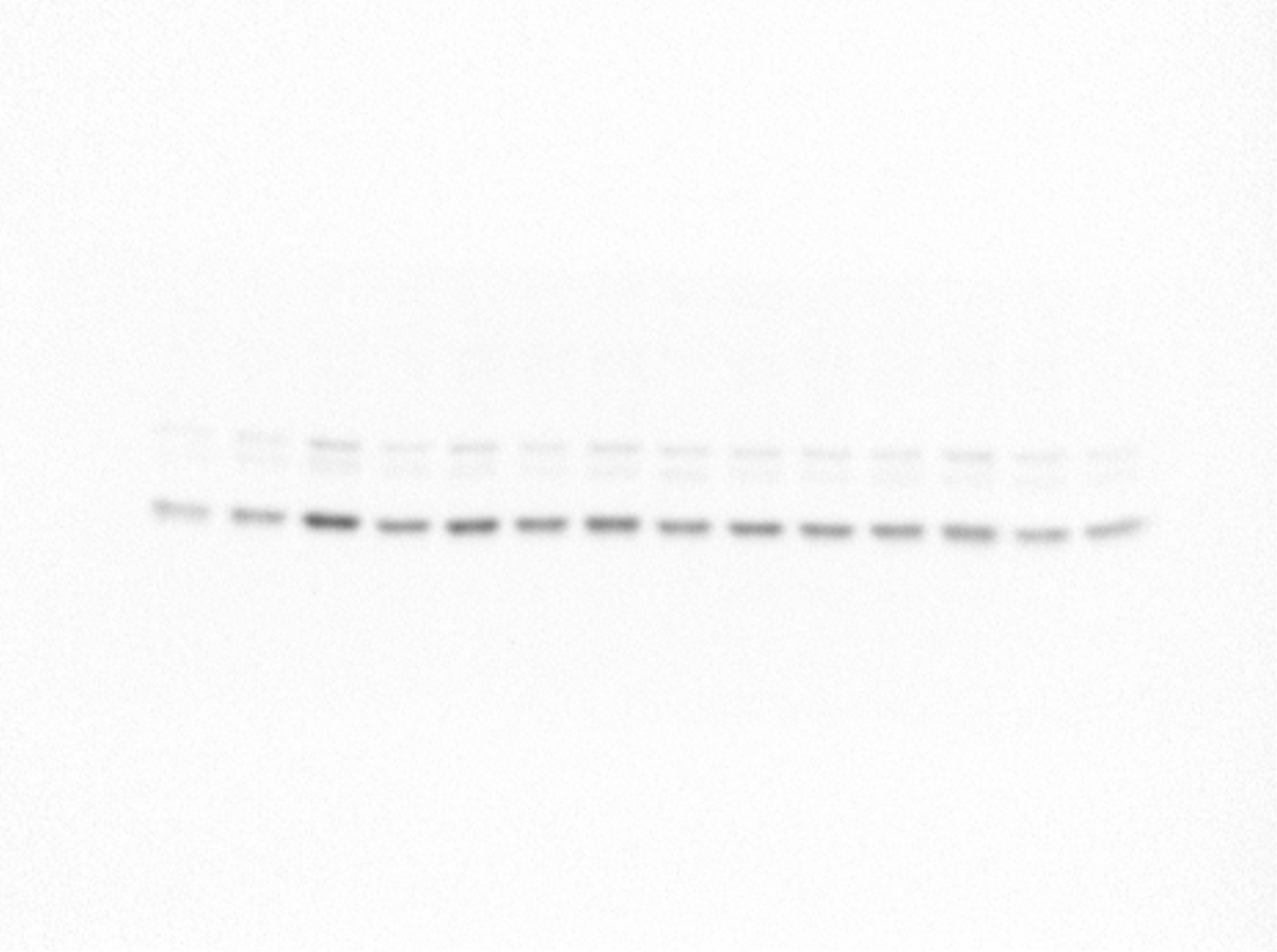

Supplement: Figure 5—source data 1. [file elife-89176-fig5-data1.zip › Figure 5 - source data 1/Figure 5A - GAPDH.tif]

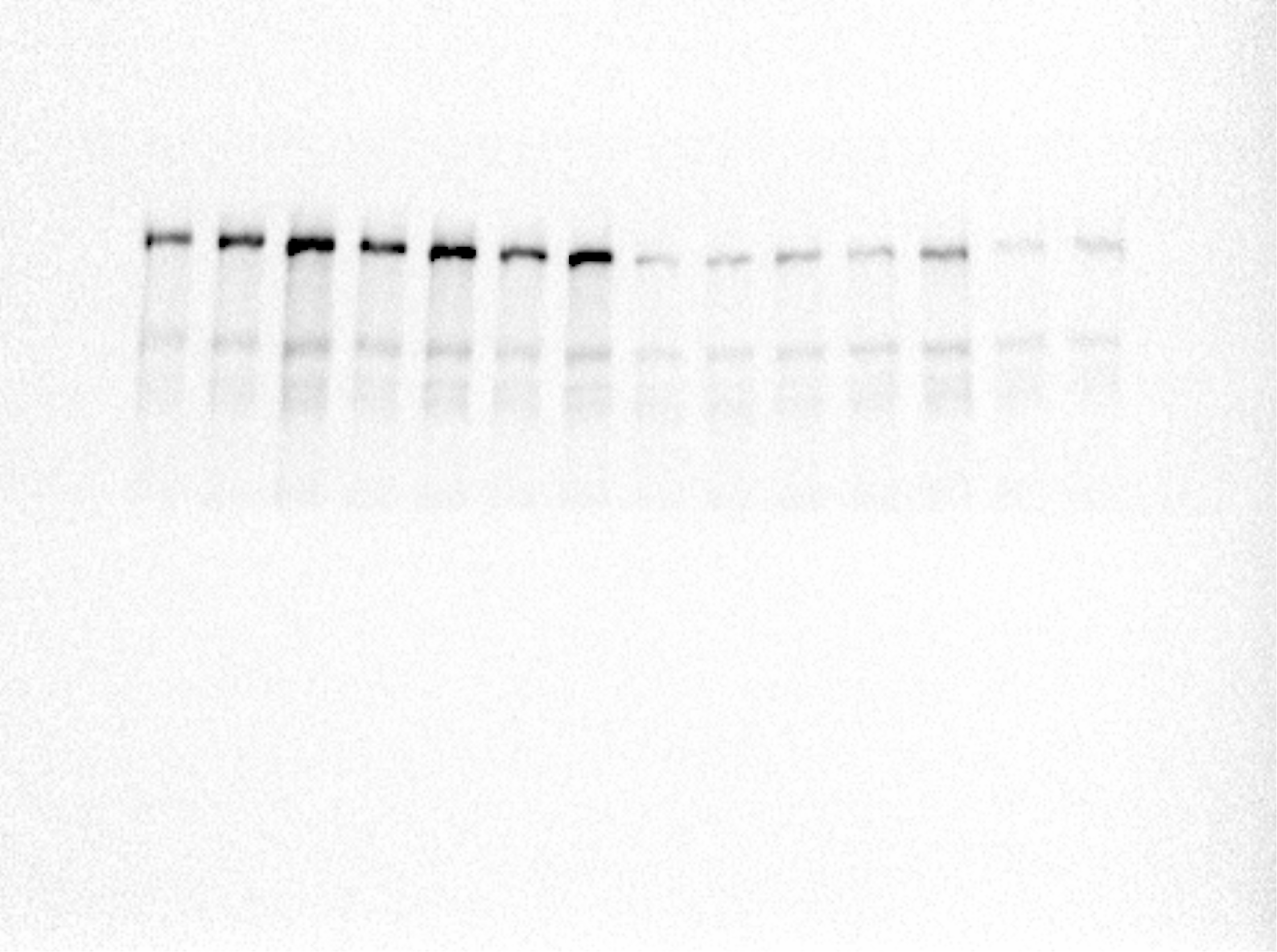

Supplement: Figure 5—source data 1. [file elife-89176-fig5-data1.zip › Figure 5 - source data 1/Figure 5A - MYCBP2.tif]

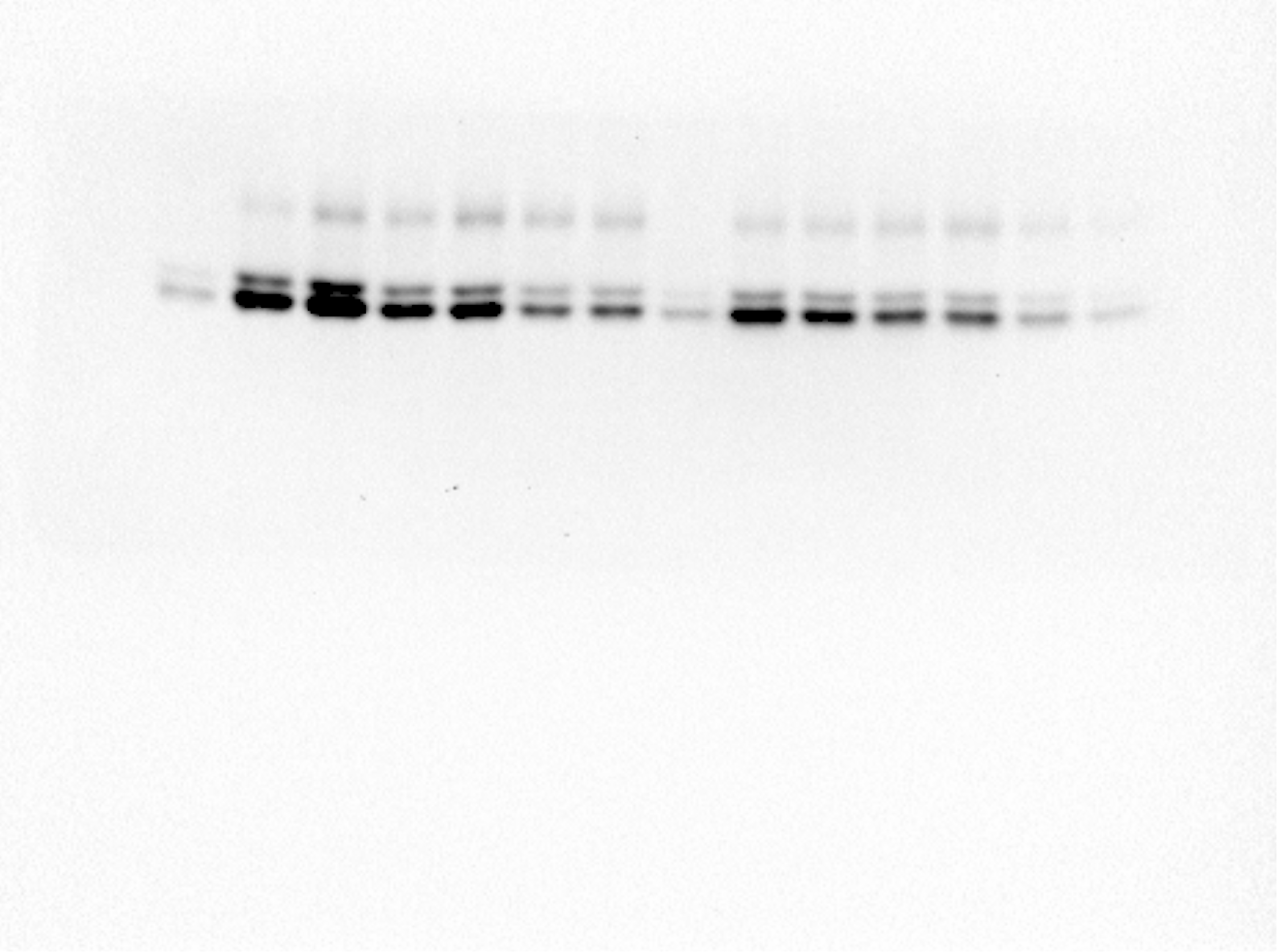

Supplement: Figure 5—source data 1. [file elife-89176-fig5-data1.zip › Figure 5 - source data 1/Figure 5A - pERK1&2.tif]

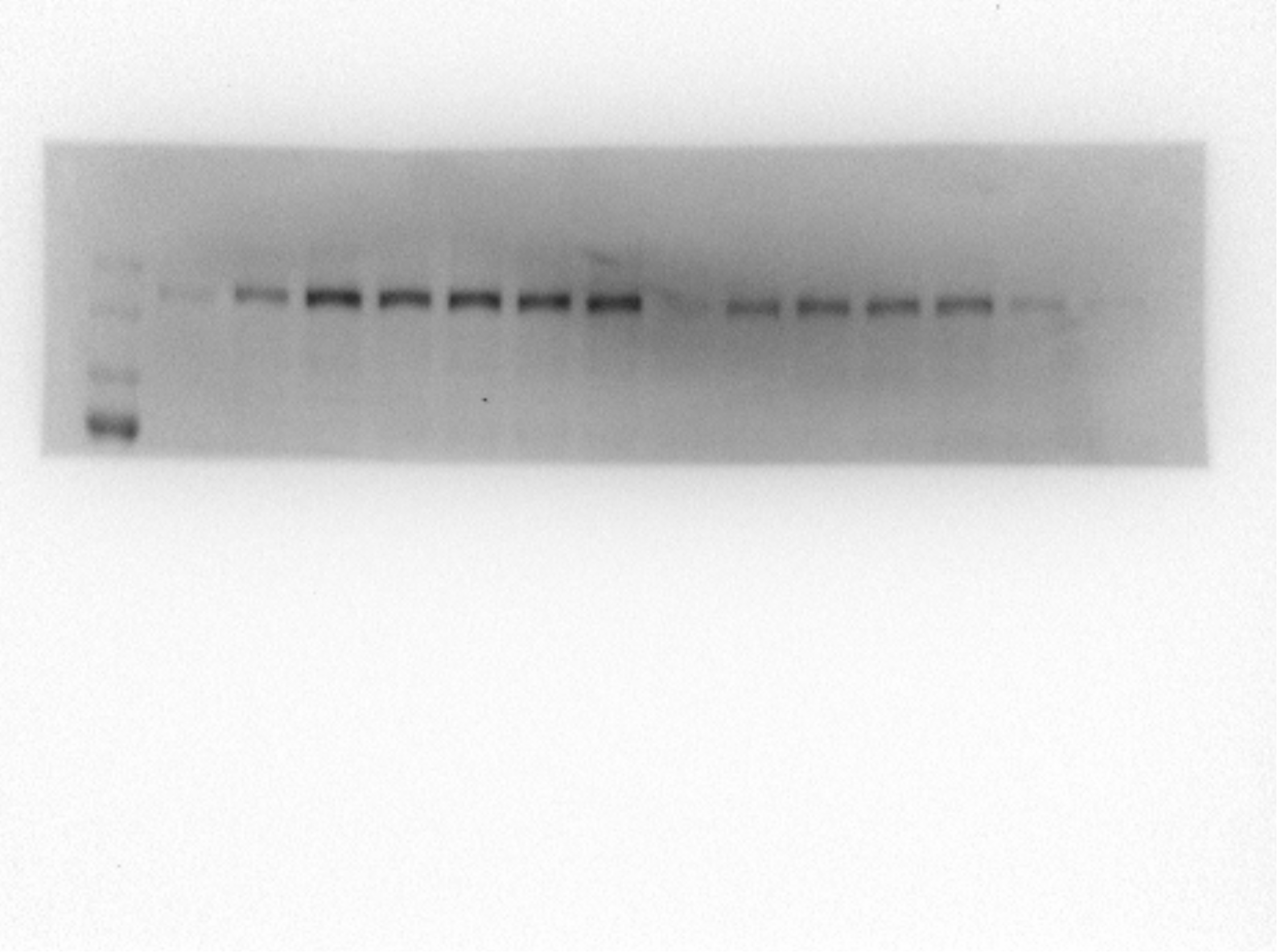

Supplement: Figure 5—source data 1. [file elife-89176-fig5-data1.zip › Figure 5 - source data 1/Figure 5A - pY20.tif]

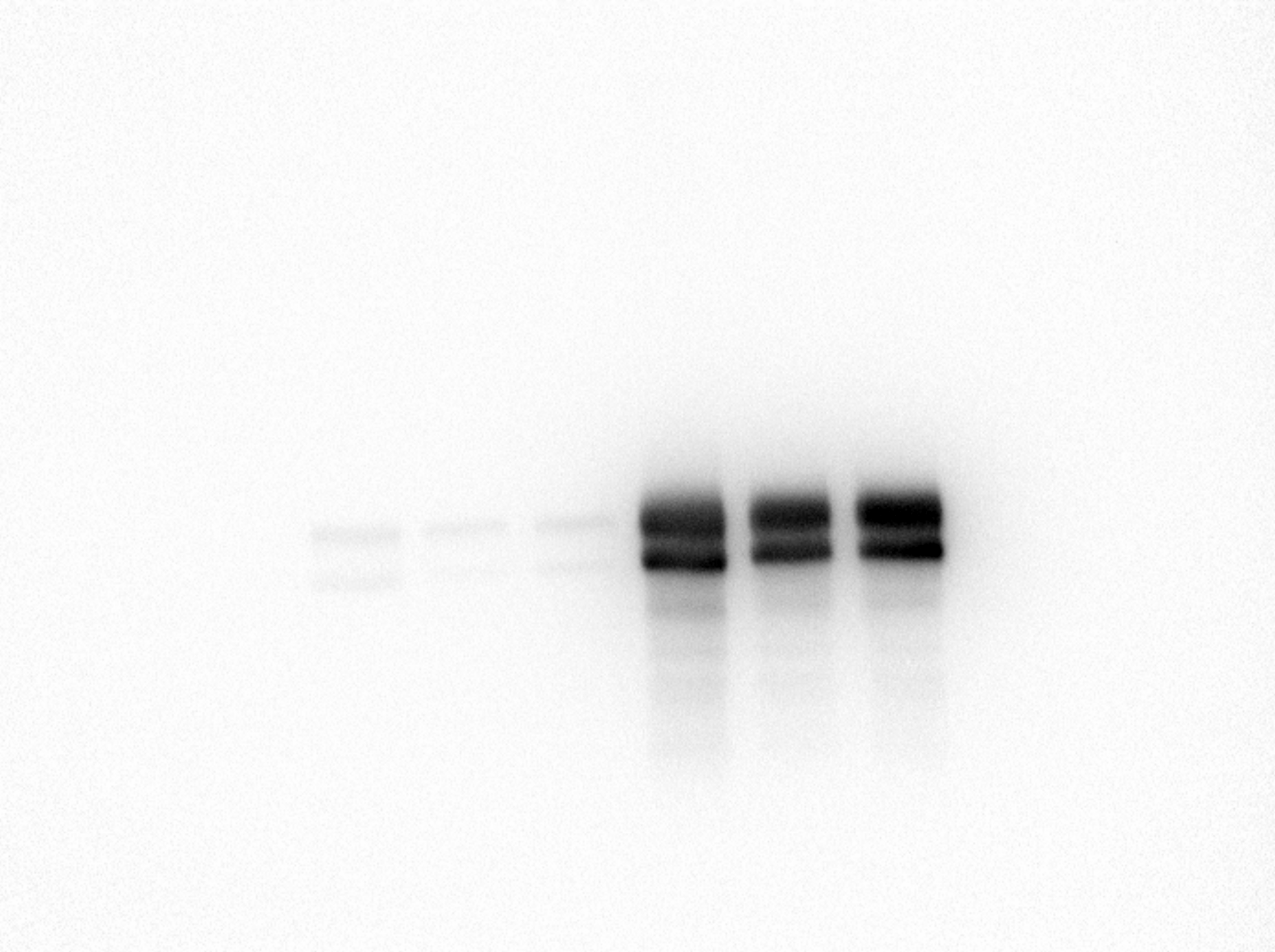

Supplement: Figure 6—source data 1. [file elife-89176-fig6-data1.zip › Figure 6 - source data 1/Figure 6B - FLAG(EPHB2) IP.tif]

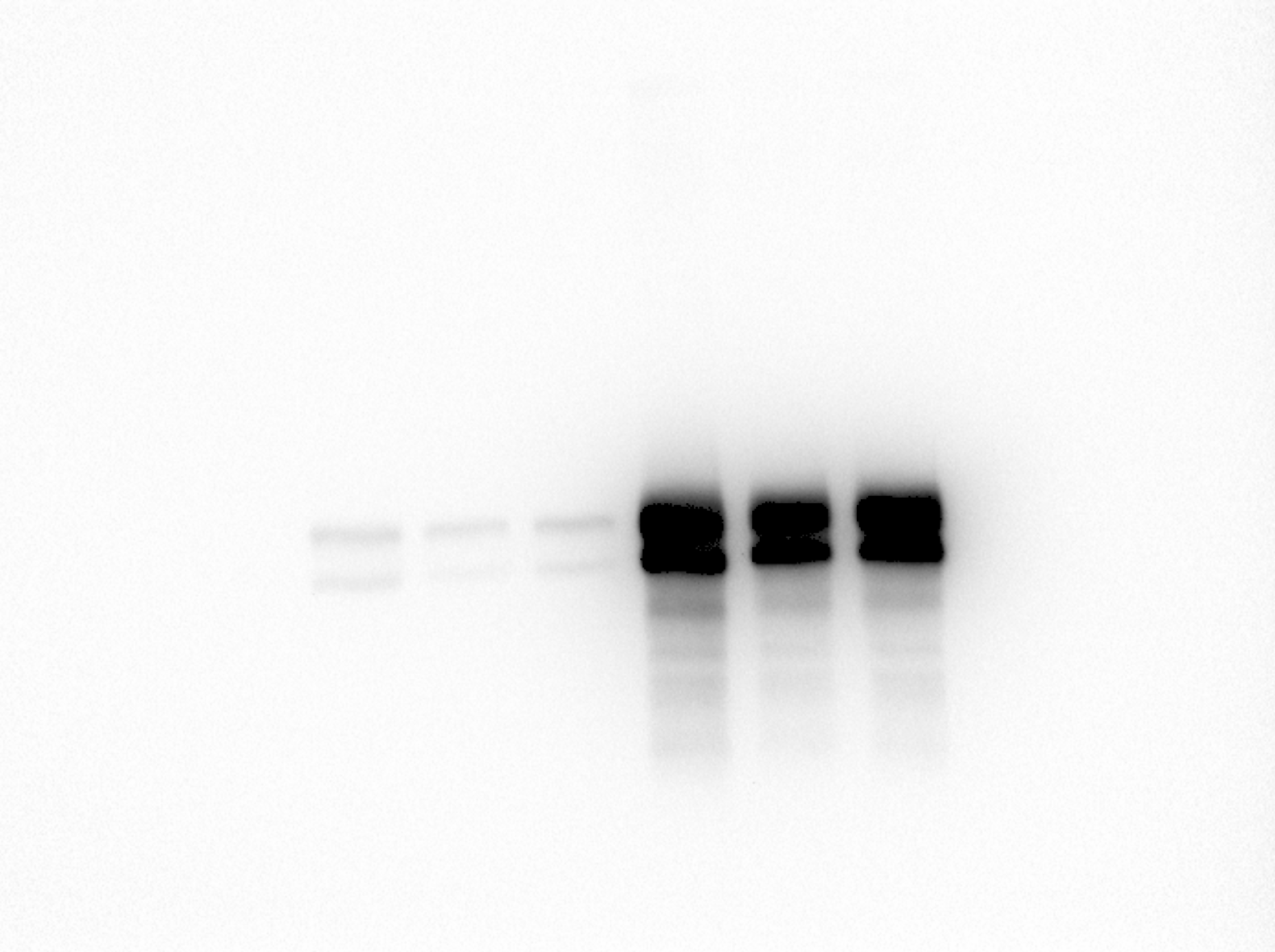

Supplement: Figure 6—source data 1. [file elife-89176-fig6-data1.zip › Figure 6 - source data 1/Figure 6B - FLAG(EPHB2) Lysate.tif]

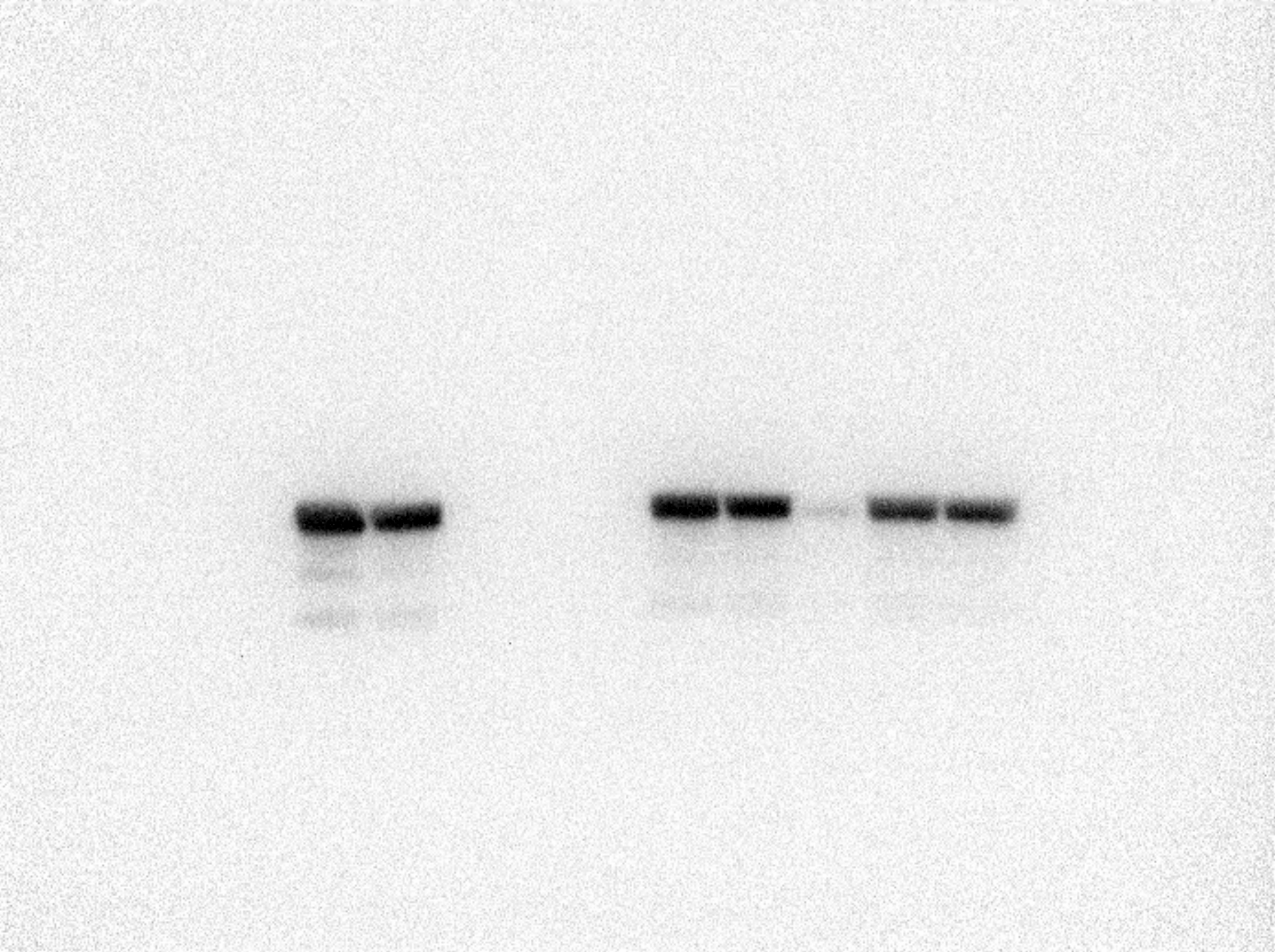

Supplement: Figure 6—source data 1. [file elife-89176-fig6-data1.zip › Figure 6 - source data 1/Figure 6B - GFP Lysate.tif]

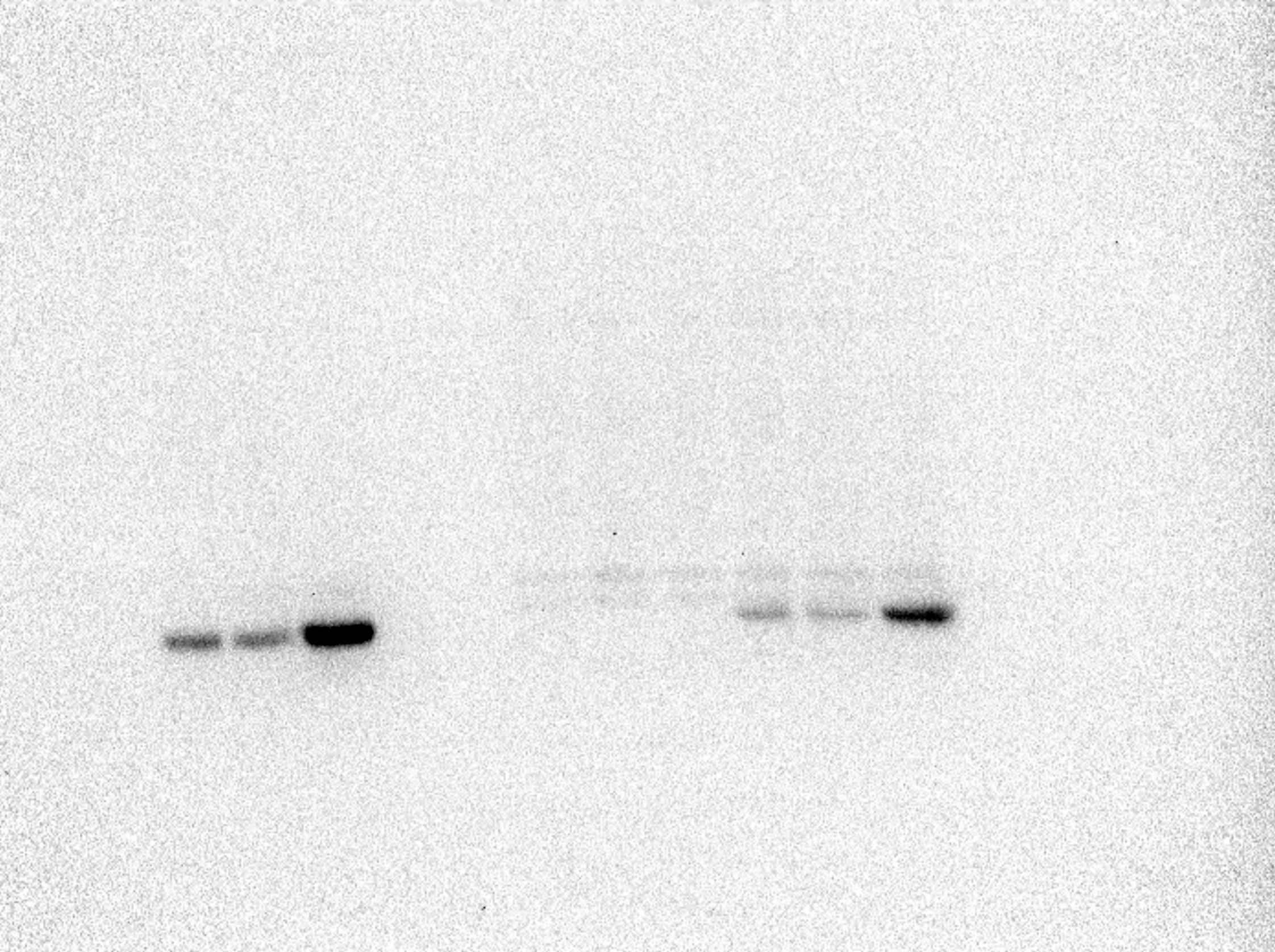

Supplement: Figure 6—source data 1. [file elife-89176-fig6-data1.zip › Figure 6 - source data 1/Figure 6B - MYC Lysate.tif]

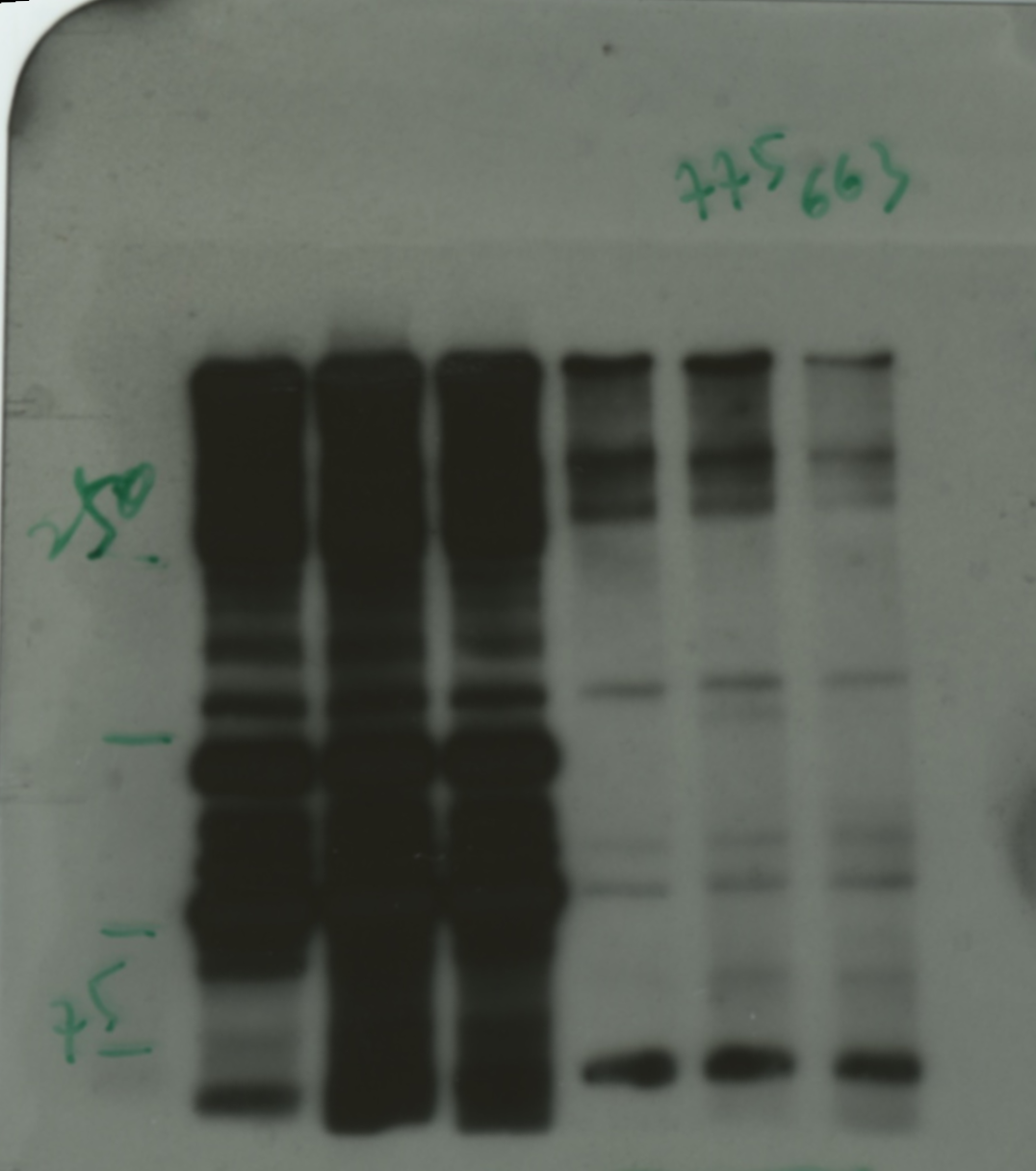

Supplement: Figure 6—source data 1. [file elife-89176-fig6-data1.zip › Figure 6 - source data 1/Figure 6B - MYCBP2 IP Lysate.tif]

Figure 6B

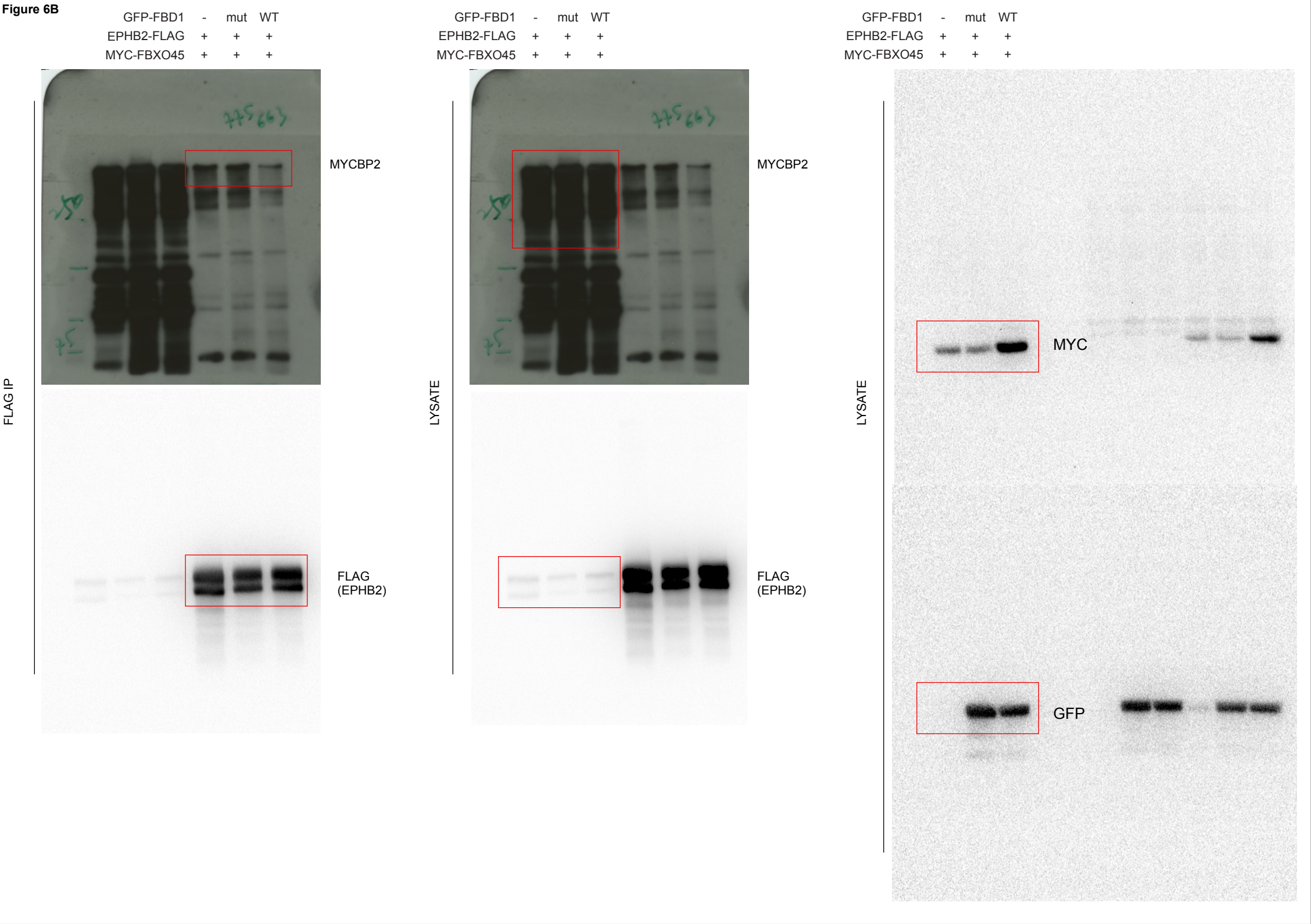

Supplement: Figure 6—source data 1. [file elife-89176-fig6-data1.zip › Figure 6 - source data 1/Figure 6-source data 1.pdf]

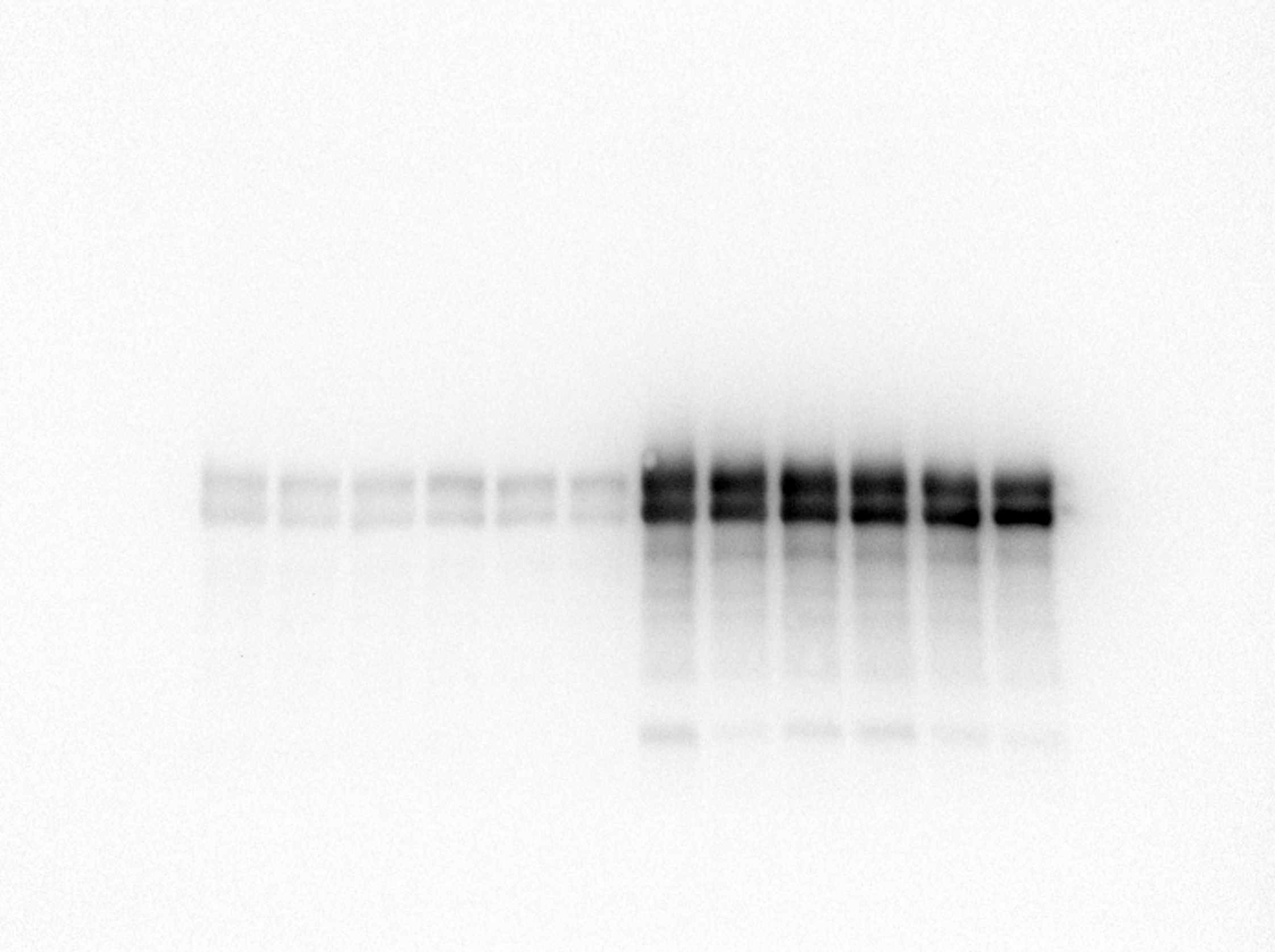

Supplement: Figure 6—source data 2. [file elife-89176-fig6-data2.zip › Figure 6 - source data 2/Figure 6C - FLAG(EPHB2) IP Lysate.tif]

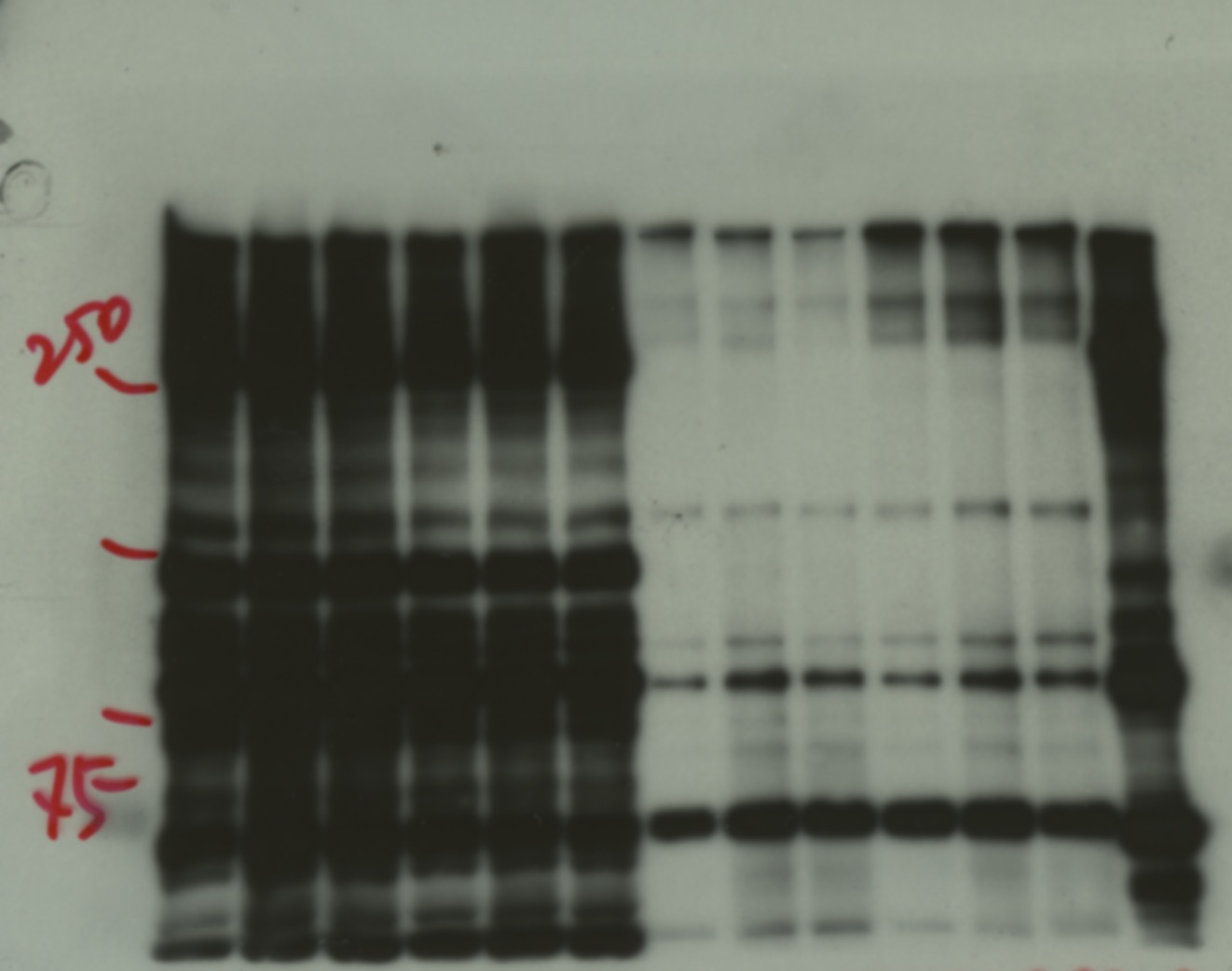

Supplement: Figure 6—source data 2. [file elife-89176-fig6-data2.zip › Figure 6 - source data 2/Figure 6C - MYCBP2 IP Lysate.tif]
