# Supplementary material for: Ubiquitin ligase and signalling hub MYCBP2 is required for efficient EPHB2 tyrosine kinase receptor function
Source: eLife. 2024 Jan 30;12:RP89176. doi: 10.7554/eLife.89176 (PMC10945567; doi:10.7554/eLife.89176)
Supplement: Figure 6—source data 2. [file elife-89176-fig6-data2.zip › Figure 6 - source data 2/Figure 6-source data 2.pdf]

Figure 6C

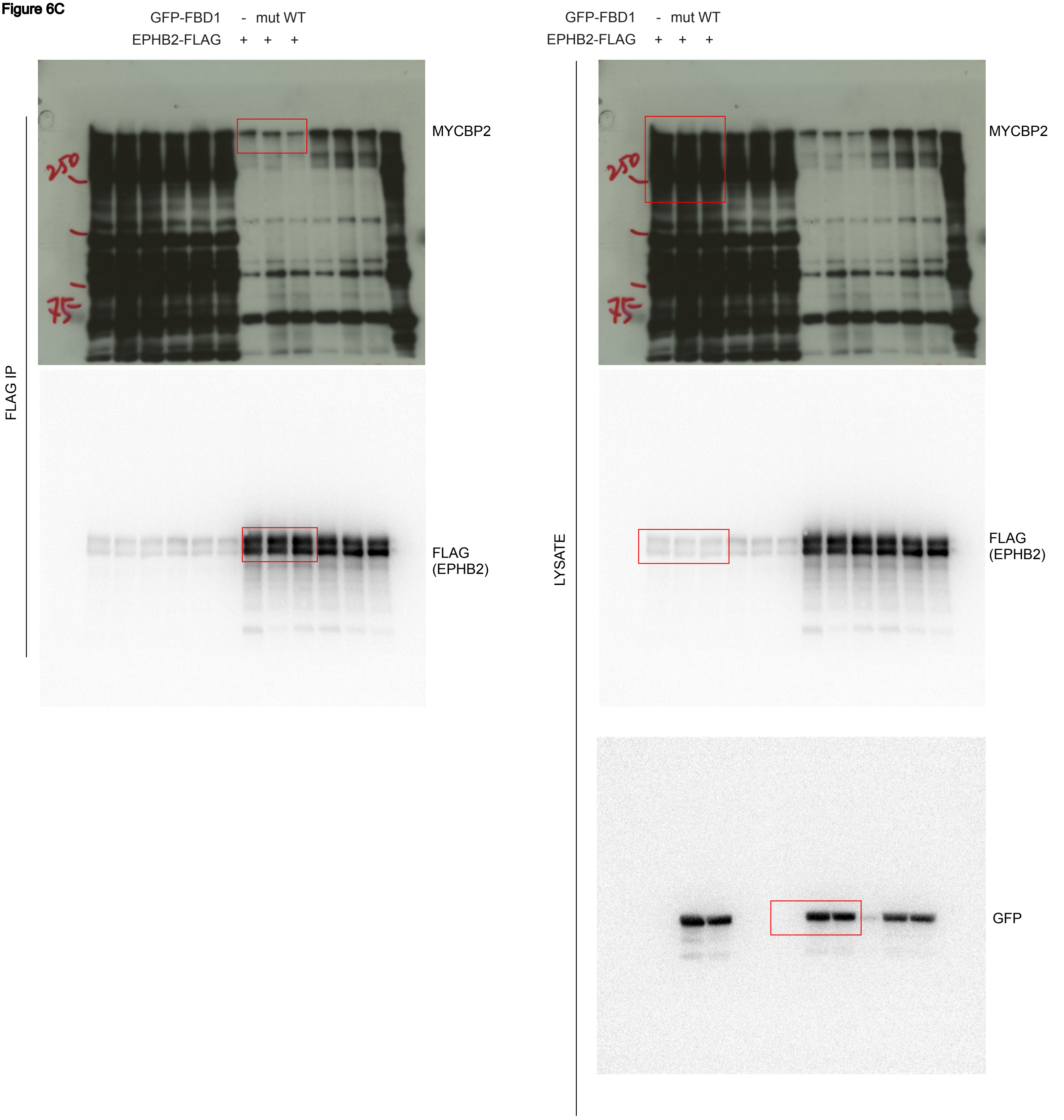
GFP-FBD1 - mut WT
EPHB2-FLAG + + +
MYCBP2
250
75
FLAG (EPHB2)
FLAG IP
GFP-FBD1 - mut WT
EPHB2-FLAG + + +
MYCBP2
250
75
FLAG (EPHB2)
LYSATE
GFP
